# Relationship between 39 dietary markers of ultra-processed food and incident depression: evidence from the prospective UK Biobank cohort study

**DOI:** 10.1007/s00394-026-04084-7

**Published:** 2026-08-21

**Authors:** Annika Katja Blumenthal, Gerrit Eichner, Mathias Fasshauer, Nathalie Judith Eise

**Affiliations:** 1 Institute of Nutritional Science, Justus-Liebig University of Giessen, Goethestr. 55, 35390 Giessen, Germany; 2 Mathematical Institute, Justus-Liebig University of Giessen, 35392 Giessen, Germany; 3 Centre for Sustainable Food Systems, Justus-Liebig University of Giessen, 35390 Giessen, Germany

**Keywords:** Food additives, Markers of ultra-processed food, Incident depression, NOVA classification, Ultra-processed food

## Abstract

**Purpose:**

To evaluate the association between the intake of markers of ultra-processed food (MUPs) and incident depression.

**Methods:**

This population-based, prospective cohort study included 171,818 participants, who completed at least one Oxford WebQ. Cox proportional hazard regression models for incident depression were applied, incorporating the consumption of ten MUP categories and 39 specific MUPs, assessed as percentage of total food intake (%TFI), using penalised cubic splines. The hazard ratio (HR)-nadir was defined as the lowest HR on the %TFI axis and rescaled to 1.

**Results:**

Across an average follow-up duration of 10.7 years, 5424 cases of incident depression occurred. The MUP categories flavour, colouring agent, and sweetener were positively and dose-dependently (all linear *P *< 0.0001) associated with depression risk and the following HRs with corresponding 95% confidence intervals (CIs) were observed as compared to the HR-nadir: flavour (40% vs. 5%TFI; HR (CI) 1.46 (1.29–1.64)), colouring agent (20% vs. 3%TFI; 1.49 (1.30–1.70)), and sweetener (20% vs. 0%TFI; 1.45 (1.32–1.58)). Of the remaining seven MUP categories, two (protein source, fibre) showed a significant U-shaped association and five (flavour enhancer, processing aid, modified starch, varieties of sugar, modified oil) no significant association. Major findings remained robust in various sensitivity analyses.

**Conclusion:**

The results suggest that only certain MUPs are associated with incident depression. These findings offer an initial framework for identifying MUPs that should be prioritised in interventions targeting ultra-processed food consumption and in future mechanistic research.

**Supplementary Information:**

The online version contains supplementary material available at https://doi.org/10.1007/s00394-026-04084-7.

## Background

Ultra-processed foods (UPFs) comprise convenient, ready-to-eat products typically characterised by high contents of fat, salt, and sugar, alongside low levels of dietary fibre, protein, and essential micronutrients [1]. Global per capita UPF sales steadily increased from 2006 to 2019 [2]. According to a systematic review published in 2021, considerable variation in UPF consumption was documented, with the United States of America and the United Kingdom (UK) recording the highest dietary energy contributions from UPF [3]. Convincing evidence from cross-over studies demonstrates that UPF consumption induces body weight gain [4, 5] and impairs weight loss [6]. Furthermore, a recent systematic review with meta-analyses of 92 observational studies conducted between 2016 and 2024 identified robust associations between UPF consumption and adverse health outcomes, including a statistically significant association with depression based on a meta-analysis of six included studies [7]. Depression ranks among the most prevalent psychiatric disorders, affecting approximately 4.0% of the global population and 5.7% of adults worldwide [8]. In 15,960 adults of the NutriNet Brasil cohort study, each 10% increase in the dietary proportion of UPFs was associated with a 10% increase in the hazard ratio (HR) for incident cases of depressive symptoms (HR: 1.10; 95% confidence interval (CI) 1.07–1.14) [9]. Comparable findings were reported in a meta-analysis of six prospective studies conducted by the same authors, in which high versus low UPF exposure was associated with a pooled HR (CI) for depressive outcomes of 1.32 (1.19–1.46) [9].

The NOVA classification is currently the most widely adopted classification system for food processing with UPFs designated as group 4 [1, 10]. A major limitation in current UPF research, however, is the inherent subjectivity stemming from reliance on published food lists for grouping items, leading to potential misclassifications due to vague definitions of UPFs [11]. Consequently, multiple researchers have emphasised the necessity for a clearer and more precise definition of UPFs to facilitate objective health-impact studies [12].

A promising advancement in addressing this issue is the use of pre-defined markers of ultra-processed food (MUPs) which comprise cosmetic additives, such as flavour, colouring agent, and sweetener, and non-culinary ingredients, such as fructose, modified oil, and isolated protein [10, 13]. A food item containing at least one MUP is classified as UPF [10, 13]. This objective and replicable MUP-based method for identifying UPFs was recently applied in extensive analyses involving 22,028 and 11,434 packaged food products from France [14] and Brazil [15], respectively. Using this MUP-based approach, our group recently demonstrated that flavour, emulsifier, and colouring agent are the most frequent markers to detect UPF in the UK food market [16] and that the percentage of UPF items was significantly higher in plant-based meat products as compared to their meat-based counterparts [17]. Furthermore, the MUP categories flavour, flavour enhancer, colouring agent, sweetener, and varieties of sugar were positively linked to all-cause mortality in a recent study by our group [18].

Unlike the broader UPF category [7], no previous studies have assessed the relationship between specific MUPs and depression risk. Addressing this gap is critical because certain MUPs, such as flavour [19], colouring agent [20], and sweetener [21–23] might independently contribute to the negative health impacts associated with UPF consumption, while other specific MUPs may pose minimal or no health risks. Hence, this investigation is the first to systematically analyse the associations of ten MUP categories and 39 specific MUPs with incident depression. Furthermore, this study evaluates the depression risk linked to UPF intake by utilising this MUP-based approach for the first time.

## Methods

### Study design, participants, and exclusion criteria

This analysis was conducted using data from the UK Biobank, a large-scale, prospective cohort study comprising over 500,000 participants [24]. Individuals were recruited across the UK between 2006 and 2010 [24]. For the purposes of the present study, a subset of 210,839 participants was selected, all of whom had completed at least one web-based 24-h dietary recall questionnaire (Oxford WebQ), which records intake data for 206 food items and 32 beverage items consumed on the previous day (Online Resource 1) [25]. The following exclusion criteria were applied: (1) absence of key lifestyle data, specifically physical activity and smoking status; (2) pre-existing depression; (3) missing socio-economic variables, including ethnic background, self-reported general health status, highest educational attainment, and the Townsend deprivation index; (4) absence of clinical examination data, namely body mass index (BMI) and systolic blood pressure (SBP); (5) a prior diagnosis of malabsorption; (6) pre-existing diabetes mellitus; and (7) implausible estimates of energy intake, defined as total energy consumption equal to 0 kcal, < 1.1 × basal metabolic rate − 500 kcal, > 2.5 × basal metabolic rate + 500 kcal, or falling within the upper 0.1% of total energy intake (Online Resource 1). Following the application of exclusion criteria, 171,818 individuals were retained for the primary analyses. Ethical approval for the UK Biobank was obtained from the North West Multi-centre Research Ethics Committee, and written informed consent was provided by all participants at baseline [24].

### Exposure assessment

Assessment of MUP intake was performed similarly as described recently by our group [18]. In brief, for each Oxford WebQ item, up to ten commercial products were researched online from the two market leaders of groceries in the UK, i.e., Tesco and Sainsbury’s. Ingredient lists of 2459 distinct commercial products were analysed concerning the presence of 59 pre-defined MUPs, grouped into ten MUP categories based on recent recommendations (Online Resource 2) [1, 10, 26]. Flavour and colour were identified using the regulatory labelling terminology defined in Regulations (EC) No 1334/2008 [27] and (EU) No 1169/2011 [28]. Several additives such as antioxidants, preservatives, and stabilisers were not considered as MUPs in the present analysis according to recent publications [1, 26].

A Marker Likelihood Index (MLI) was calculated for each of the 238 Oxford WebQ items and each MUP, based on the proportion of commercial products that contained the respective MUP. For example, the MLI for flavour for “chocolate bars” (Oxford WebQ field 102260) was 0.6 since six out of ten commercial products contained flavour. The MLI was then multiplied by the average portion size for each Oxford WebQ item, e.g., given a mean portion size of 50 g for “chocolate bars” and a MLI of 0.6 for flavour, the flavoured portion was calculated to be 30 g. The contribution of each MUP to an individual's total dietary intake (g) was determined by dividing the summed intake of foods containing the respective MUP by the individual's overall food consumption and multiplying this value by 100, resulting in the percentage of total food intake (%TFI). For example, if a participant's intake included 1000 g of foods containing flavour out of an overall dietary intake of 2000 g, the resulting %TFI attributed to flavour would be 50%. This approach was consistently applied across all MUPs. Calculation of %TFI for the UPF group was applied using the same approach. For example, if a participant consumed 1000 g of UPF out of an overall dietary intake of 2000 g, UPF would represent 50%TFI. In cases where participants had completed multiple Oxford WebQs, the mean consumption across these questionnaires was utilised for the primary and sensitivity analyses, except when analyses explicitly focused on data from only the first questionnaire.

### Outcome assessment

Linked morbidity information was provided by UK Biobank, reporting the earliest date of recorded diagnoses, categorised according to three-character codes from the International Statistical Classification of Diseases and Related Health Problems, Tenth Revision (ICD-10) [29]. These morbidity data originated from participant self-reports at the initial assessment, complemented by data obtained from primary care records, inpatient hospital stays, and death certificates [29]. The outcome assessed in this study was the onset of depression, identified through ICD-10 codes F32, i.e., depressive episode, and F33, i.e., recurrent depressive disorder. The duration of follow-up was determined as the interval between the baseline assessment date and the earliest of either depression diagnosis, date of loss to follow-up, death, or end of data acquisition (May 01, 2023), where the latter three led to censored observations. If an individual was diagnosed with both F32 and F33, the earliest diagnostic date was applied.

### Statistical analysis

All statistical analyses and data visualisations were performed using R (version 4.4.2) [30] alongside relevant packages including readxl [31], tidyverse [32], and survival [33]. Multivariable Cox proportional hazard regression models were employed to estimate HRs for incident depression. Intakes of UPFs and MUPs (%TFI), as well as total energy intake, were modelled using penalised cubic splines with four degrees of freedom. Models were adjusted for the following covariates (grouping; source): age (split by quintiles; touchscreen questionnaire), alcohol intake (< 1, 1 to < 8, 8 to < 16, ≥ 16 g per day; touchscreen questionnaire), BMI (< 18.5, 18.5 to < 25, 25 to < 30, ≥ 30 kg/m^2^; physical examination), ethnic background (White versus all other groups; touchscreen questionnaire), general health status (poor, fair, good, excellent; touchscreen questionnaire), highest qualification (none, national exams at age 16, vocational qualifications or optional national exams at 17–18 years, professional, college or university; touchscreen questionnaire), household income (< £18k, £18k to < £31k, £31k to < £52k, £52k to < £100k, ≥ £100k, unknown; touchscreen questionnaire), physical activity (Metabolic Equivalent of Task (MET) per week, split by quintiles; Oxford WebQ), SBP (split by quintiles; physical examination), sex (female, male; central registry at recruitment and in some cases updated by the participant), smoking status (never, previous, current occasional, current < 10, 10–14, 15–19, ≥ 20 cigarettes per day; touchscreen questionnaire), and the Townsend deprivation index (split by quintiles; national census data). Variables that violated the proportional hazard assumption as assessed via scaled Schoenfeld residuals were stratified in the final models. For all analyses, the HR-nadir defined as the lowest HR observed between 0 and the 99th percentile of %TFI was rescaled to 1 to facilitate comparison across intake levels. HRs are presented with corresponding 95% CI. Linear and non-linear associations were evaluated separately and a *P* < 0.05 was considered statistically significant. Non-significant findings in the primary models were not further interpreted and in the case of specific MUPs excluded from sensitivity analyses.

### Sensitivity analyses

To test the robustness of the findings, several sensitivity analyses were conducted: (1) exclusion of participants with follow-up durations shorter than two years to mitigate reverse causality (landmark analysis); (2) exclusion of individuals reporting unintentional weight loss, a possible indicator of underlying disease; (3) exclusion of participants who reported at least once that their previous day’s diet was not typical to exclude non-representative dietary data; (4) exclusion of participants who completed only a single Oxford WebQ, addressing potential lower reproducibility in UPF and MUP consumption; (5) exclusion of individuals with pre-existing cardiovascular disease or cancer, since they might be prone to an increased depression risk; (6) assessment of UPF and MUP intake based solely on the first Oxford WebQ since it was filled out most adjacent to the baseline assessment; (7) additional adjustment for a diet quality score to explore potential confounding; (8) substitution of BMI with waist-to-hip ratio and height to more accurately account for adiposity distribution; and (9) exclusion of total energy intake from the model to account for its potential role as a mediator. Additional analyses were conducted in which the main and all sensitivity analyses were further adjusted for multiple testing using the Benjamini–Hochberg method.

## Results

### Baseline data of UK Biobank participants

The baseline characteristics of the overall study population, as well as those of the subgroups defined by UPF intake quintiles, are detailed in Table 1 and Online Resource 3. The study population had a mean (standard deviation (SD)) age of 58.5 (8.0) years, with UPF intake comprising 19.9 (8.8) %TFI (Table 1). Among the MUP categories, the highest mean (SD) %TFI was observed for flavour at 13.5 (8.2), followed by processing aid at 7.6 (3.5), and colouring agent at 5.6 (4.0) (Table 1). Throughout a mean follow-up of 10.7 (1.8) years and 1.8 million person-years, 5424 incident depression cases were recorded.

**Table 1:** Baseline characteristics of the UK Biobank cohort^a^

Parameters	Total cohort (n = 171,818)	Quintiles of UPF intake (%TFI)				
		0.4 to 12.9 (n = 34,364)	12.9 to 16.5 (n = 34,363)	16.5 to 20.3 (n = 34,364)	20.3 to 25.9 (n = 34,363)	25.9 to 98.7 (n = 34,364)
Age (years)	58.5 (8.0)	58.3 (7.9)	59.0 (7.8)	59.0 (7.9)	58.7 (8.1)	57.4 (8.4)
Alcohol (g per day)						
< 1	60,447 (35.2)	8227 (23.9)	9866 (28.7)	11,279 (32.8)	13,386 (39.0)	17,689 (51.5)
1 to < 8	18,449 (10.7)	2217 (6.5)	3448 (10.0)	4174 (12.1)	4537 (13.2)	4073 (11.9)
8 to < 16	24,419 (14.2)	3906 (11.4)	5145 (15.0)	5440 (15.8)	5425 (15.8)	4503 (13.1)
≥ 16	68,503 (39.9)	20,014 (58.2)	15,904 (46.3)	13,471 (39.2)	11,015 (32.1)	8099 (23.6)
BMI (kg/m^2^)						
< 18.5	972 (0.6)	209 (0.6)	184 (0.5)	195 (0.6)	224 (0.7)	160 (0.5)
18.5 to < 25	67,855 (39.5)	14,263 (41.5)	14,301 (41.6)	13,952 (40.6)	13,338 (38.8)	12,001 (34.9)
25 to < 30	72,236 (42.0)	14,479 (42.1)	14,425 (42.0)	14,456 (42.1)	14,441 (42.0)	14,435 (42.0)
≥ 30	30,755 (17.9)	5413 (15.8)	5453 (15.9)	5761 (16.8)	6360 (18.5)	7768 (22.6)
Energy (kJ)	8918 (2240)	8363 (2146)	8805 (2109)	9015 (2163)	9204 (2253)	9203 (2406)
Ethnic background						
White	165,443 (96.3)	33,019 (96.1)	33,247 (96.8)	33,258 (96.8)	33,116 (96.4)	32,803 (95.5)
Group composed of Mixed, Asian, Black, Chinese, and other	6375 (3.7)	1345 (3.9)	1116 (3.2)	1106 (3.2)	1247 (3.6)	1561 (4.5)
General health status						
Poor	3391 (2.0)	582 (1.7)	575 (1.7)	573 (1.7)	694 (2.0)	967 (2.8)
Fair	25,899 (15.1)	4564 (13.3)	4734 (13.8)	4908 (14.3)	5340 (15.5)	6353 (18.5)
Good	105,277 (61.3)	20,857 (60.7)	21,106 (61.4)	21,328 (62.1)	21,280 (61.9)	20,706 (60.3)
Excellent	37,251 (21.7)	8361 (24.3)	7948 (23.1)	7555 (22.0)	7049 (20.5)	6338 (18.4)
Highest qualification						
None of the below	13,755 (8.0)	2415 (7.0)	2576 (7.5)	2648 (7.7)	2860 (8.3)	3256 (9.5)
National exams at age 16 years	25,811 (15.0)	4936 (14.4)	4916 (14.3)	4950 (14.4)	5206 (15.2)	5803 (16.9)
Vocational qualifications or optional national exams at ages 17–18 years	30,411 (17.7)	5846 (17.0)	5769 (16.8)	5901 (17.2)	6172 (18.0)	6723 (19.6)
Professional	26,633 (15.5)	5070 (14.8)	5173 (15.1)	5388 (15.7)	5567 (16.2)	5435 (15.8)
College or university	75,208 (43.8)	16,097 (46.8)	15,929 (46.4)	15,477 (45.0)	14,558 (42.4)	13,147 (38.3)
Household income per year (k£)						
< 18	21,886 (12.7)	3621 (10.5)	3982 (11.6)	4335 (12.6)	4701 (13.7)	5247 (15.3)
18 to < 31	37,291 (21.7)	6605 (19.2)	7282 (21.2)	7630 (22.2)	7852 (22.9)	7922 (23.1)
31 to < 52	44,612 (26.0)	8658 (25.2)	8854 (25.8)	9078 (26.4)	8999 (26.2)	9023 (26.3)
52 to < 100	39,224 (22.8)	8831 (25.7)	8166 (23.8)	7812 (22.7)	7415 (21.6)	7000 (20.4)
≥ 100	11,832 (6.9)	3411 (9.9)	2688 (7.8)	2196 (6.4)	1915 (5.6)	1622 (4.7)
Unknown	16,973 (9.9)	3238 (9.4)	3391 (9.9)	3313 (9.6)	3481 (10.1)	3550 (10.3)
Physical activity (MET per week)	4146 (2655)	4196 (2723)	4198 (2606)	4159 (2588)	4116 (2603)	4061 (2750)
SBP (mmHg)	138.8 (19.5)	139.4 (19.8)	139.1 (19.5)	139.0 (19.4)	138.7 (19.4)	137.9 (19.3)
Sex						
Female	96,772 (56.3)	19,493 (56.7)	19,705 (57.3)	19,447 (56.6)	19,059 (55.5)	19,068 (55.5)
Male	75,046 (43.7)	14,871 (43.3)	14,658 (42.7)	14,917 (43.4)	15,304 (44.5)	15,296 (44.5)
Smoking status						
Never	99,631 (58.0)	18,144 (52.8)	19,701 (57.3)	20,267 (59.0)	20,614 (60.0)	20,905 (60.8)
Previous	60,380 (35.1)	13,354 (38.9)	12,354 (36.0)	11,950 (34.8)	11,616 (33.8)	11,106 (32.3)
Current occasional	4030 (2.3)	1013 (2.9)	833 (2.4)	756 (2.2)	719 (2.1)	709 (2.1)
< 10 cigarettes per day	2073 (1.2)	515 (1.5)	390 (1.1)	399 (1.2)	371 (1.1)	398 (1.2)
10–14 cigarettes per day	1744 (1.0)	423 (1.2)	316 (0.9)	315 (0.9)	322 (0.9)	368 (1.1)
15–19 cigarettes per day	1543 (0.9)	341 (1.0)	323 (0.9)	272 (0.8)	283 (0.8)	324 (0.9)
≥ 20 cigarettes per day	2417 (1.4)	574 (1.7)	446 (1.3)	405 (1.2)	438 (1.3)	554 (1.6)
Townsend deprivation index	−1.7 (2.8)	−1.6 (2.9)	−1.7 (2.8)	−1.8 (2.8)	−1.7 (2.8)	−1.6 (2.9)
UPF intake (%TFI)	19.9 (8.8)	10.2 (2.0)	14.7 (1.0)	18.3 (1.1)	22.8 (1.6)	33.6 (7.8)
MUP category intake (%TFI)						
Flavour	13.5 (8.2)	5.7 (1.9)	8.9 (2.0)	11.7 (2.4)	15.6 (3.0)	25.8 (8.3)
Flavour enhancer	0.3 (0.5)	0.2 (0.4)	0.3 (0.4)	0.3 (0.5)	0.4 (0.6)	0.4 (0.7)
Colouring agent	5.6 (4.0)	2.4 (1.2)	3.7 (1.5)	4.9 (1.9)	6.5 (2.5)	10.5 (5.3)
Sweetener	4.5 (5.9)	0.8 (1.1)	1.8 (1.8)	3.0 (2.6)	4.9 (3.7)	12.0 (8.5)
Processing aid	7.6 (3.5)	4.6 (1.7)	6.4 (2.0)	7.5 (2.4)	8.8 (3.0)	10.6 (4.3)
Modified starch	0.5 (0.5)	0.3 (0.3)	0.4 (0.4)	0.5 (0.5)	0.6 (0.5)	0.7 (0.7)
Varieties of sugar	5.0 (2.6)	2.9 (1.2)	4.0 (1.4)	4.8 (1.7)	5.7 (2.1)	7.4 (3.2)
Modified oil	0.0 (0.1)	0.0 (0.0)	0.0 (0.1)	0.0 (0.1)	0.1 (0.1)	0.1 (0.1)
Protein source	2.7 (1.9)	1.6 (1.0)	2.3 (1.2)	2.7 (1.5)	3.2 (1.8)	3.8 (2.6)
Fibre	1.0 (0.9)	0.6 (0.5)	0.8 (0.7)	1.0 (0.8)	1.2 (1.0)	1.4 (1.3)

^a^Categorical variables are summarised as frequencies (percentages) and continuous variables as mean (SD). %TFI, Percentage total food intake; BMI, Body mass index; MET, Metabolic equivalent of task; MUP, Marker of ultra-processed food; SBP, Systolic blood pressure; SD Standard deviation; UK, United Kingdom; UPF, Ultra-processed food

### Main analyses

#### Association of UPF and ten MUP categories with incident depression

**Fig. 1:**
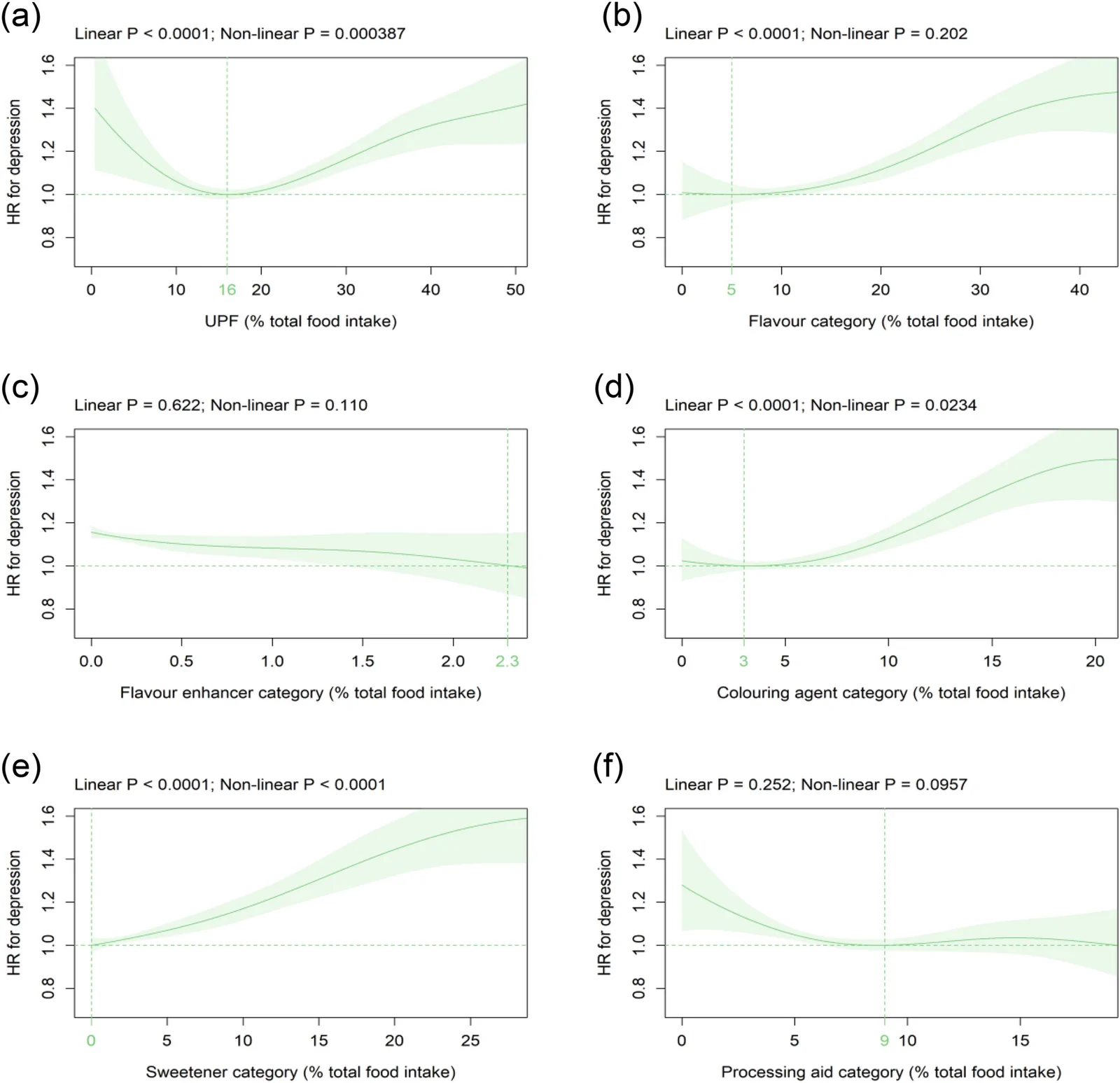

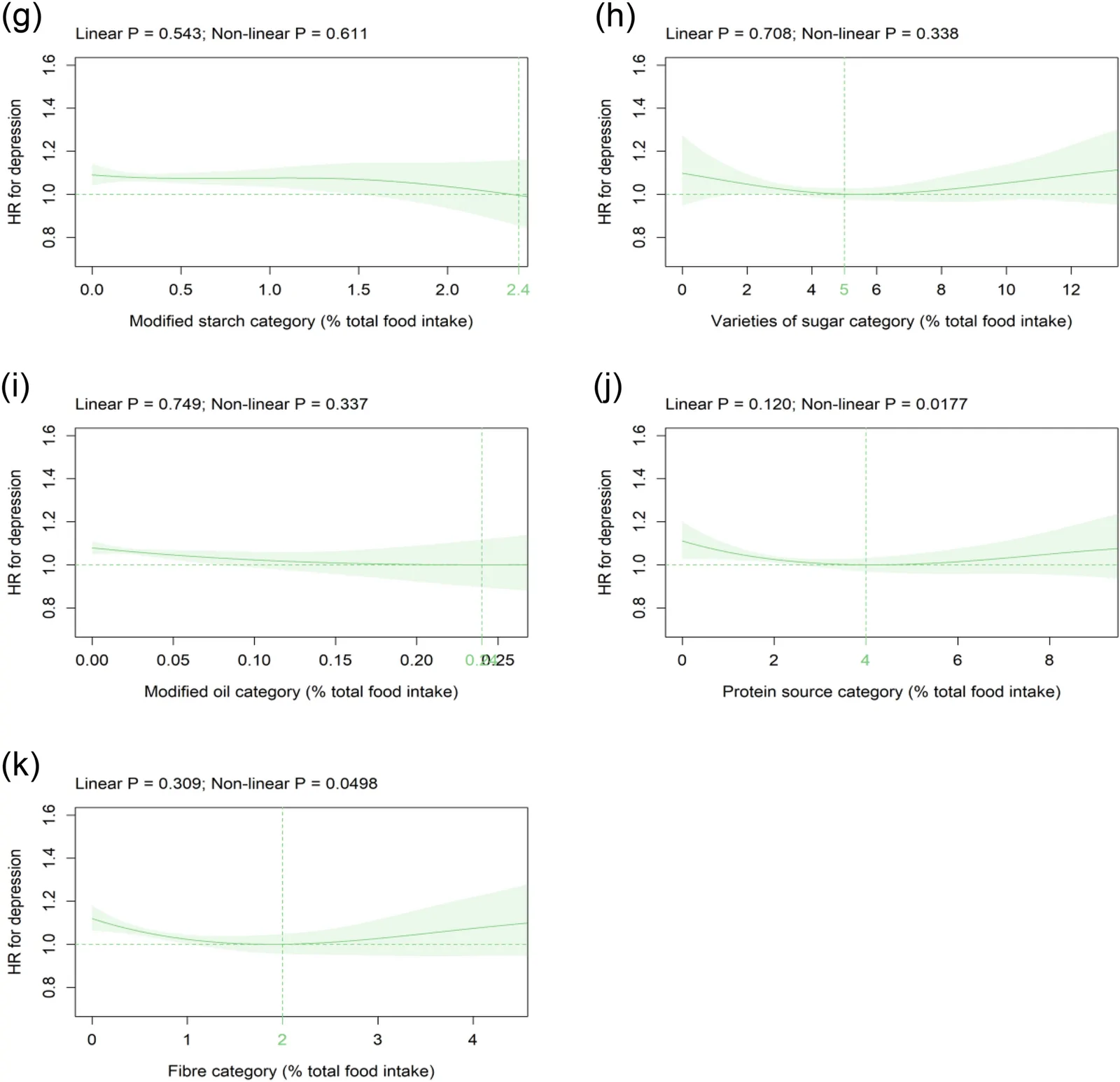
Association of depression risk with UPF and MUP categories. Association of depression risk with **a** UPF, as well as the MUP categories (all %TFI) **b** flavour, **c** flavour enhancer, **d** colouring agent, **e** sweetener, **f** processing aid, **g** modified starch, **h** varieties of sugar, **i** modified oil, **j** protein source, and **k** fibre (n = 171,818; number of events = 5424). Models are adjusted for age, alcohol intake, BMI, energy intake, ethnic background, general health status, highest qualification, household income, physical activity, sex, smoking status, SBP, and Townsend deprivation index as indicated in the Methods section. Abbreviations: %TFI, Percentage total food intake; BMI, Body mass index; HR, Hazard ratio; MUP, Marker of ultra-processed food; SBP, Systolic blood pressure; UPF, Ultra-processed food

A significant U-shaped association between UPF consumption and incident depression was identified, with the HR-nadir occurring at 16%TFI, beyond which the risk of depression rose linearly (Fig. 1a). Compared to the intake level at the HR-nadir, the mean (pointwise 95% CI) HR increased to 1.40 (1.11–1.76), 1.16 (1.11–1.22), 1.32 (1.22–1.43), and 1.41 (1.23–1.61) at 0, 30, 40, and 50%TFI, respectively (Fig. 1a). Within the flavour category, the risk of incident depression demonstrated a significant linear increase from the HR-nadir at 5%TFI, with HRs reaching 1.12 (1.07–1.16), 1.32 (1.22–1.42), and 1.46 (1.29–1.64) at 20, 30, and 40%TFI, respectively (Fig. 1b). Colouring agent intake was also linearly associated with depression risk beyond the HR-nadir at 3%TFI, with HR rising to 1.13 (1.08–1.18) at 10%TFI and 1.49 (1.30–1.70) at 20%TFI (Fig. 1d). A similar linear association with a HR-nadir at 0%TFI was observed for sweetener, with HRs of 1.17 (1.12–1.23) and 1.45 (1.32–1.58) at 10 and 20%TFI, respectively (Fig. 1e). In contrast, protein source (Fig. 1j) and fibre (Fig. 1k) showed a small but significant non-linear association with incident depression with increased HRs of 1.11 (1.03–1.20) and 1.12 (1.06–1.18) in non-consumers as compared to the HR-nadirs at 4%TFI and 2%TFI, respectively. No statistically significant associations with depression risk were detected for the MUP categories flavour enhancer (Fig. 1c), processing aid (Fig. 1f), modified starch (Fig. 1g), varieties of sugar (Fig. 1h), and modified oil (Fig. 1i).

#### Association of specific MUPs with depression risk

**Fig. 2:**
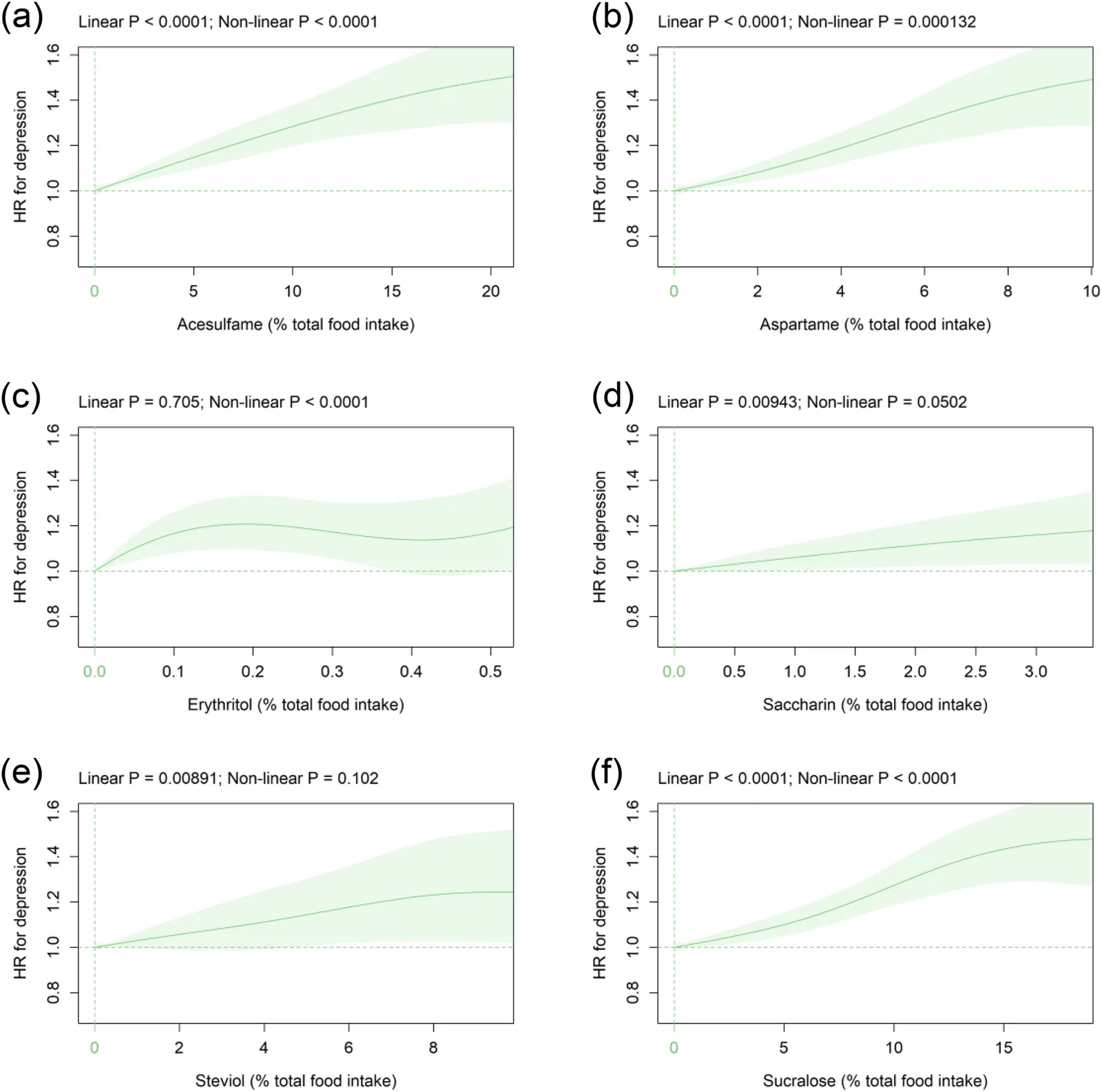

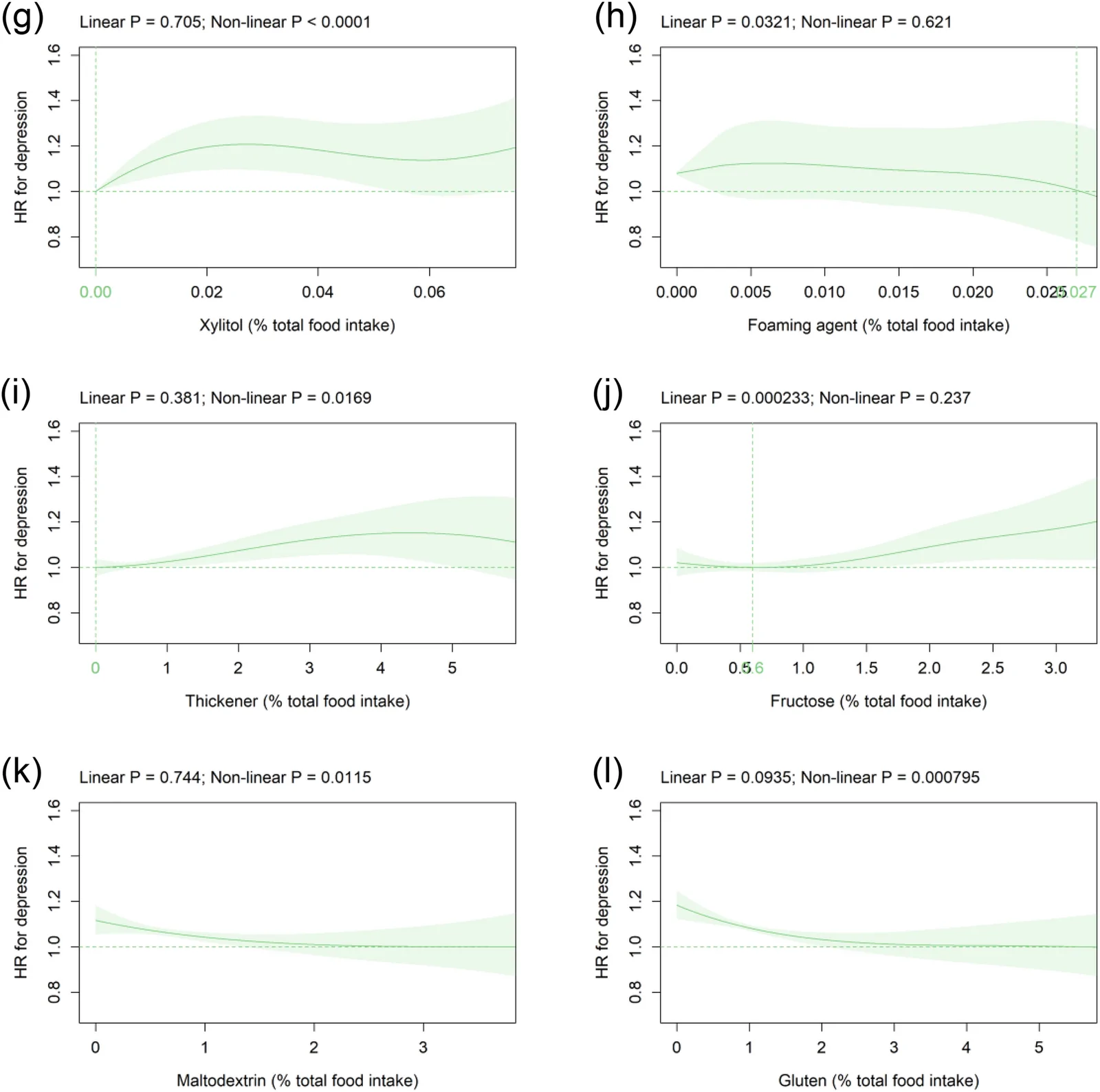
Association of depression risk with specific MUPs. Association of depression risk with the specific MUPs (all %TFI) **a** acesulfame, **b** aspartame, **c** erythritol, **d** saccharin, **e** steviol, **f** sucralose, **g** xylitol, **h** foaming agent, **i** thickener, **j** fructose, **k** maltodextrin, and **l** gluten (n = 171,818; number of events = 5424). Models are adjusted as defined in Fig. 1 and abbreviations are indicated in Fig. 1

Of the 39 specific MUPs identified in at least one food product, five MUPs, i.e., flavour, colour, modified starch, hydrogenated oil, and fibre, were equivalent to their corresponding MUP categories (Online Resource 2), and these have already been depicted in Fig. 1b (flavour category corresponding to flavour), 1d (colouring agent category corresponding to colour), 1g (modified starch category corresponding to modified starch), 1i (modified oil category corresponding to hydrogenated oil), and 1k (fibre category corresponding to fibre). Of the other 34 specific MUPs analysed individually, 12 demonstrated significant associations with the risk of depression (Fig. 2). Within the sweetener category, an HR-nadir at 0%TFI and significant dose-dependent relationships with depression risk were identified for the following seven out of ten MUPs: acesulfame (Fig. 2a), aspartame (Fig. 2b), erythritol (Fig. 2c), saccharin (Fig. 2d), steviol (Fig. 2e), sucralose (Fig. 2f), and xylitol (Fig. 2g). In addition fructose (Fig. 2j) was associated with incident depression in a linear fashion. In contrast, a non-linear relation was observed for thickener intake, with an HR-nadir at 0%TFI (Fig. 2i). The MUPs foaming agent (Fig. 2h), maltodextrin (Fig. 2k), and gluten (Fig. 2l) showed an inverse association. The remaining 22 specific MUPs did not display significant associations with depression risk including glutamate, emulsifier, and inverted sugar (Online Resource 4).

### Sensitivity analyses

Major results of all sensitivity analyses are summarised in Online Resource 5. The associations between UPF intake and depression risk (Online Resource 6a to 14a), as well as those for the categories flavour (Online Resource 6b to 14b), colouring agent (Online Resource 6d to 14d), and sweetener (Online Resource 6e to 14e), remained significant in all sensitivity analyses. For the categories protein source (Online Resource 6j to 14j) and fibre (Online Resource 6k to 14k) five and four of the nine sensitivity analyses remained significant, respectively. Of the 12 specific MUPs which were significantly associated with depression risk in the main analyses (Fig. 2), acesulfame (Online Resource 6l to 14l), aspartame (Online Resource 6m to 14m), erythritol (Online Resource 6n to 14n), sucralose (Online Resource 6q to 14q), xylitol (Online Resource 6r to 14r), and fructose (Online Resource 6u to 14u) remained significant in all sensitivity analyses. Associations of all other MUPs were significant in the majority of all sensitivity analyses (Online Resource 5 to 14). Associations with depression risk in the main analysis remained significant after adjustment for multiple testing for the MUP categories flavour, colouring agent, and sweetener, as well as for all specific MUPs except for foaming agent, thickener, and maltodextrin (Online Resource 15).

## Discussion

The current study evaluates for the first time the associations of all MUP categories, as well as specific MUPs, with depression risk. The results suggest that the MUP categories flavour, colouring agent, and sweetener are most prominently linked with incident depression. It is interesting to note in this context, that these three MUP categories were also positively associated with all-cause mortality in a recent study by our group [18].

To the best of our knowledge, our study is the first to suggest a link between flavour and colouring agent with incident depression. However, both MUPs have been implicated in other adverse health outcomes. In fact, our research group has previously reported that added flavour significantly contributes to increased body weight and obesity by mechanisms involving enhanced hedonic eating and disrupted flavour-nutrient learning [19]. Colouring agent on the other hand has been implicated in adverse health outcomes in children and adolescents, including Attention Deficit Hyperactivity Disorder and Autism Spectrum Disorder [20]. The current findings are in accordance with the hypothesis that flavour and colouring agent might be linked to depression risk in addition to these other known adverse effects [19, 20]. It is noteworthy that flavour and colouring agent represent the most and third most frequent MUPs in a recent UK market analysis from our group, detecting 58.4% and 26.5% of all UPFs, respectively [16].

In the current study, sweetener intake is dose-dependently associated with depression risk. Within the sweetener category, an HR-nadir at 0%TFI and significant dose-dependent relationships with incident depression are identified for seven out of ten MUPs. These findings are supported by results from independent groups. Thus, healthy adults who consume a high-aspartame diet for eight days exhibit more depression and more irritable mood as compared to a low-aspartame diet [34]. Comparing extreme quintiles, artificially sweetened beverages and artificial sweeteners are the only UPF components significantly and independently associated with greater risk of depression in a cohort including 31,712 females from the Nurses’ Health Study II [21]. Moreover, increasing consumption of sweetener-based diet drinks is associated with an increased risk for depression in 263,923 participants of the NIH-AARP Diet and Health Study [22]. Similarly, a significant and convincing association between depression and consumption of sweeteners and diet drinks is found in 18,838 participants from Atlantic Canada [23]. It is interesting to note in this context that the use of artificial sweeteners in the UK soft drinks market has likely increased following the introduction of the sugar tax [35, 36]. Since younger adults consume higher amounts of artificially sweetened beverages [37], the potential long-term effects of this increased sweetener exposure on mental health in general and depression in particular should be assessed in future studies.

Besides the MUP categories flavour, colouring agent, and sweetener, the specific MUP fructose is dose-dependently related to incident depression. Excessive fructose intake is known to negatively impact metabolic health through mechanisms involving hepatic lipogenesis, insulin resistance, and disruptions in leptin and ghrelin regulation, contributing to increased food intake [38]. In rats, a high-fat, high-fructose diet induces metabolic derangements and depression- and anxiety-like behaviours [39]. However, it remains unclear whether early-life fructose exposure affects the risk of depression-like behaviours or anxiety [40]. Therefore, further work is needed to define the role of fructose consumption in the pathophysiology of depression.

Our study is the first to show that thickener intake is significantly associated with depression risk. In this context, it is noteworthy that carboxymethylcellulose and carrageenan, which are commonly used as thickeners and emulsifiers, have been shown to adversely affect the gut microbiota [41, 42] and such alterations may contribute to the pathogenesis of depression [43, 44].

Furthermore, the MUP categories protein source and fibre, along with the specific MUPs foaming agent, maltodextrin, and gluten, show significant associations with depression risk. Since all these MUPs show the highest risk for depression in non-consumers, there is no evidence of harm at higher intake levels in contrast to the MUP categories flavour, colouring agent, and sweetener.

The mechanisms underlying the observed associations between specific MUPs and depression remain to be elucidated. Potential mechanisms include direct effects of specific MUPs on brain function and neurochemical signalling, which may contribute to depression as a neurobiological disorder [45]. Additional pathways may involve indirect effects mediated through obesity, metabolic dysfunction, and alterations of the gut microbiota [46]. Further insight into these pathways will require both animal experiments and human intervention studies incorporating neuroimaging approaches, gut microbiome analyses, metabolic biomarker profiling, and assessments of depressive symptoms.

Our investigation applies for the first time a MUP-based definition of UPF, identifying a significant U-shaped association with depression risk. The linear risk increased beyond 16%TFI broadly aligns with earlier studies employing conventional UPF definitions derived from established food lists. For instance, a recent systematic review with meta-analyses links UPF intake to a wide range of adverse health outcomes, including increased depression risk [7]. The significantly increased depression risk in non-UPF consumers further supports the notion that only part of the MUP categories and specific MUPs are positively and dose-dependently associated with depression risk and further studies should focus on these candidates.

A key strength of the current study lies in its analysis of pre-defined MUP categories and specific MUPs within a large, well-characterised prospective cohort, featuring a 10.7-year mean follow-up, wide exposure ranges, and advanced modelling to capture potential non-linear associations. This study has limitations, including potential residual confounding, self-reported dietary data without independent validation, and inaccuracies in exposure measurement. The Oxford WebQ does not assess ingredient lists directly, thus, MLI scores reflect estimated probabilities of MUP presence. While this introduces some uncertainty, the MUP-based approach offers greater objectivity and reproducibility than traditional UPF classifications based on food lists. Furthermore, reverse causality cannot be excluded since people with depression might face listlessness potentially leading to a greater reliance on ready-to-eat meals rather than preparing meals from scratch. It is interesting to note in this context that depression severity, as assessed by the Patient Health Questionnaire−9, is positively associated with the frequency of ready-to-eat food consumption in a recent cross-sectional analysis of the National Health and Nutrition Examination Survey [47]. Nevertheless, all associations except for maltodextrin remain significant after exclusion of participants with a follow-up duration shorter than 2 years (landmark analysis). Finally, a “healthy volunteer” bias may limit generalisability, although full population representativeness is not essential for valid exposure-outcome associations [48].

## Conclusions

The present study is the first to comprehensively examine both MUP categories and specific MUPs in relation to depression risk. The results suggest that the MUP categories flavour, colouring agent, and sweetener are most prominently and dose-dependently associated with incident depression, while others show no association with this outcome. These findings offer an initial framework for identifying MUPs that should be prioritised in interventions targeting UPF consumption and in future mechanistic research. Furthermore, the results support the refinement of current UPF definitions by focusing on those MUPs most strongly linked to adverse health effects. Future studies should aim to replicate the associations between MUPs and depression observed using the MLI in this study, investigate relationships with other health outcomes, and further explore the underlying biological mechanisms.

## Supplementary Information

Below is the link to the electronic supplementary material.

Supplementary file1 (PDF 7358 KB)

## Data Availability

Data described in the manuscript, code book, and analytic code will not be made available because the data used in this study were accessed under institutional data use agreements with the UK Biobank (application number 53438) and these agreements do not permit public sharing of the data. However, researchers can apply for access directly through the UK Biobank (www.ukbiobank.ac.uk) in accordance with their access procedures.

## Abbreviations

%TFI: Percentage total food intake
BMI: Body mass index
CI: Confidence interval
HR: Hazard ratio
MET: Metabolic equivalent of task
MLI: Markers Likelihood Index
MUP: Marker of ultra-processed food
SBP: Systolic blood pressure
SD: Standard deviation
UK: United Kingdom
UPF: Ultra-processed food

## Declarations

### Generative AI and AI-assisted technologies in the writing process

During the preparation of this work the authors used ChatGPT (OpenAI, version GPT-5.5) in order to assist with language editing and to improve grammar, clarity, and style. No scientific content, data analysis, or interpretation was generated or influenced by the use of the tool. After using this tool/service, the authors reviewed and edited the content as needed and take full responsibility for the content of the publication.
