# Supplementary material for: Relationship between 39 dietary markers of ultra-processed food and incident depression: evidence from the prospective UK Biobank cohort study
Source: Eur J Nutr. 2026 Aug 21;65(6):238. doi: 10.1007/s00394-026-04084-7 (PMC13498484; doi:10.1007/s00394-026-04084-7)
Supplement: Supplementary file 1 [file 394_2026_4084_MOESM1_ESM.pdf]

Online Resources

Relationship between 39 dietary markers of ultra-processed food and incident depression: evidence from the prospective UK Biobank cohort study

Annika Katja Blumenthal, M. Sc. (0009-0007-2306-2182)[1*], Gerrit Eichner, PhD (0000-0001-9523-5520)[2*], Mathias Fasshauer, MD (0000-0001-8077-6404)[1,3#], Nathalie Judith Eise, PhD (0000-0003-3247-1555)[1#§]

1. Institute of Nutritional Science, Justus-Liebig University of Giessen, 35390 Giessen, Germany.
2. Mathematical Institute, Justus-Liebig University of Giessen, 35392 Giessen, Germany.
3. Centre for Sustainable Food Systems, Justus-Liebig University of Giessen, 35390 Giessen, Germany.

[*]AKB and GE contributed equally to this work and are joint first authors.
[#]MF and NJE contributed equally to this work and are joint senior authors.
[§]Corresponding author: Nathalie Judith Eise. Mailing address: Goethestr. 55, 35390 Giessen, Germany. E-mail: nathalie.eise@uni-giessen.de. Phone: +49 641 9939067. Fax: +49 641 9939069.

Index

Online Resource 1

Flowchart of participant selection

Exclusion criteria included (1) missing lifestyle factors, i.e., physical activity and smoking status, (2) pre-existing depression, (3) missing socio-economic factors, i.e., ethnic background, general health status, highest qualification, and Townsend deprivation index, (4) missing exam parameters, i.e., body mass index and systolic blood pressure, (5) pre-existing malabsorption, (6) pre-existing diabetes mellitus, and (7) implausible energy intake, i.e., total energy intake of 0 kcal or <(1.1 × basal metabolic rate - 500 kcal) or >(2.5 × basal metabolic rate + 500 kcal) or belonging to the top 0.1 % total energy intake. Abbreviation: OWQ, Oxford WebQ

Online Resource 2

Systematic overview of specific MUPs and their individual compounds

Specific MUPs and their individual compounds within the ten categories: flavour, flavour enhancer, colouring agent, sweetener, processing aid, modified starch, varieties of sugar, modified oil, protein source, and fibre. Abbreviations: MSG, Monosodium glutamate; MUP, Marker of ultra-processed food

Online Resource 3

Specific MUPs at baseline

Intake of specific MUPs at baseline in both the total cohort and the subgroups based on quintiles of UPF intake (%TFI). Abbreviations: TFI, Percentage total food intake; MUP, Marker of ultra-processed food; SD, Standard deviation; UPF, Ultra-processed food

Online Resource 4

Non-significant associations of specific MUPs (all %TFI) with depression risk.

Association of depression risk with (a) glutamate, (b) guanylate, (c) inosinate, (d) ribonucleotide, (e) isomalt, (f) maltitol, (g) sorbitol, (h) bulking agent, (i) caking agent, (j) emulsifier, (k) firming agent, (l) gelling agent, (m) glazing agent, (n) humectant, (o) sequestrant, (p) dextrose, (q) inverted sugar, (r) juice concentrate, (s) lactose, (t) hydrolysed protein, (u) isolated protein, and (v) whey protein (n=171,818; number of

events=5,424). Models are adjusted as defined in Figure 1 and abbreviations are indicated in Figure 1

Online Resource 5

Overview of sensitivity analyses

Association of UPF, MUP categories, and specific MUPs with incident depression in all sensitivity analyses. Results are only shown for associations which were significant in the main analyses. The respective Online Resources are indicated (e.g., Landmark analysis for colouring agent is depicted in Online Resource 6d). Abbreviations: BMI, Body mass index; CVD, Cardiovascular disease; MUP, Marker of ultra-processed food; ns, Non-significant; s, Significant, UPF, Ultra-processed food; WHR, Waist-to-hip ratio

Online Resource 6

Landmark analysis

Association of depression risk with (a) UPF, the MUP categories (b) flavour, (c) flavour enhancer, (d) colouring agent, (e) sweetener, (f) processing aid, (g) modified starch, (h) varieties of sugar, (i) modified oil, (j) protein source, (k) fibre, and the specific MUPs (l) acesulfame, (m) aspartame, (n) erythritol, (o) saccharin, (p) steviol, (q) sucralose, (r) xylitol, (s) foaming agent, (t) thickener, (u) fructose, (v) maltodextrin, and (w) gluten (Landmark analysis; n=169,860; number of events=4,632). Models are adjusted as defined in Figure 1 and abbreviations are indicated in Figure 1

Online Resource 7

Unintentional weight loss removed

Association of depression risk with (a) UPF, (b) to (k) MUPs categories, and (l) to (w) specific MUPs indicated in Online Resource 6 (n=145,116; number of events=4,383). Models are adjusted as defined in Figure 1 and abbreviations are indicated in Figure 1

Online Resource 8

Atypical diet excluded

Association of depression risk with (a) UPF, (b) to (k) MUPs categories, and (l) to (w) specific MUPs indicated in Online Resource 6 (n=115,951; number of events=3,749).

Models are adjusted as defined in Figure 1 and abbreviations are indicated in Figure 1

Online Resource 9

More than one Oxford WebQ

Association of depression risk with (a) UPF, (b) to (k) MUPs categories, and (l) to (w) specific MUPs indicated in Online Resource 6 (n=106,389; number of events=2,922). Models are adjusted as defined in Figure 1 and abbreviations are indicated in Figure 1

Online Resource 10

History of CVD and cancer excluded

Association of depression risk with (a) UPF, (b) to (k) MUPs categories, and (l) to (w) specific MUPs indicated in Online Resource 6 (n=151,869; number of events=4,593). Models are adjusted as defined in Figure 1 and abbreviations are indicated in Figure 1. Further abbreviation: CVD, Cardiovascular disease

Online Resource 11

First Oxford WebQ only

Association of depression risk with (a) UPF, (b) to (k) MUPs categories, and (l) to (w) specific MUPs indicated in Online Resource 6 (n=171,818; number of events=5,424). Models are adjusted as defined in Figure 1 and abbreviations are indicated in Figure 1

Online Resource 12

Further adjusted for diet quality

Association of depression risk with (a) UPF, (b) to (k) MUPs categories, and (l) to (w) specific MUPs indicated in Online Resource 6 (n=169,673; number of events=5,304). Models are adjusted as defined in Figure 1 and abbreviations are indicated in Figure 1

Online Resource 13

WHR and height instead of BMI

Association of depression risk with (a) UPF, (b) to (k) MUPs categories, and (l) to (w) specific MUPs indicated in Online Resource 6 (n=171,778; number of events=5,421). Models are adjusted as defined in Figure 1 and abbreviations are indicated in Figure 1. Further abbreviations: BMI, Body mass index; WHR, Waist-to-hip ratio

Online Resource 14

Energy intake removed

Association of depression risk with (a) UPF, (b) to (k) MUPs categories, and (l) to (w) specific MUPs indicated in Online Resource 6 (n=171,818; number of events=5,424). Models are adjusted as defined in Figure 1 and abbreviations are indicated in Figure 1

Online Resource 15

Adjustment for multiple testing

Association of MUP categories and specific MUPs with incident depression in the main and sensitivity analyses further adjusted for multiple testing using the Benjamini-Hochberg method for the MUP categories and specific MUPs separately. Results with an FDR-adjusted P<0.05 were considered as statistically significant and are only shown for associations which were significant in the main analyses without adjustment for multiple testing. The respective Online Resources are indicated (e.g., Landmark analysis for colouring agent is depicted in Online Resource 6d). Abbreviations: BMI, Body mass index; CVD, Cardiovascular disease; FDR, False discovery rate; MUP, Marker of ultra-processed food; ns, Non-significant; s, Significant, UPF, Ultra-processed food; WHR, Waist-to-hip ratio

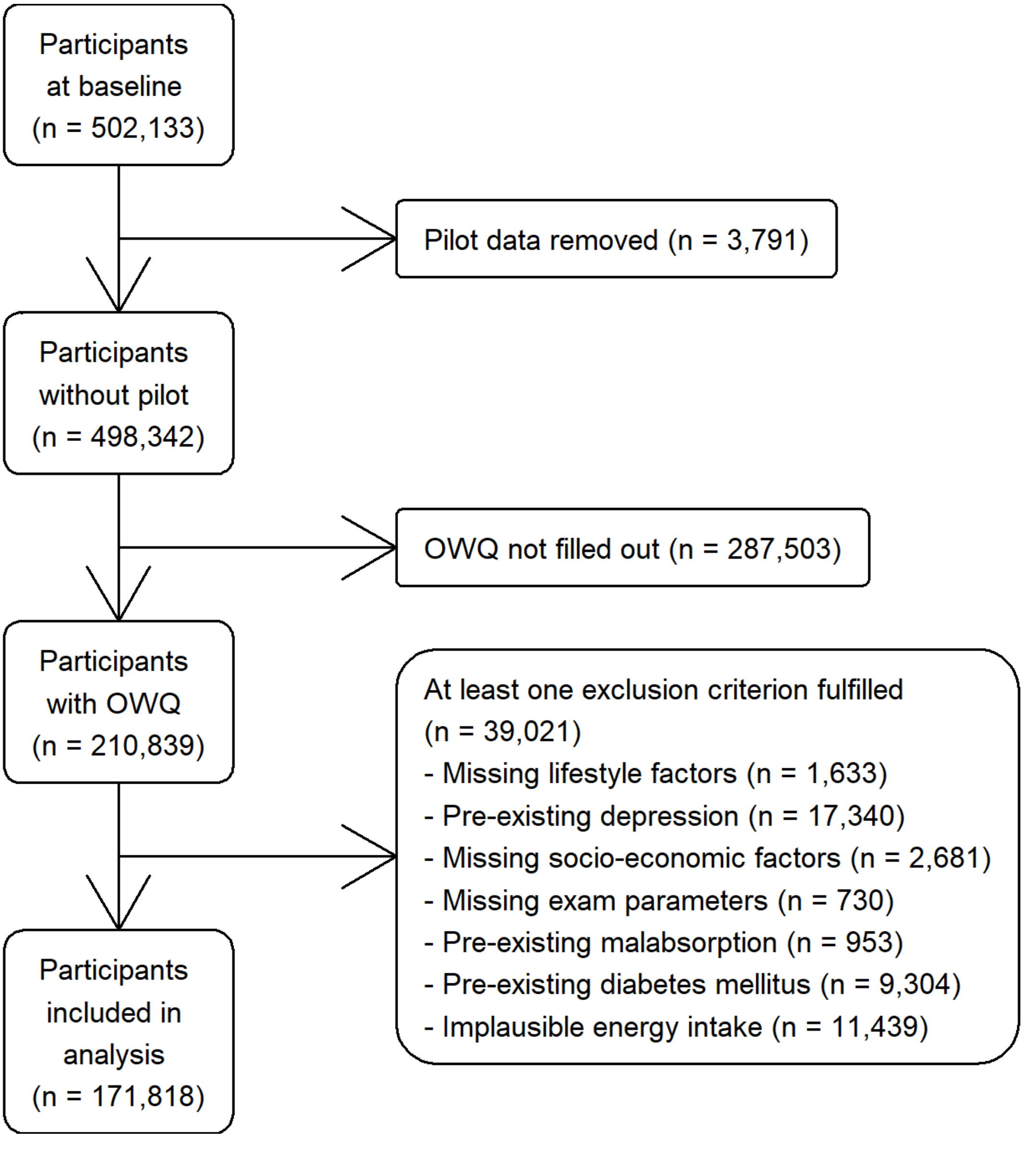

Online Resource 1 - Flowchart of participant selection

Online Resource 2. Specific MUPs and their individual compounds within the ten categories[a]

Categories	Specific MUPs	Individual compounds
**Cosmetic additives**		
**1. Flavour**	**1. Flavour***	Flavor<br>Flavour
2. Flavour enhancer	2. Glutamate	E620 – E625<br>Glutamate<br>Glutamic acids<br>MSG
	3. Glycine	E640<br>Glycinate<br>Glycine
	4. Guanylate	E626 – E629<br>Guanylate<br>Guanylic acids
	5. Inosinate	E630 – E633<br>Inosinate<br>Inosinic acids
	6. Maltol	E636 – E637<br>Maltol
	7. Ribonucleotide	5'-ribonucleotides<br>E634 – E635
	8. Zinc acetate	E650<br>Zinc (di)acetate
**3. Colouring agent**	**9. Colour***	Color<br>Color stabilizer<br>Colour<br>Colour stabiliser
	10. Dye	Dye
**4. Sweetener**	**11. Acesulfame**	Acesulfame K<br>E950
	12. Advantame	Advantame<br>E969
	13. Alitame	Alitame<br>E956
	**14. Aspartame**	Aspartame<br>E951
	15. Aspartame-Acesulfame	Aspartame-Acesulfame<br>E962
	16. Cyclamate	Cyclamate<br>Cyclamic acid<br>E952
	**17. Erythritol**	E968<br>Erythritol
	18. Glycyrrhizin	E958<br>Glycyrrhizic acid<br>Glycyrrhizin
	19. Isomalt	E953<br>Isomalt
	20. Lactitol	E966<br>Lactitol
	21. Maltitol	E965<br>Maltitol
	22. Mannitol	E421<br>Mannitol
	23. Neohesperidine	E959<br>Neohesperidine DC
	24. Neotame	E961<br>Neotame
	25. Polyglycitol	E964<br>Polyglycitol
	**26. Saccharin**	E954<br>Saccharin
	27. Sorbitol	E420<br>Sorbitol
	**28. Steviol**	E960<br>Steviol glycoside
	**29. Sucralose**	E955<br>Sucralose
	30. Tagatose	E963<br>Tagatose
	31. Thaumatin	E957<br>Thaumatin
	**32. Xylitol**	E967<br>Xylitol

5. Processing aid	33. Bulking agent	Anti-bulking<br>Bulking agent
	34. Caking agent	Anti-caking agent<br>Anticaking agent
	35. Carbonating agent	Carbonating agent
	36. Emulsifier	Emulsifier<br>Emulsifying salts
	37. Firming agent	Firming agent
	**38. Foaming agent**	Anti-foaming agent<br>De-foaming agent<br>Foaming agent
	39. Gelling agent	Gelling agent
	40. Glazing agent	Glazing agent
	41. Humectant	Humectant
	42. Sequestrant	Sequestrant
	**43. Thickener**	Thickener
**Non-culinary ingredients**		
6. Modified starch	44. Modified starch*	Modified starch
7. Varieties of sugar	45. Dextrose	Dextrose<br>Polydextrose#
	**46. Fructose**	Fructose<br>Fructose-glucose syrup#<br>Glucose-fructose syrup#<br>High-fructose corn syrup
	47. Inverted sugar	Inverted refiners syrup#<br>Inverted sugar syrup#<br>Invert sugar
	48. Juice concentrate	Juice concentrate
	49. Lactose	Lactose
	**50. Maltodextrin**	Maltodextrin
8. Modified oil	51. Hydrogenated oil*	Hydrogenated oil
	52. Interesterified oil	Interesterified oil
**9. Protein source**	*53. Casein*	Casein
	**54. Gluten**	Gluten
	55. Hydrolysed protein	Hydrolysed protein<br>Hydrolyzed protein
	56. Isolated protein	Isolated soy protein<br>Protein isolate#<br>Soy protein isolate
	57. Mechanically separated meat	Mechanically separated meat
	58. Whey protein	Dried whey#<br>Whey<br>Whey derivatives#<br>Whey permeate#<br>Whey powder#<br>Whey protein<br>Whey solids#
**10. Fibre**	**59. Fibre***	Fiber<br>Fibre<br>Fiber isolate#<br>Fibre isolate#<br>Insoluble fiber<br>Insoluble fibre<br>Soluble fiber<br>Soluble fibre

[a]Individual compounds are based on NOVA group 4 [1–3]. #Indicates individual compounds which are not literally mentioned in the three publications [1–3] but which are related to the ultra-processing compounds mentioned. A total of 39 out of 59 MUPs was detected in at least one commercial product. **Bold** indicates a significant (linear and/or non-linear P<0.05) association with incident depression in the main analysis. *Italics* indicates that the MUP was not present in any commercial product. MSG, Monosodium glutamate; MUP, Marker of ultra-processed food. *These MUPs are identical to the respective MUP category since either the search term is identical (i.e., flavour, modified starch, and fibre) or only one search term is present in at least one ingredient list (i.e., colour and hydrogenated oil) and, therefore, results are only shown for the MUP category

References

1. Monteiro CA, Cannon G, Moubarac J-C et al. (2017) The UN Decade of Nutrition, the NOVA food classification and the trouble with ultra-processing. Public Health Nutr 21:5–17. https://doi.org/10.1017/S1368980017000234
2. Monteiro CA, Cannon G, Levy RB et al. (2019) Ultra-processed foods: what they are and how to identify them. Public Health Nutr 22:936–941. https://doi.org/10.1017/S1368980018003762.
3. Martinez-Steele E, Khandpur N, Batis C et al. (2023) Best practices for applying the Nova food classification system. Nat Food 4:445–448. https://doi.org/10.1038/s43016-023-00779-w

Online Resource 3. Intake of specific MUPs at baseline in both the total cohort and the subgroups based on quintiles of UPF intake (%TFI)[a]

Parameters in %TFI	Total cohort (n = 171,818)	Quintiles of UPF intake (%TFI)				
		0.4 to 12.9 (n = 34,364)	12.9 to 16.5 (n = 34,363)	16.5 to 20.3 (n = 34,364)	20.3 to 25.9 (n = 34,363)	25.9 to 98.7 (n = 34,364)
	Mean (SD)	Mean (SD)	Mean (SD)	Mean (SD)	Mean (SD)	Mean (SD)
Glutamate	0.3 (0.5)	0.2 (0.4)	0.3 (0.4)	0.3 (0.5)	0.4 (0.6)	0.4 (0.7)
Guanylate	0.0 (0.1)	0.0 (0.0)	0.0 (0.1)	0.0 (0.1)	0.0 (0.1)	0.0 (0.1)
Inosinate	0.0 (0.1)	0.0 (0.0)	0.0 (0.1)	0.0 (0.1)	0.0 (0.1)	0.0 (0.1)
Ribonucleotide	0.3 (0.5)	0.2 (0.4)	0.3 (0.4)	0.3 (0.5)	0.4 (0.5)	0.4 (0.7)
Acesulfame	2.3 (4.2)	0.3 (0.6)	0.8 (1.2)	1.4 (1.8)	2.4 (2.8)	6.4 (7.0)
Aspartame	1.2 (2.0)	0.3 (0.5)	0.6 (0.7)	0.9 (1.0)	1.4 (1.4)	3.2 (3.3)
Erythritol	0.0 (0.1)	0.0 (0.1)	0.0 (0.1)	0.0 (0.1)	0.0 (0.1)	0.0 (0.1)
Isomalt	0.1 (0.2)	0.0 (0.1)	0.1 (0.2)	0.1 (0.2)	0.1 (0.2)	0.1 (0.3)
Maltitol	0.1 (0.1)	0.0 (0.1)	0.0 (0.1)	0.1 (0.1)	0.1 (0.1)	0.1 (0.1)
Saccharin	0.3 (0.7)	0.1 (0.2)	0.1 (0.3)	0.2 (0.4)	0.3 (0.6)	0.6 (1.3)
Sorbitol	0.1 (0.1)	0.1 (0.1)	0.1 (0.1)	0.1 (0.1)	0.1 (0.2)	0.1 (0.2)
Steviol	0.6 (1.7)	0.2 (0.3)	0.3 (0.5)	0.4 (0.7)	0.6 (1.1)	1.6 (3.5)
Sucralose	2.3 (4.0)	0.2 (0.6)	0.7 (1.2)	1.3 (1.9)	2.5 (2.8)	6.7 (6.2)
Xylitol	0.0 (0.0)	0.0 (0.0)	0.0 (0.0)	0.0 (0.0)	0.0 (0.0)	0.0 (0.0)
Bulking agent	0.1 (0.1)	0.1 (0.1)	0.1 (0.1)	0.1 (0.1)	0.1 (0.1)	0.1 (0.2)
Caking agent	0.5 (1.2)	0.2 (0.4)	0.3 (0.7)	0.5 (0.9)	0.6 (1.3)	0.9 (2.0)
Emulsifier	5.3 (3.0)	3.1 (1.5)	4.4 (1.8)	5.3 (2.2)	6.2 (2.7)	7.6 (3.8)
Firming agent	1.1 (1.0)	0.9 (0.8)	1.1 (0.9)	1.1 (1.0)	1.1 (1.0)	1.1 (1.1)
Foaming agent	0.0 (0.0)	0.0 (0.0)	0.0 (0.0)	0.0 (0.0)	0.0 (0.0)	0.0 (0.0)
Gelling agent	0.6 (0.5)	0.4 (0.3)	0.6 (0.4)	0.6 (0.5)	0.7 (0.5)	0.8 (0.6)
Glazing agent	0.2 (0.4)	0.1 (0.3)	0.2 (0.3)	0.2 (0.4)	0.2 (0.4)	0.2 (0.5)
Humectant	0.5 (0.7)	0.3 (0.5)	0.5 (0.6)	0.5 (0.7)	0.6 (0.8)	0.7 (0.9)
Sequestrant	0.0 (0.0)	0.0 (0.0)	0.0 (0.0)	0.0 (0.0)	0.0 (0.0)	0.0 (0.1)
Thickener	0.9 (1.2)	0.4 (0.4)	0.6 (0.7)	0.8 (0.9)	1.2 (1.1)	1.7 (1.8)
Dextrose	2.6 (1.8)	1.5 (0.9)	2.1 (1.1)	2.5 (1.3)	3.0 (1.7)	3.9 (2.4)
Fructose	0.8 (0.7)	0.4 (0.3)	0.6 (0.4)	0.7 (0.5)	0.9 (0.6)	1.3 (1.0)
Inverted sugar	0.5 (0.6)	0.3 (0.4)	0.5 (0.5)	0.6 (0.5)	0.6 (0.6)	0.7 (0.7)
Juice concentrate	0.3 (0.4)	0.2 (0.3)	0.3 (0.3)	0.3 (0.4)	0.4 (0.4)	0.5 (0.6)
Lactose	0.4 (0.6)	0.2 (0.3)	0.3 (0.4)	0.4 (0.5)	0.5 (0.6)	0.7 (1.0)
Maltodextrin	0.9 (0.8)	0.7 (0.5)	0.8 (0.6)	0.9 (0.7)	1.0 (0.8)	1.2 (1.1)
Gluten	1.3 (1.2)	0.9 (0.7)	1.2 (0.9)	1.3 (1.0)	1.5 (1.2)	1.7 (1.6)
Hydrolysed protein	0.1 (0.1)	0.1 (0.1)	0.1 (0.1)	0.1 (0.1)	0.1 (0.1)	0.1 (0.2)
Isolated protein	0.0 (0.1)	0.0 (0.0)	0.0 (0.1)	0.0 (0.1)	0.0 (0.1)	0.0 (0.1)
Whey protein	1.4 (1.3)	0.7 (0.7)	1.1 (0.9)	1.3 (1.1)	1.6 (1.3)	2.0 (1.9)

[a]%TFI, Percentage total food intake; MUP, Marker of ultra-processed food; SD, Standard deviation; UPF, Ultra-processed food

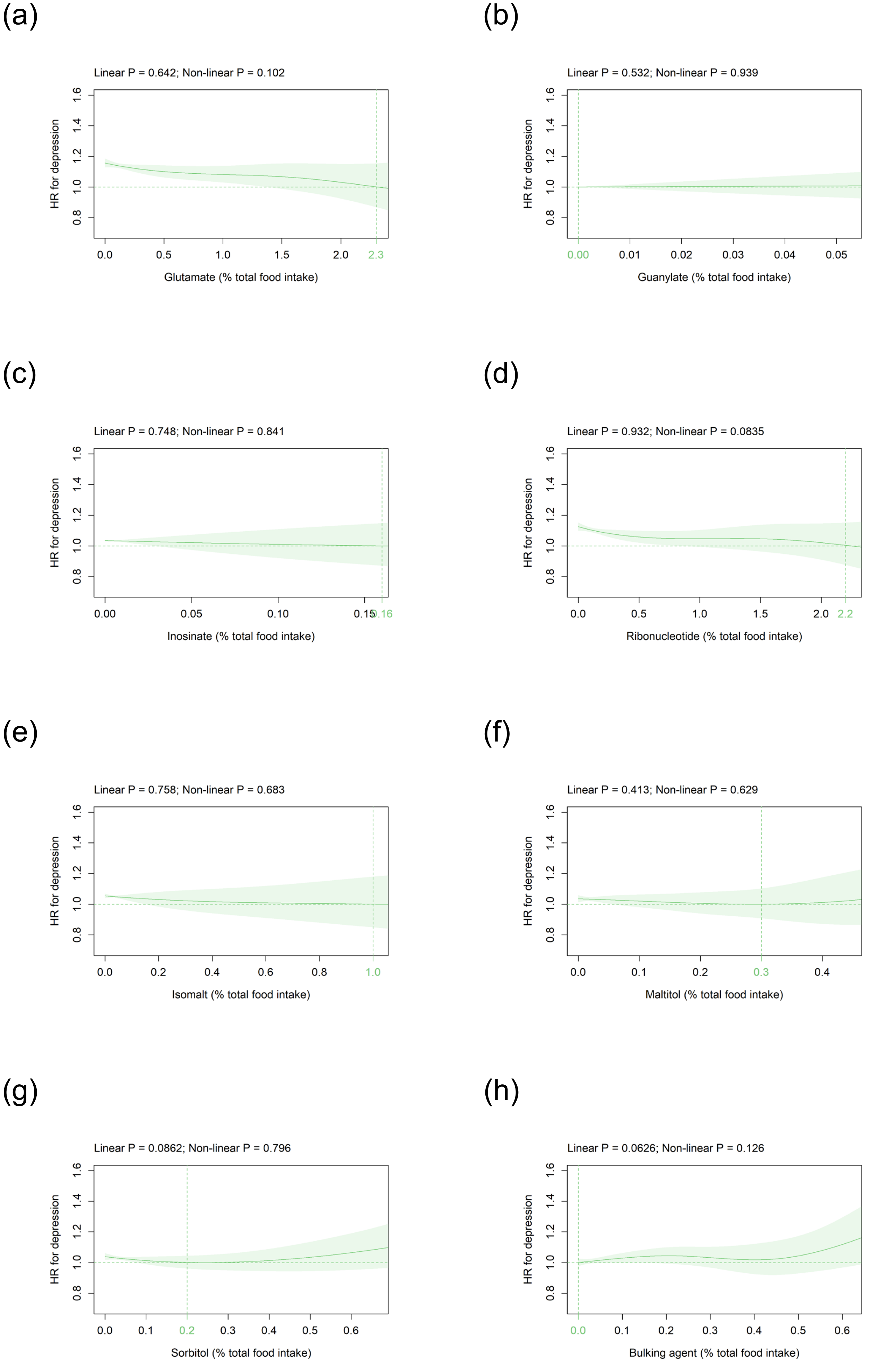

Online Resource 4 - Non-significant associations of specific MUPs

(i)

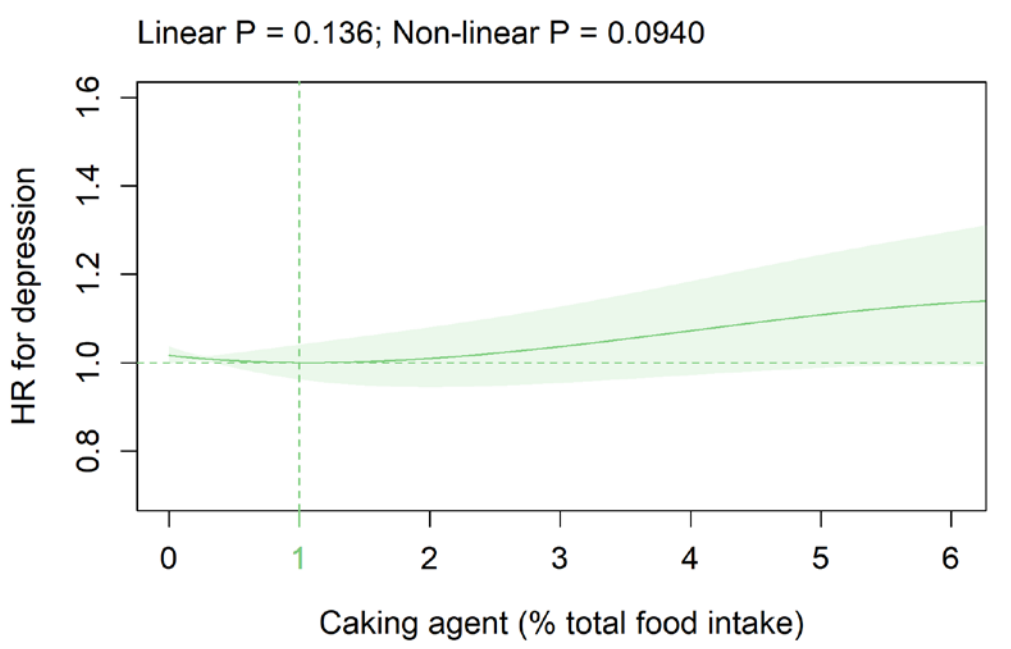

(j)

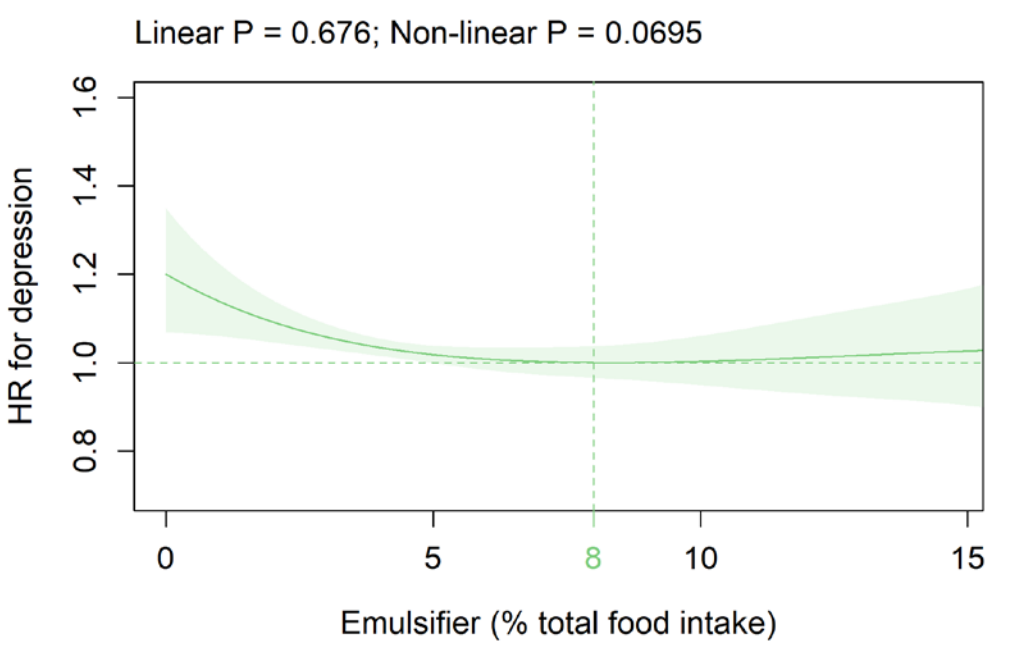

(k)

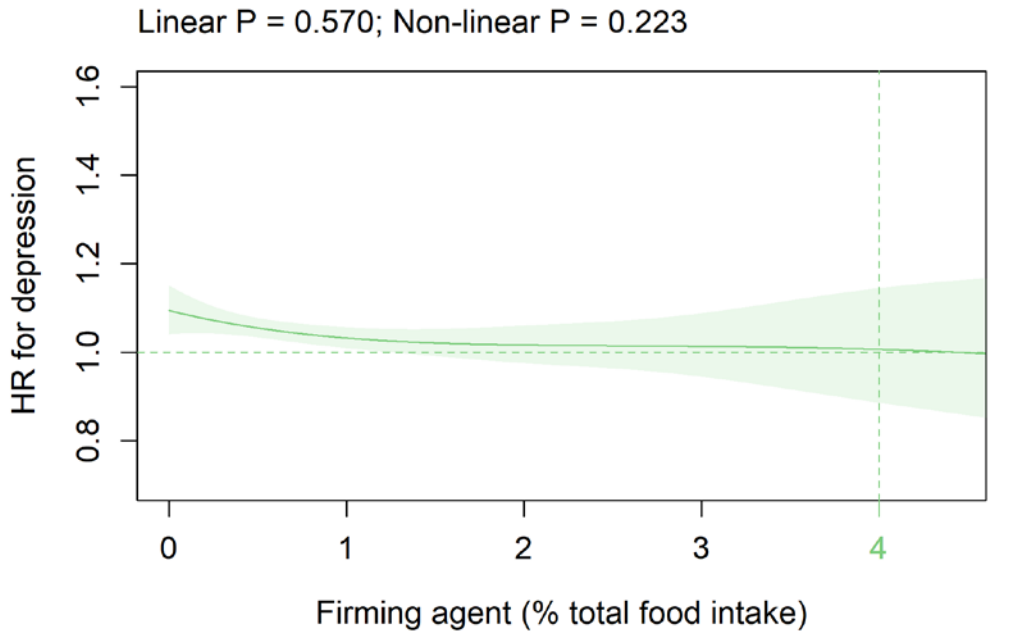

(l)

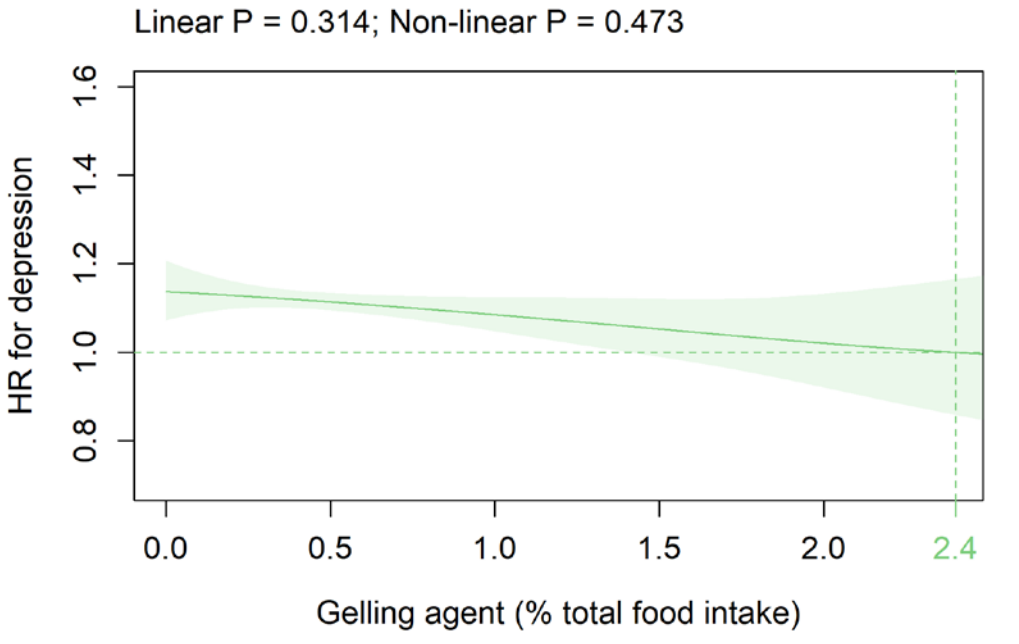

(m)

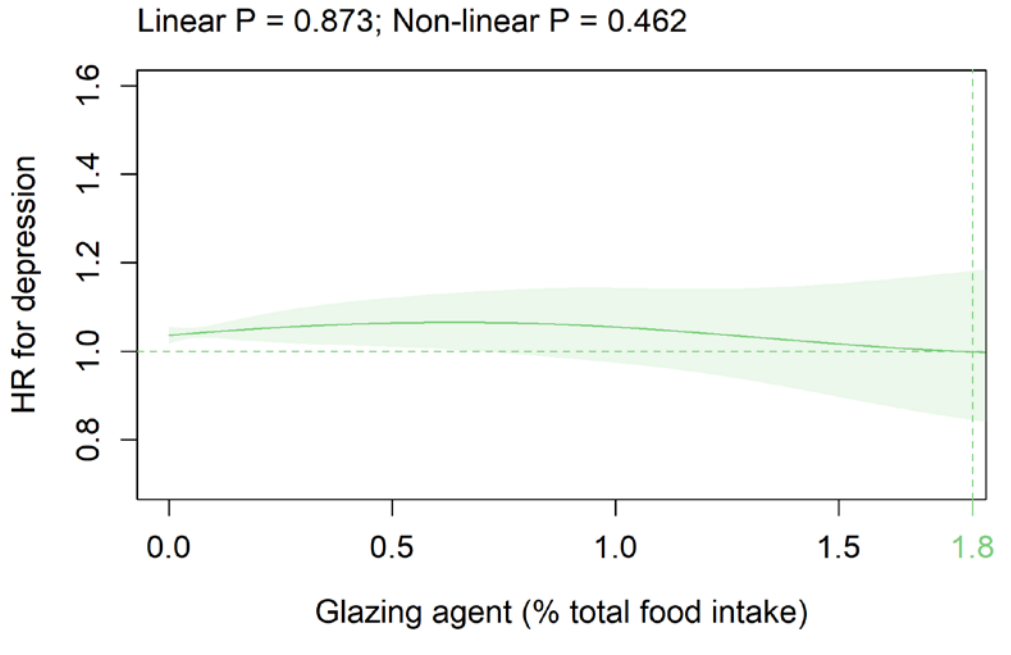

(n)

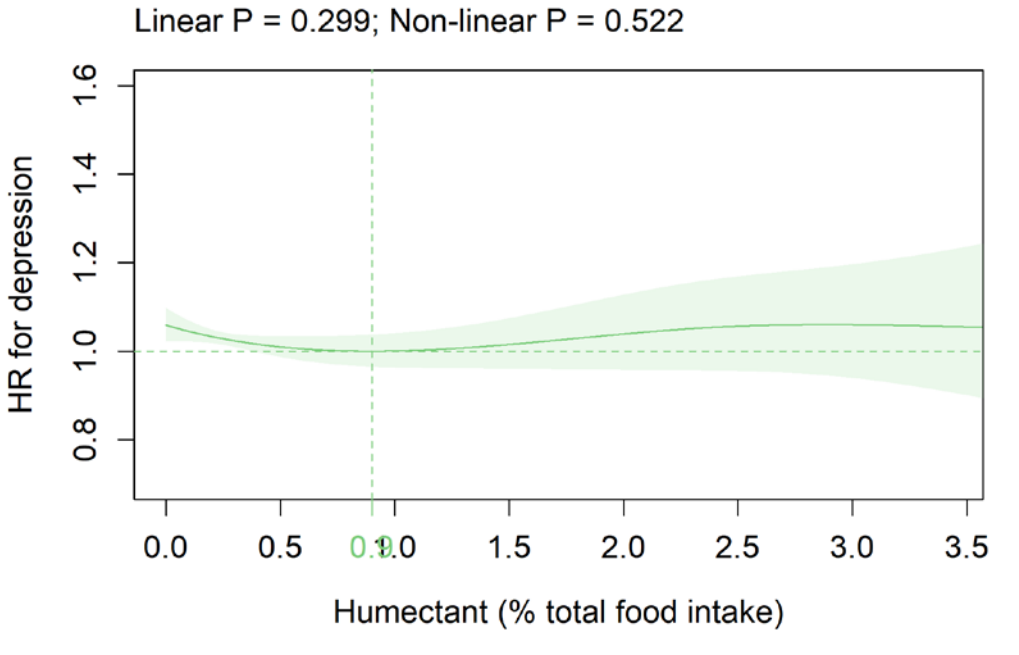

(o)

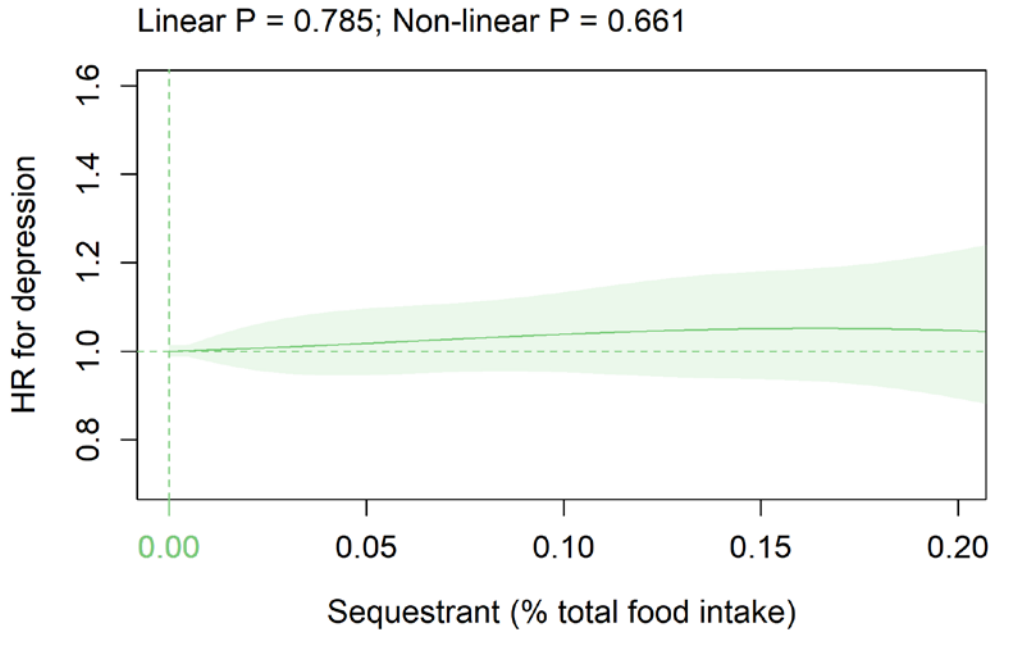

(p)

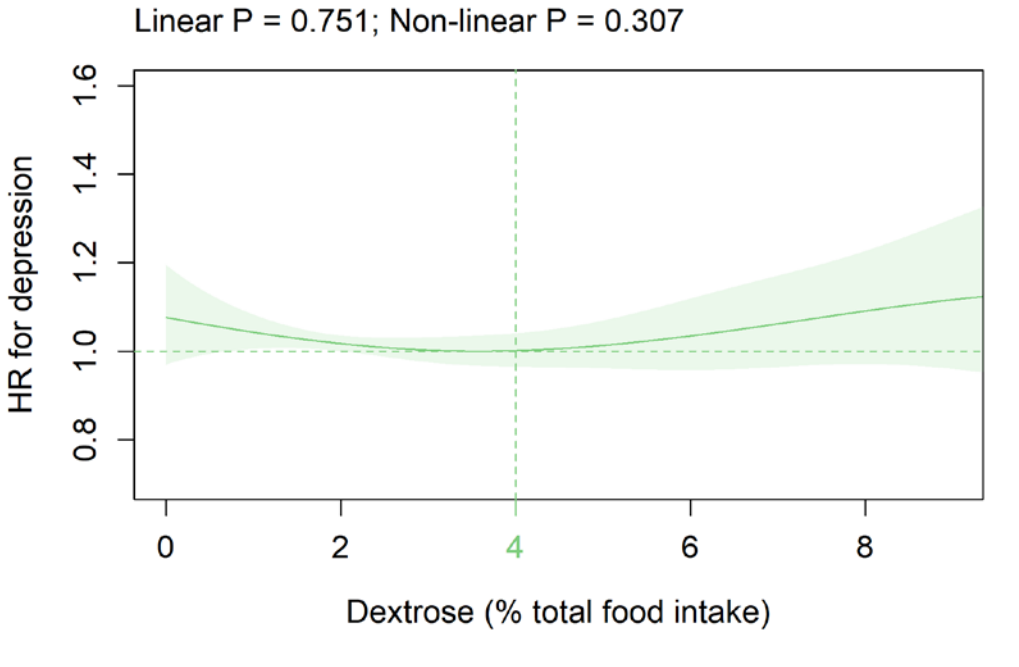

Online Resource 4 - Non-significant associations of specific MUPs

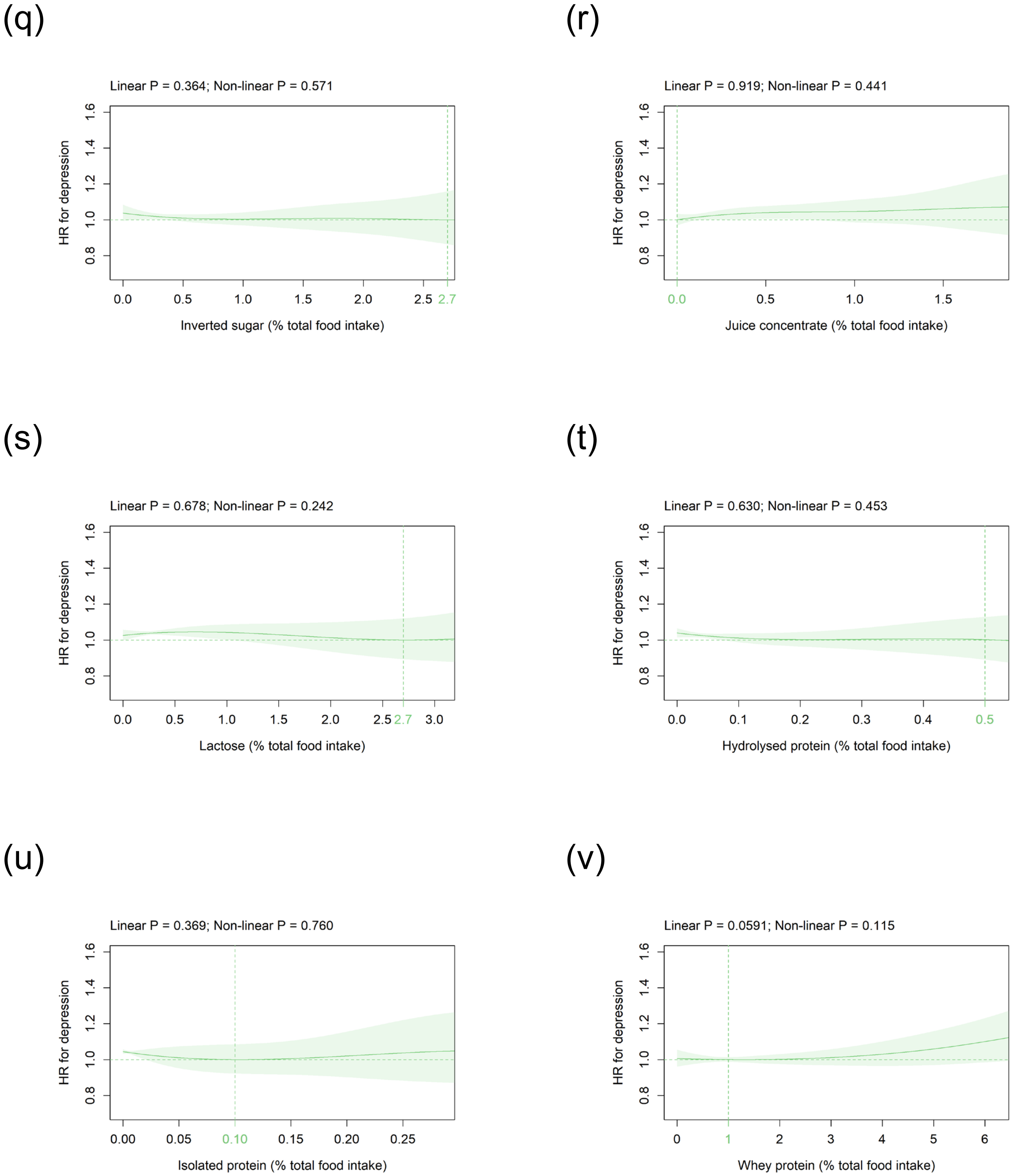

Online Resource 4 - Non-significant associations of specific MUPs

Online Resource 5. Association of UPF, MUP categories, and specific MUPs with incident depression in all sensitivity analyses. Results are only shown for associations which were significant in the main analyses[a]

		Landmark analysis	Unintentional weight loss removed	Atypical diet excluded	More than one Oxford WebQ	History of CVD and cancer excluded	First Oxford WebQ only	Further adjusted for diet quality	WHR and height instead of BMI	Energy intake removed
	Online Resource	6	7	8	9	10	11	12	13	14
UPF	a	s	s	s	s	s	s	s	s	s
Category										
- Flavour	b	s	s	s	s	s	s	s	s	s
- Colouring agent	d	s	s	s	s	s	s	s	s	s
- Sweetener	e	s	s	s	s	s	s	s	s	s
- Protein source	j	s	ns	s	ns	s	ns	s	s	ns
- Fibre	k	s	ns	ns	s	s	ns	ns	s	ns
MUP										
- Acesulfame	l	s	s	s	s	s	s	s	s	s
- Aspartame	m	s	s	s	s	s	s	s	s	s
- Erythritol	n	s	s	s	s	s	s	s	s	s
- Saccharin	o	s	ns	s	s	s	s	s	s	s
- Steviol	p	s	s	s	ns	s	s	s	s	s
- Sucralose	q	s	s	s	s	s	s	s	s	s
- Xylitol	r	s	s	s	s	s	s	s	s	s
- Foaming agent	s	s	ns	s	ns	s	ns	s	s	s
- Thickener	t	s	s	s	ns	s	ns	s	s	s
- Fructose	u	s	s	s	s	s	s	s	s	s
- Maltodextrin	v	ns	s	s	ns	s	s	s	s	s
- Gluten	w	s	ns	s	s	s	s	s	s	s

[a]The respective Online Resources are indicated (e.g., Landmark analysis for colouring agent is depicted in Online Resource 6d). Abbreviations: BMI, Body mass index; CVD, Cardiovascular disease; MUP, Marker of ultra-processed food; ns, Non-significant; s, Significant, UPF, Ultra-processed food; WHR, Waist-to-hip ratio

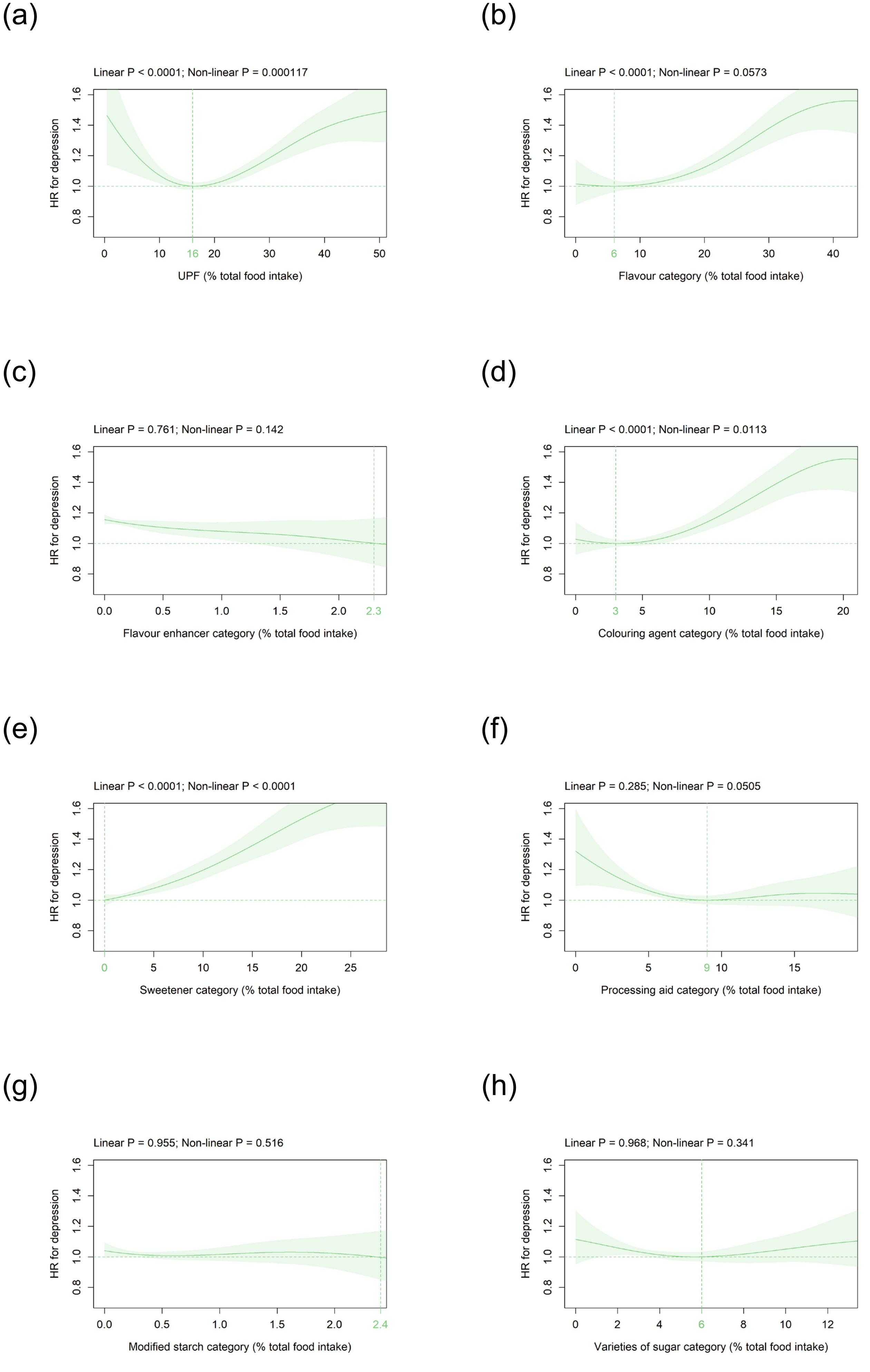

Online Resource 6 - Landmark analysis

(i)

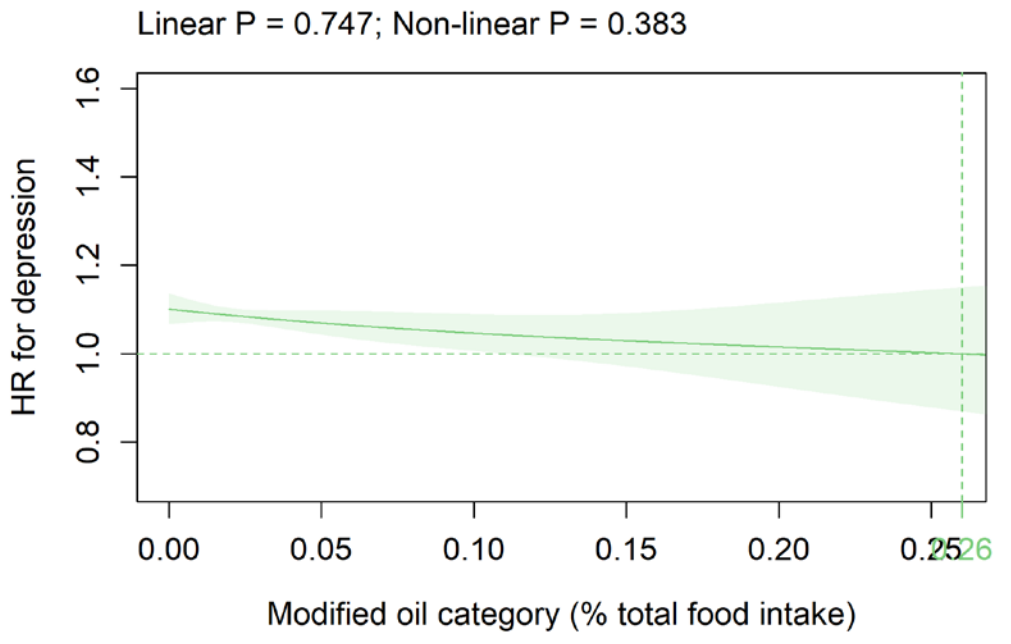
Linear P = 0.747; Non-linear P = 0.383
HR for depression
0.8
1.0
1.2
1.4
1.6
0.00
0.05
0.10
0.15
0.20
0.25
0.26
Modified oil category (% total food intake)

(j)

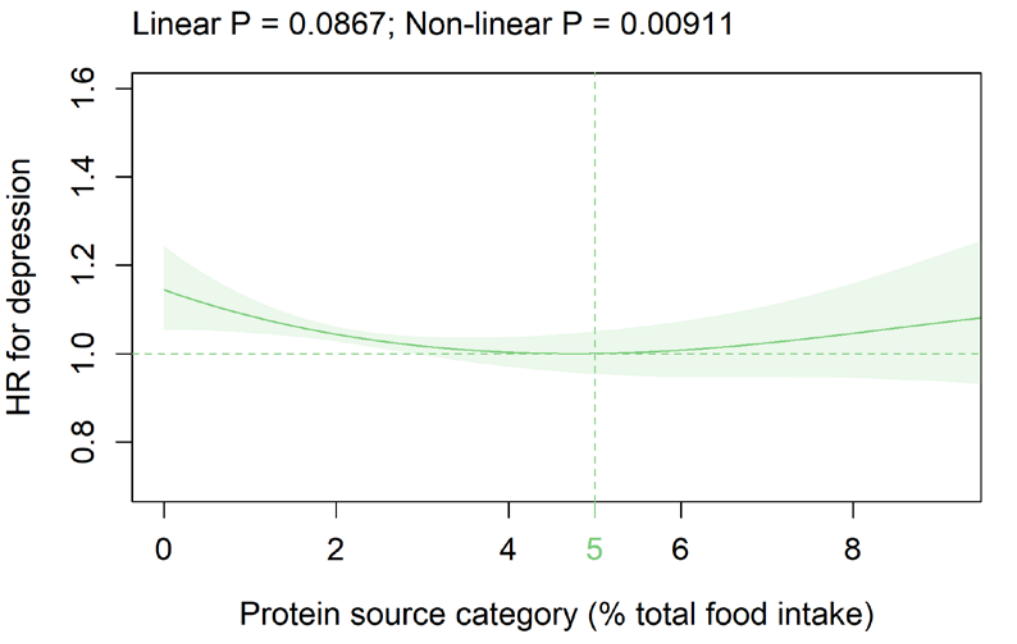
Linear P = 0.0867; Non-linear P = 0.00911
HR for depression
0.8
1.0
1.2
1.4
1.6
0
2
4
5
6
8
Protein source category (% total food intake)

(k)

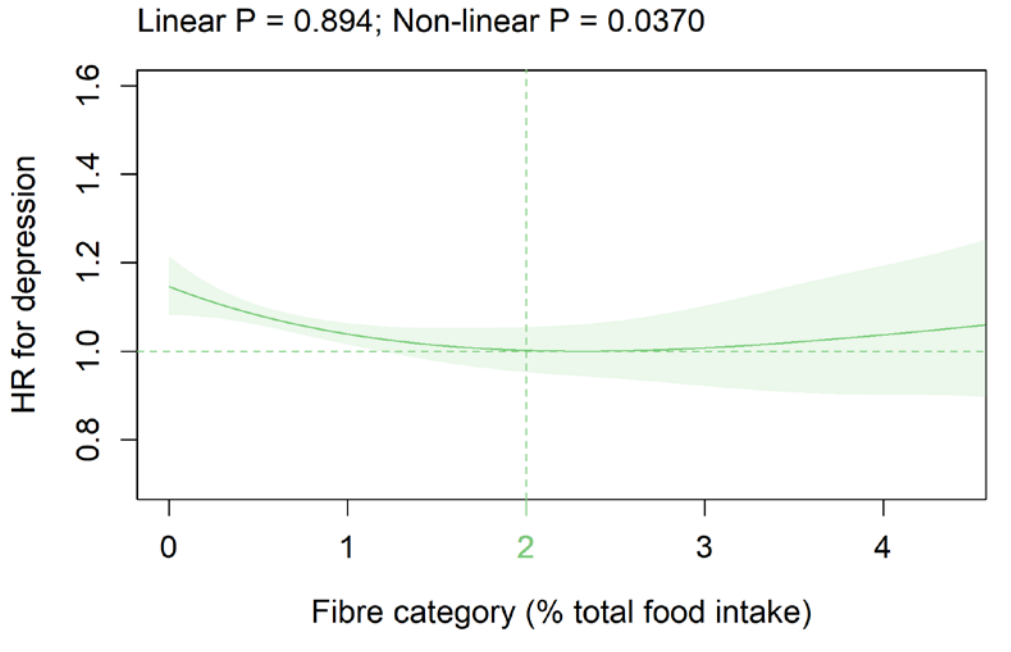
Linear P = 0.894; Non-linear P = 0.0370
HR for depression
0.8
1.0
1.2
1.4
1.6
0
1
2
3
4
Fibre category (% total food intake)

(l)

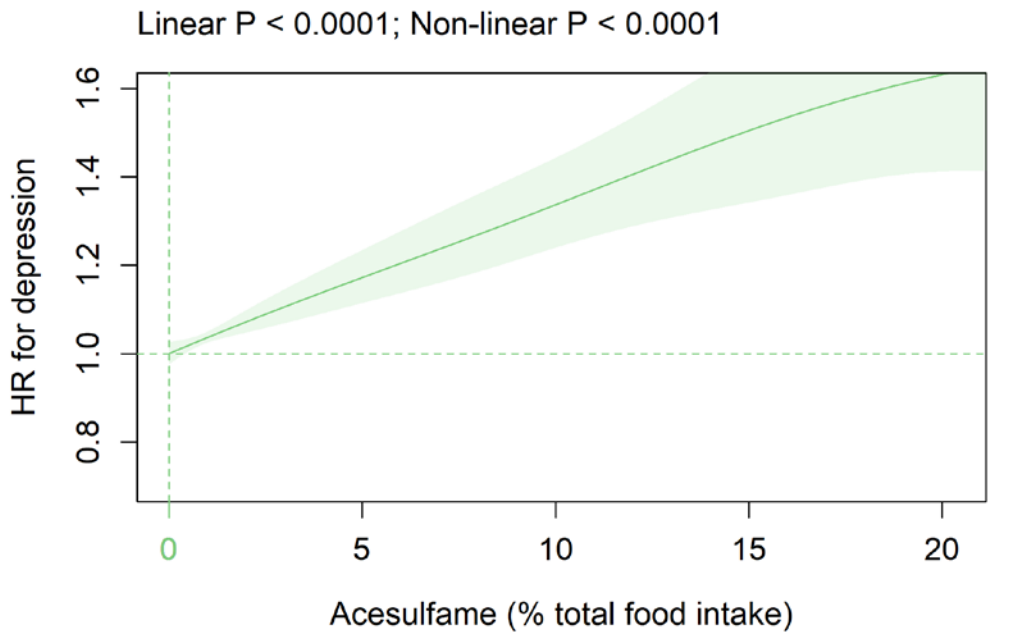

(m)

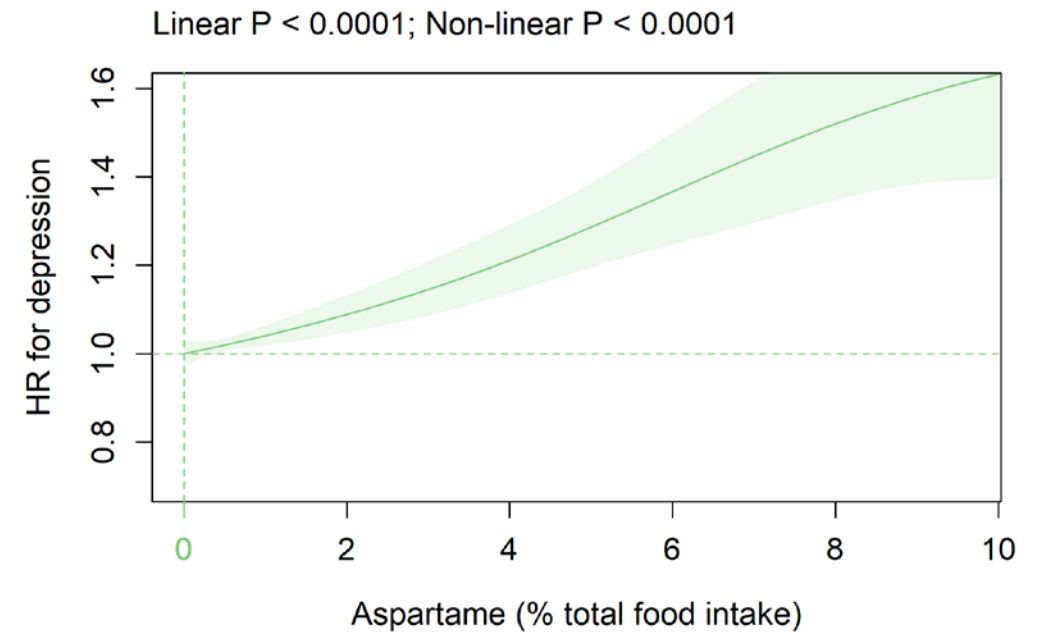

(n)

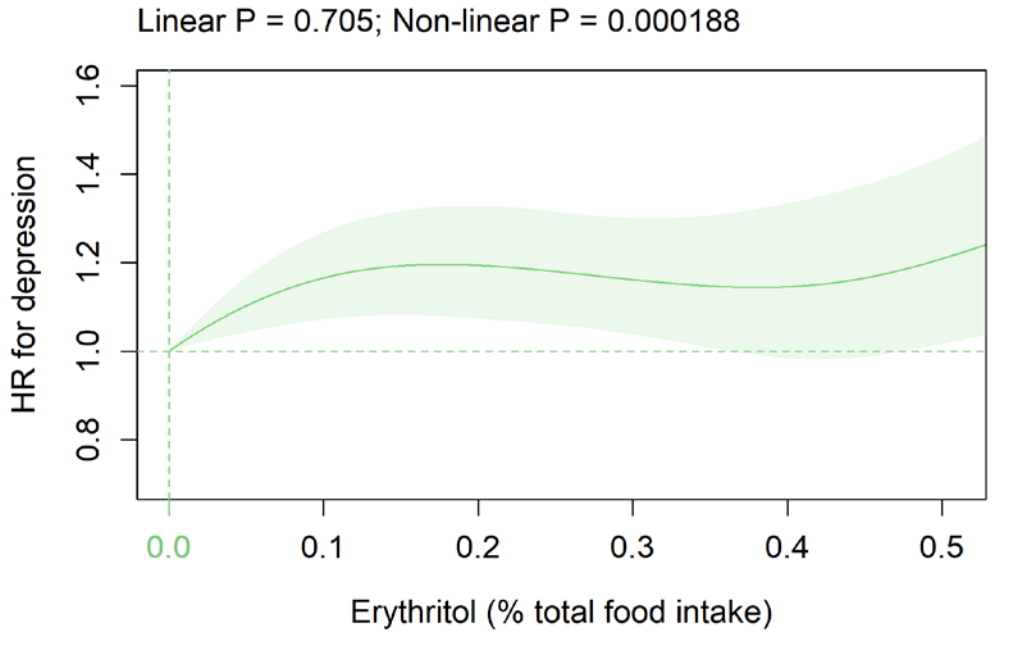

(o)

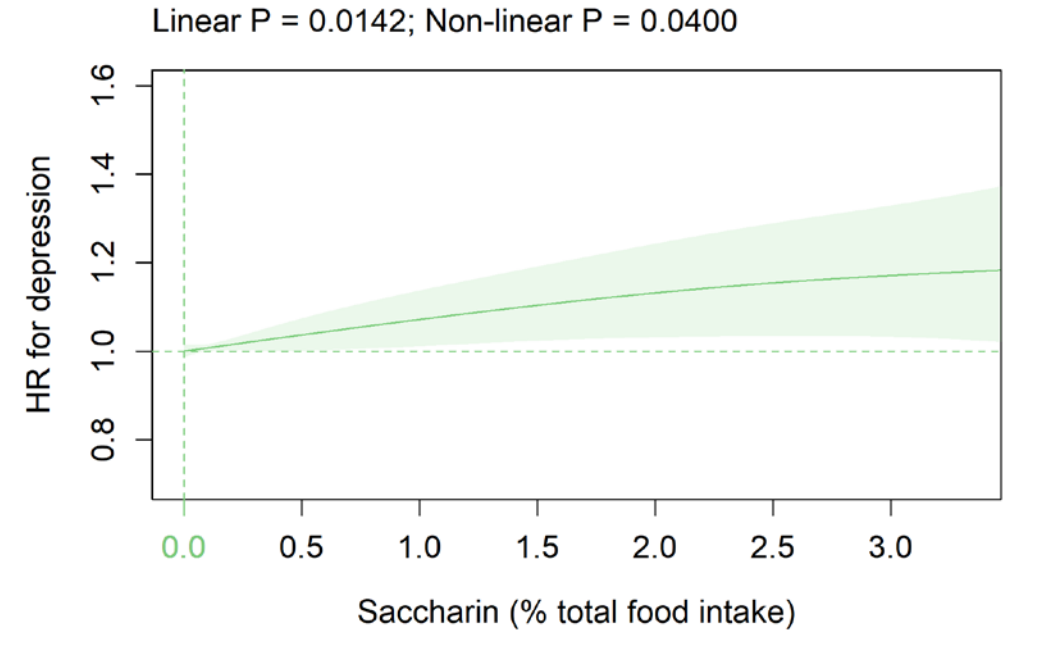

(p)

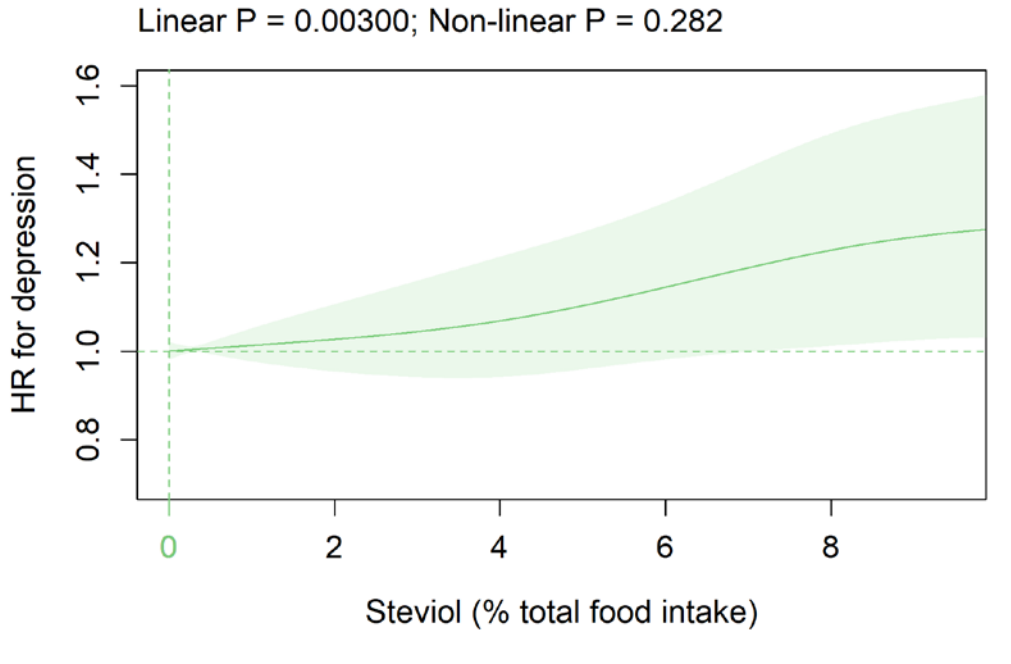

(q)

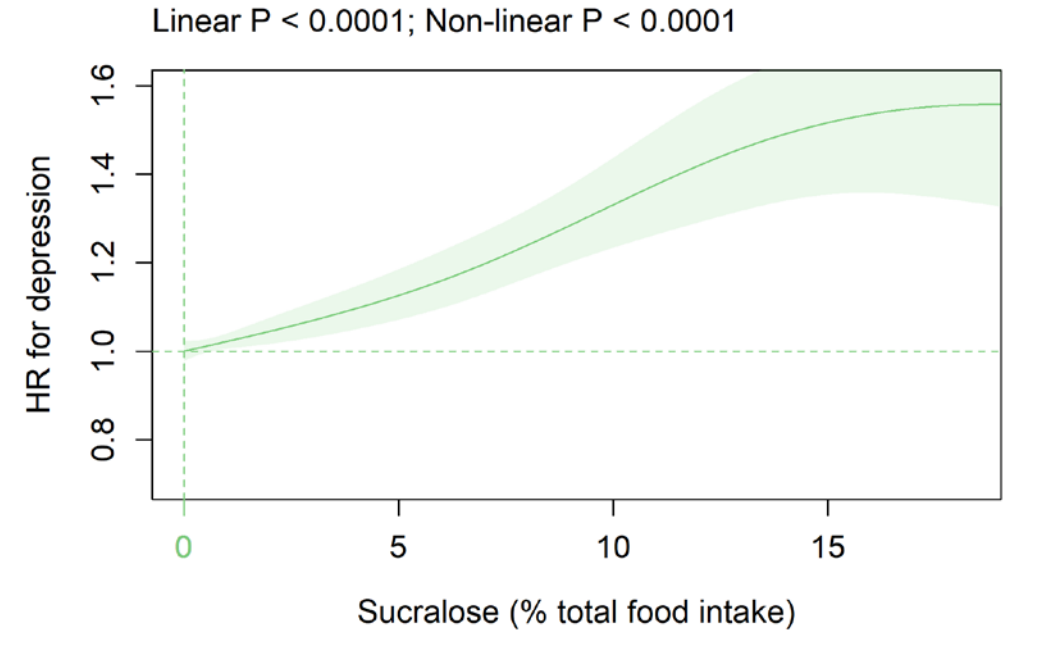

(r)

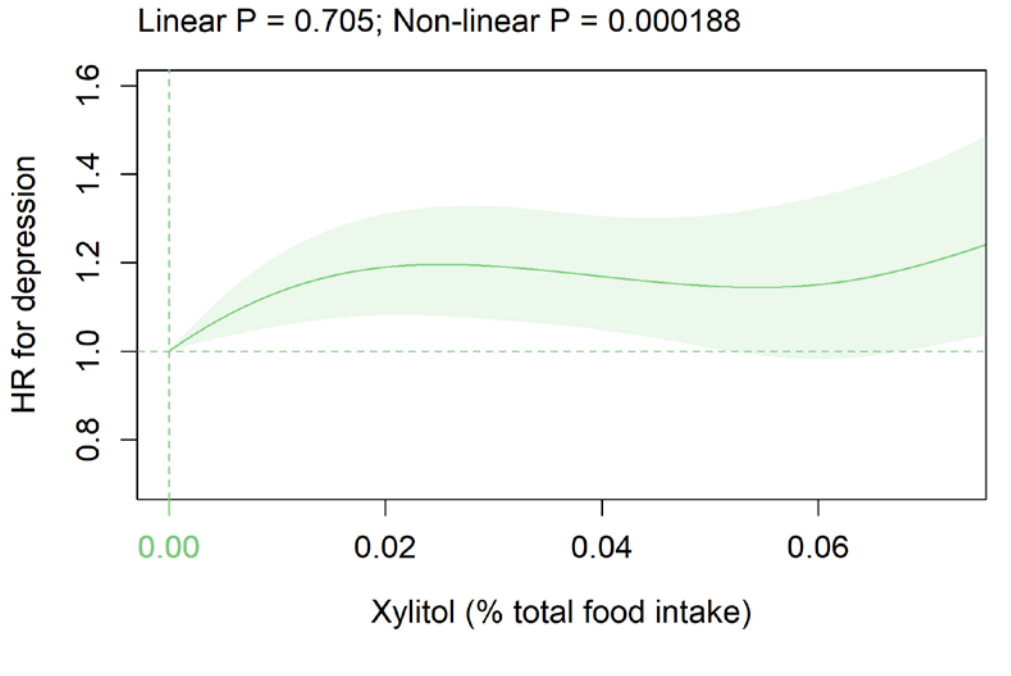

(s)

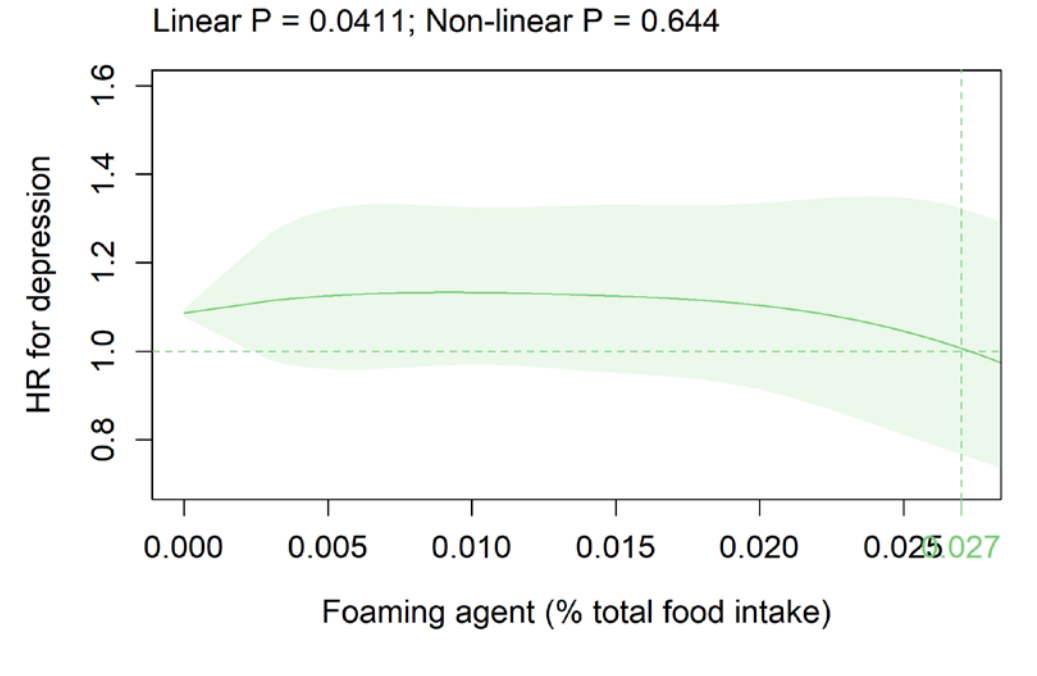

Online Resource 6 - Landmark analysis

(t)

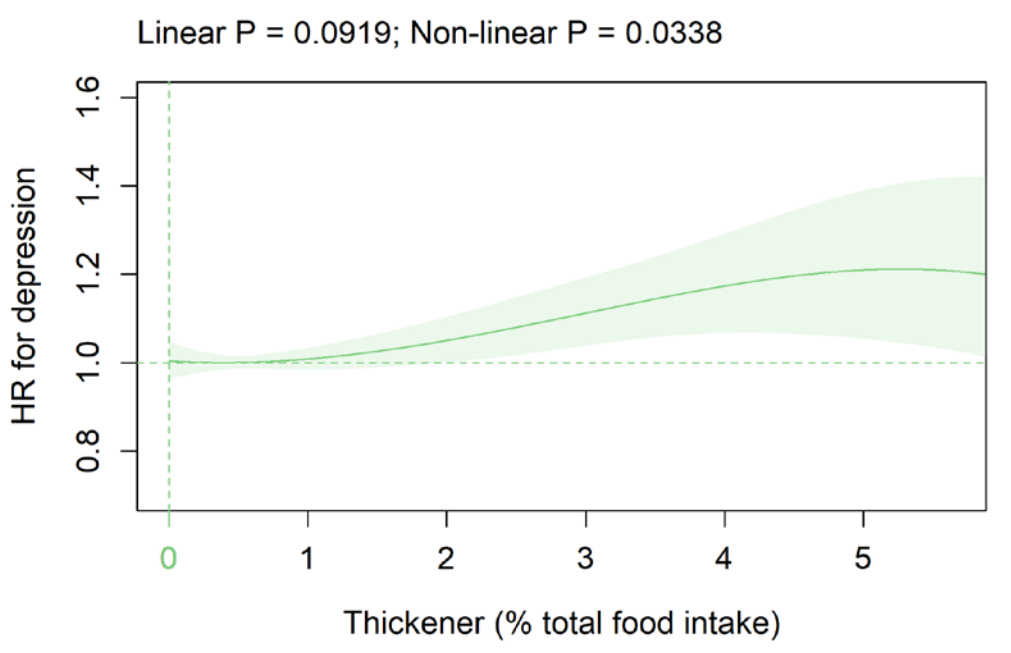

(u)

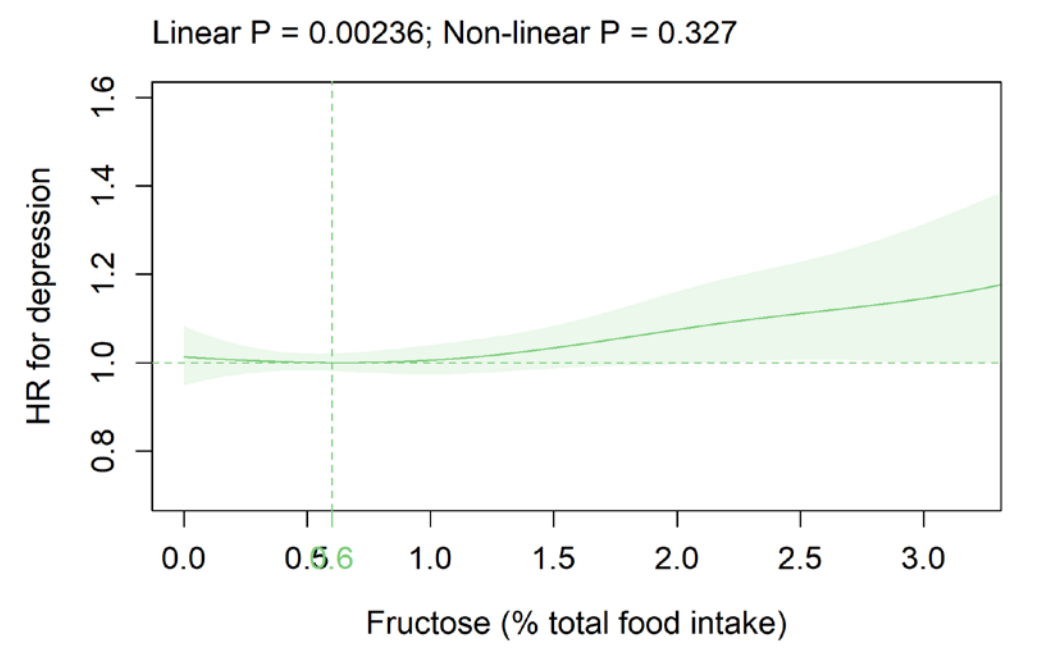

(v)

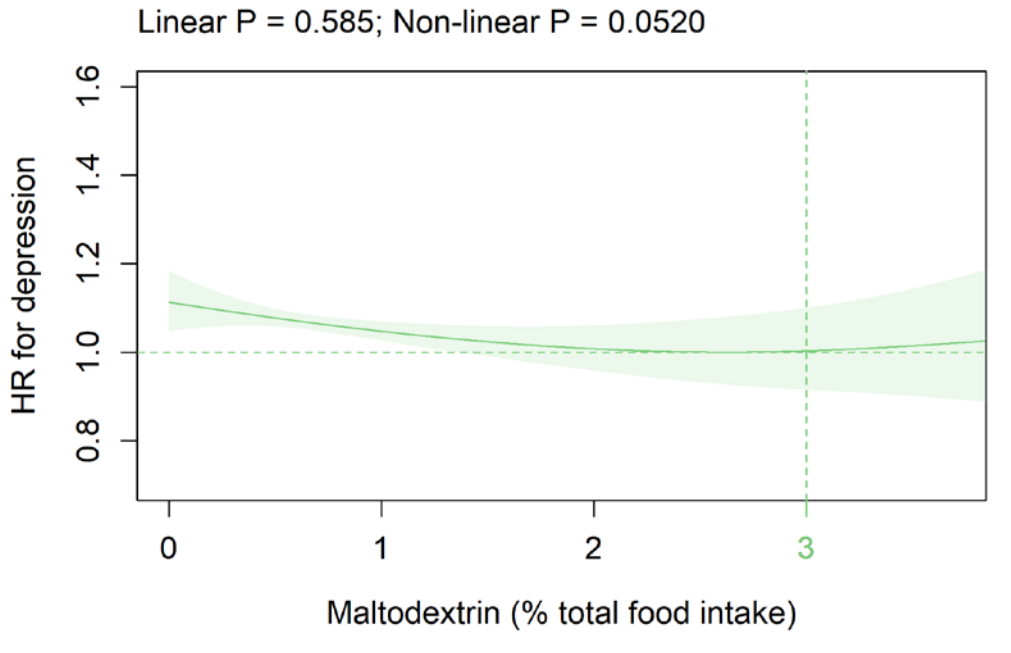

(w)

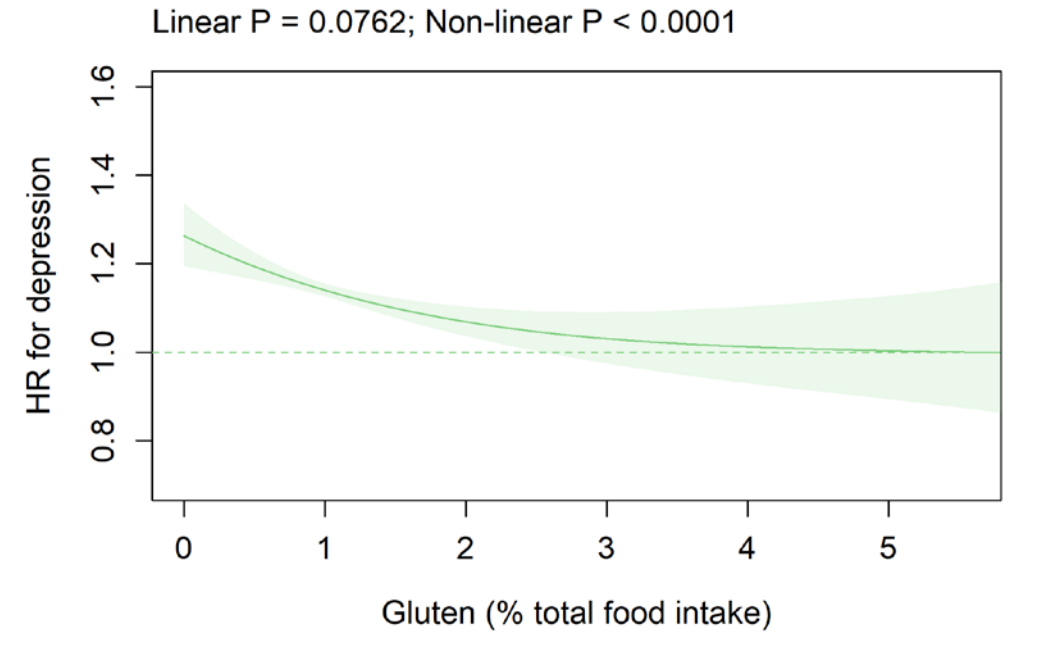

Online Resource 6 - Landmark analysis

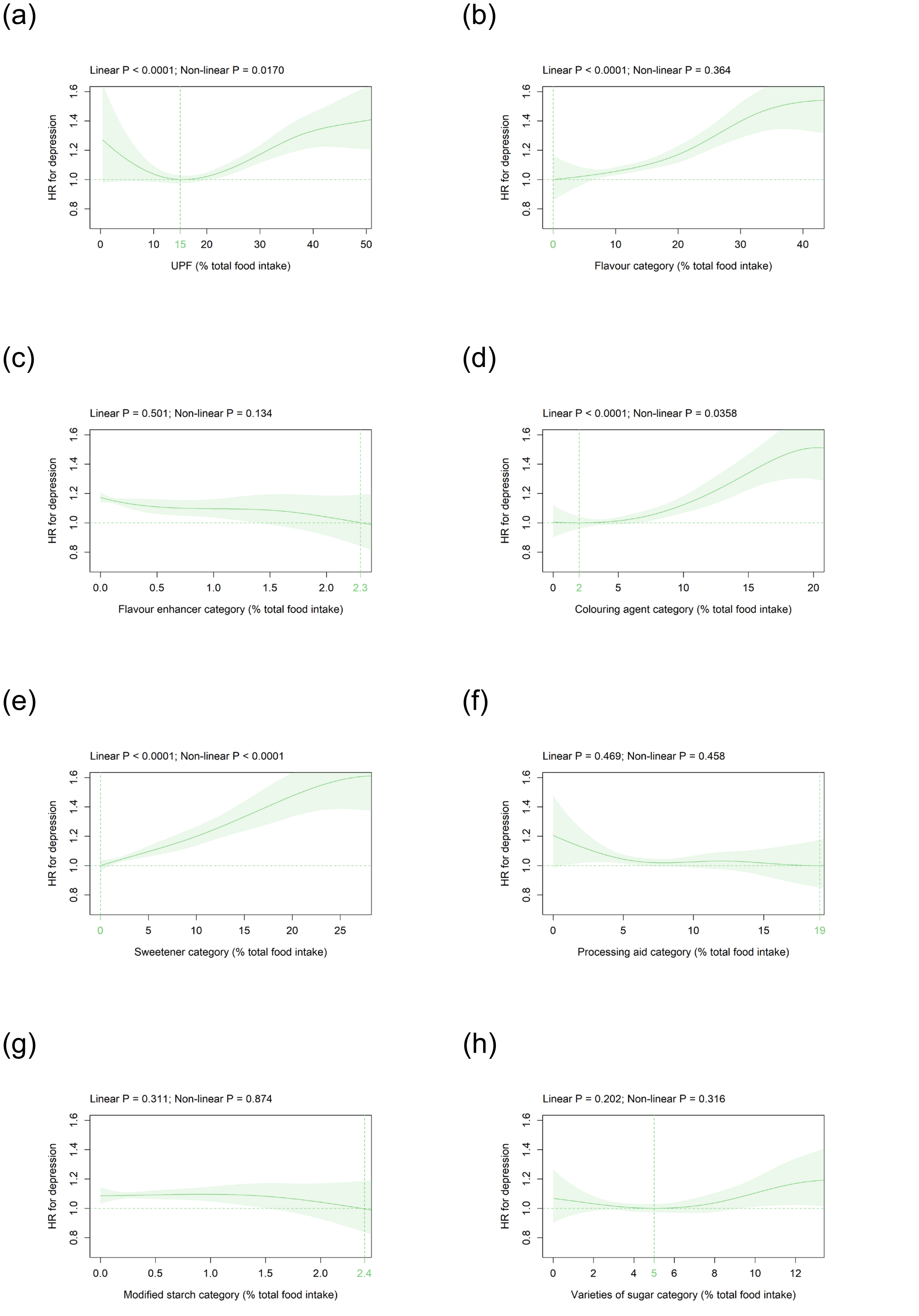

Online Resource 7 - Unintentional weight loss removed

(i)

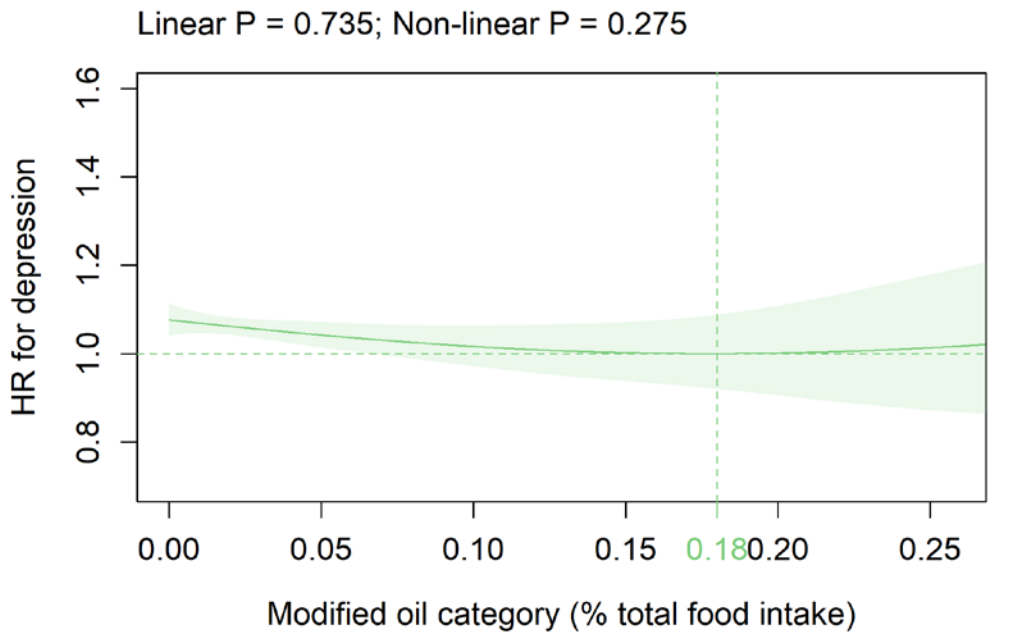

(j)

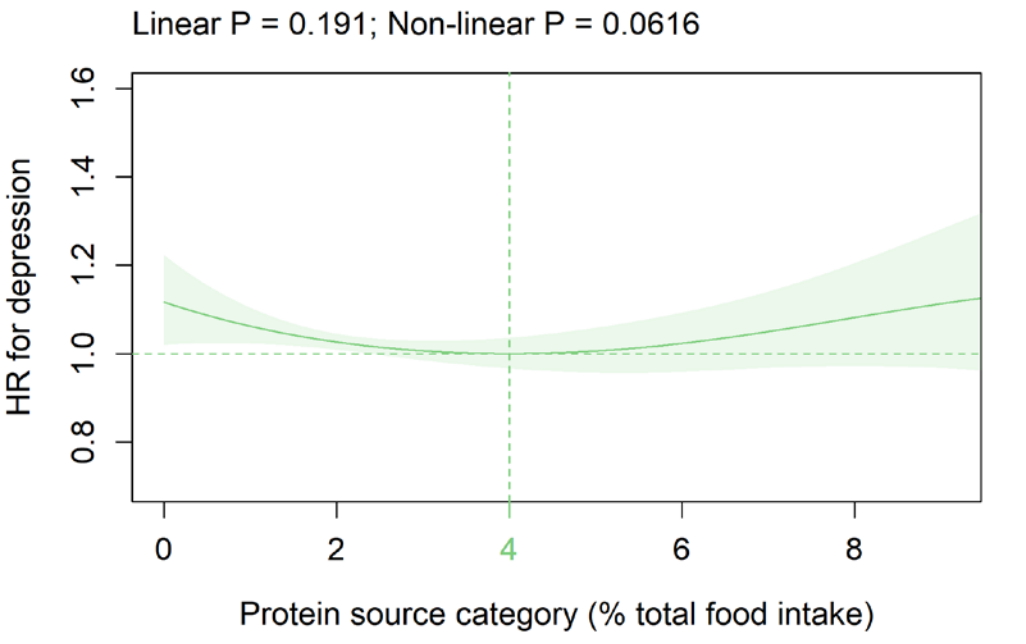

(k)

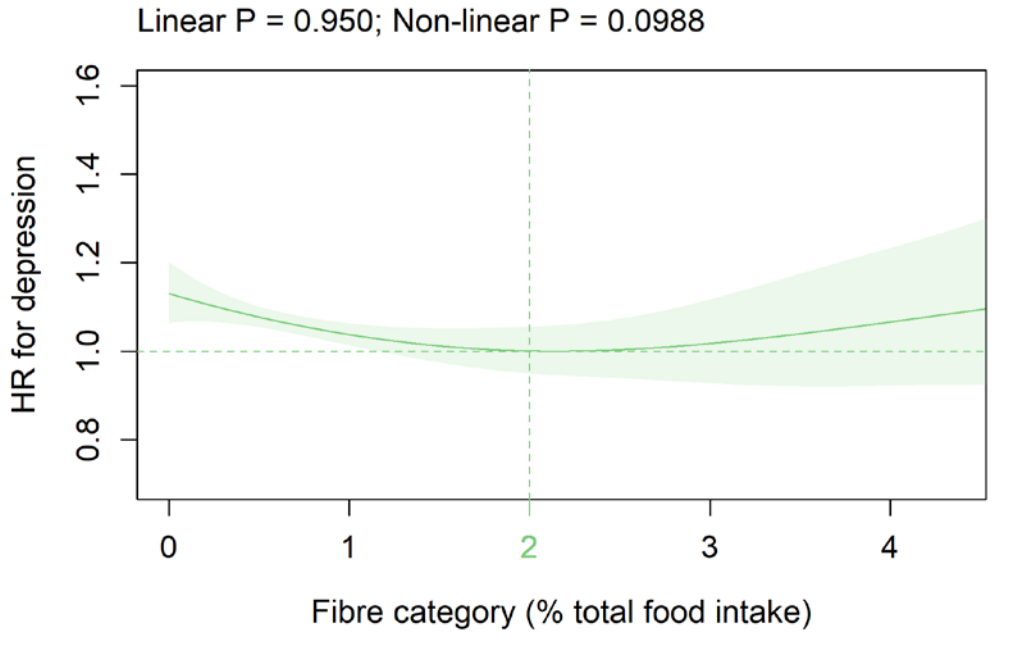

Online Resource 7 - Unintentional weight loss removed

(l)

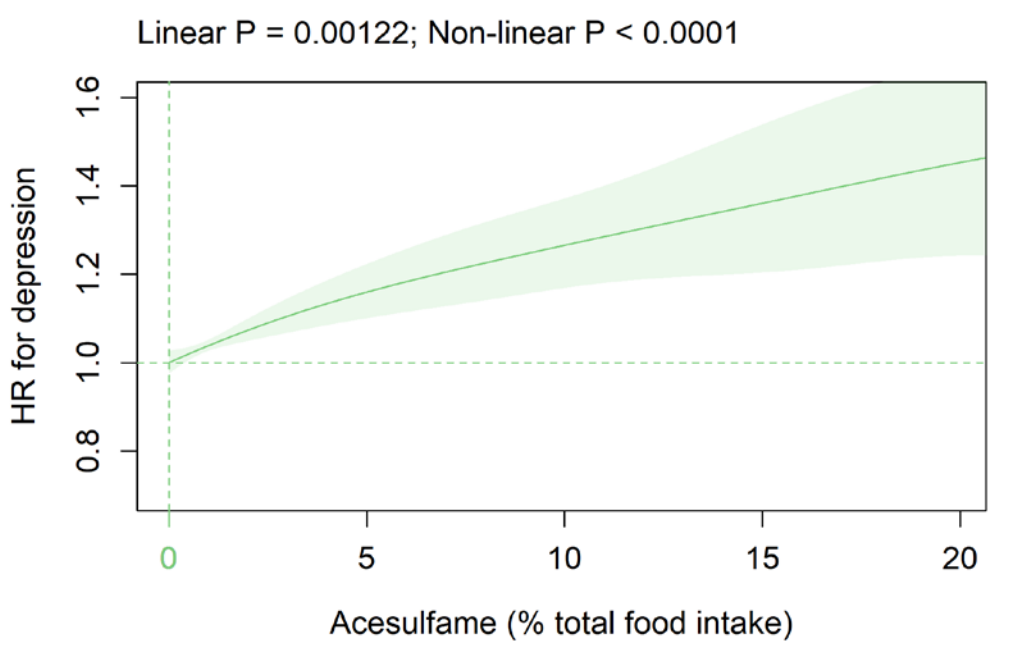

(m)

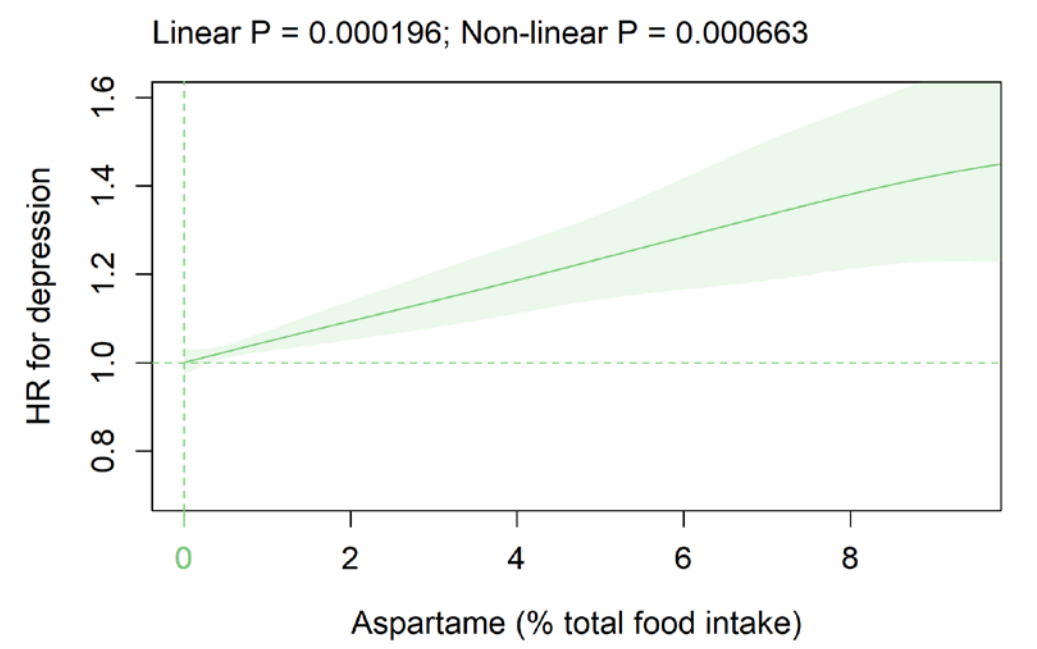

(n)

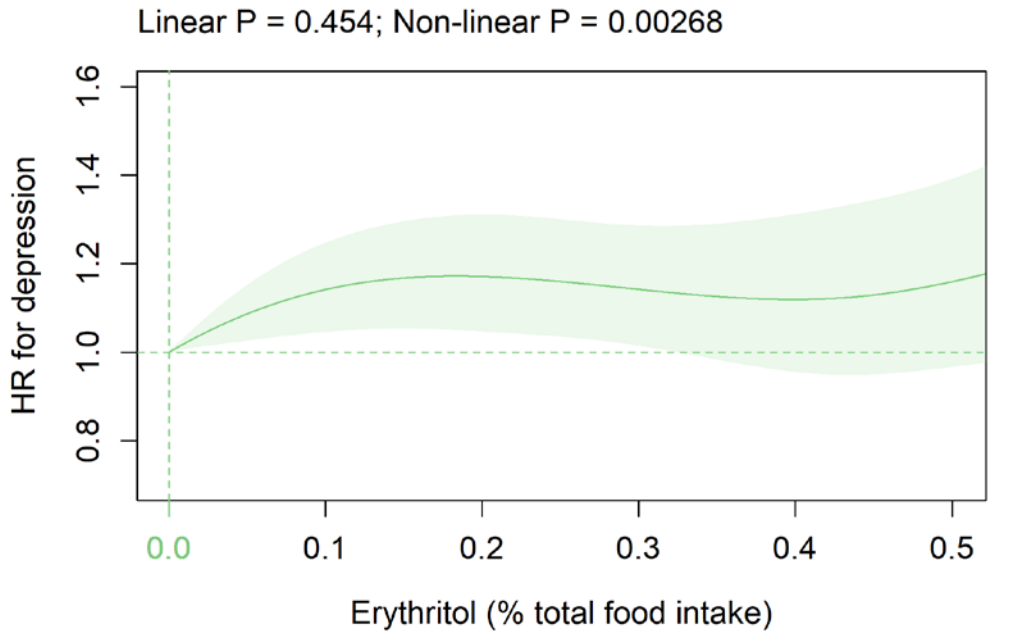

(o)

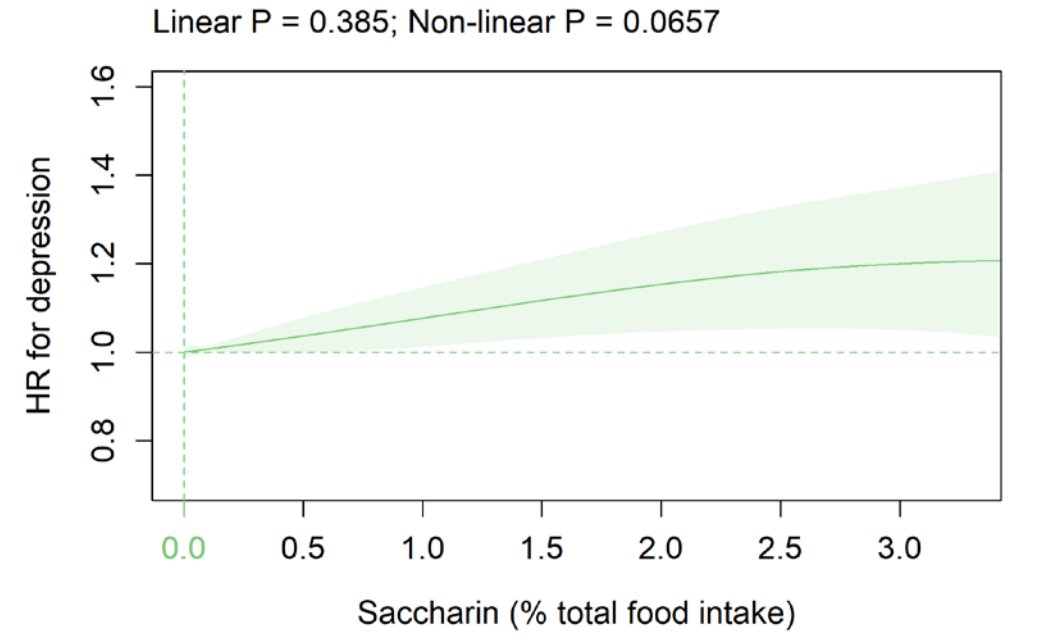

(p)

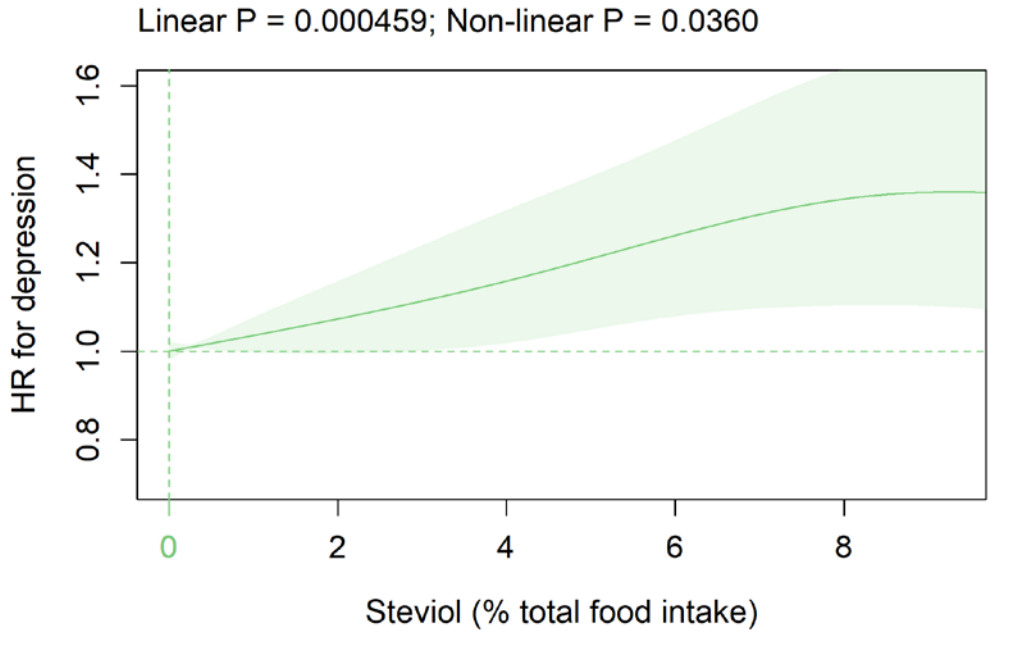

(q)

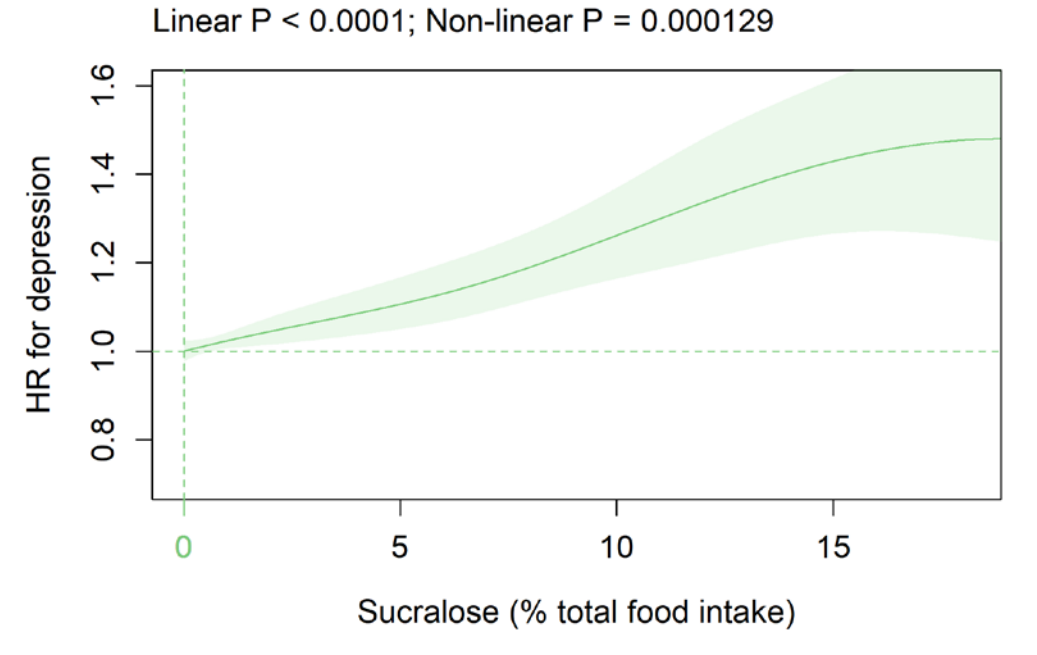

(r)

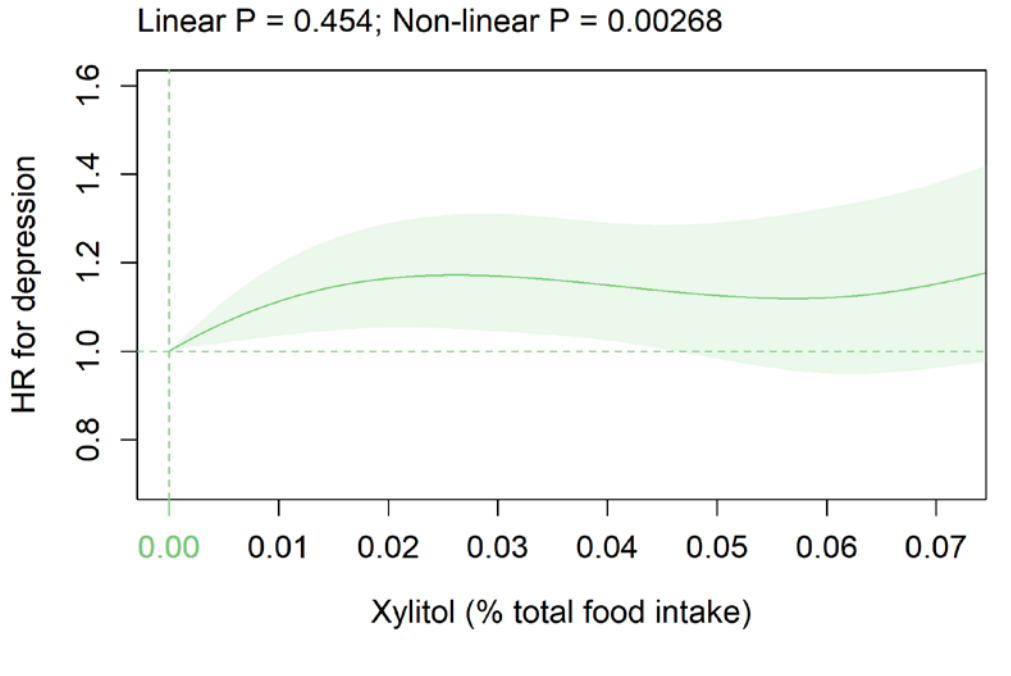

(s)

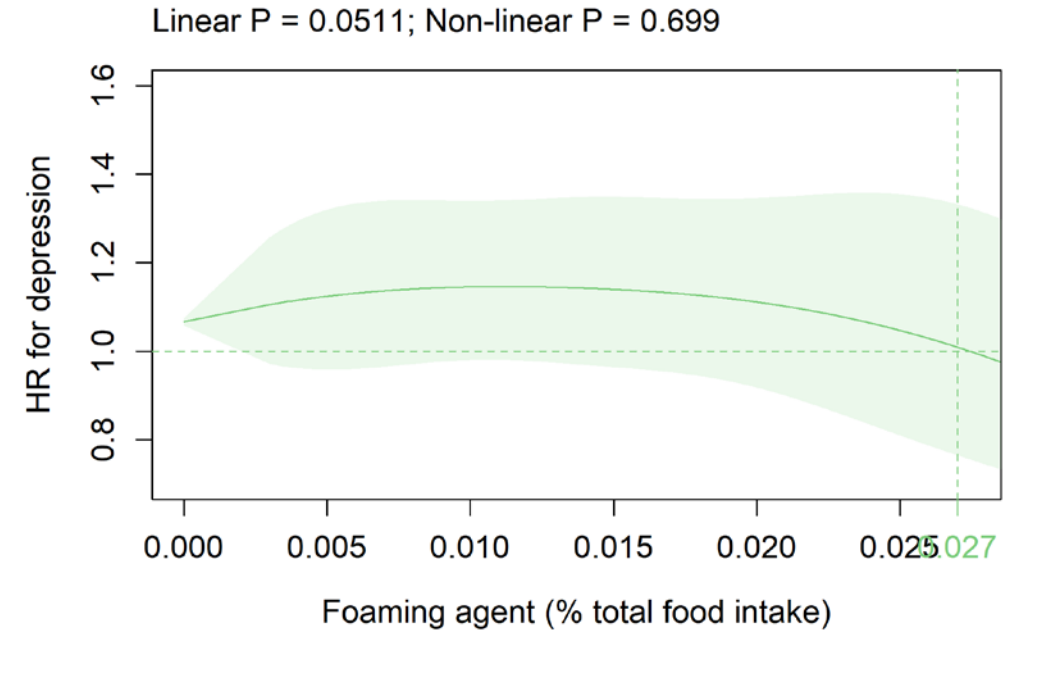

Online Resource 7 - Unintentional weight loss removed

(t)

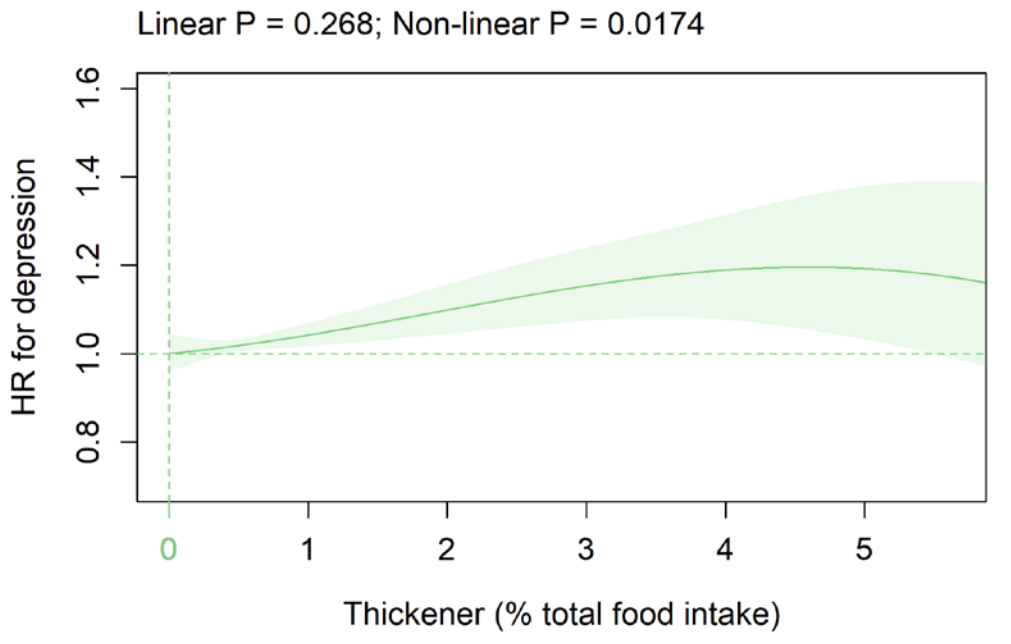

(u)

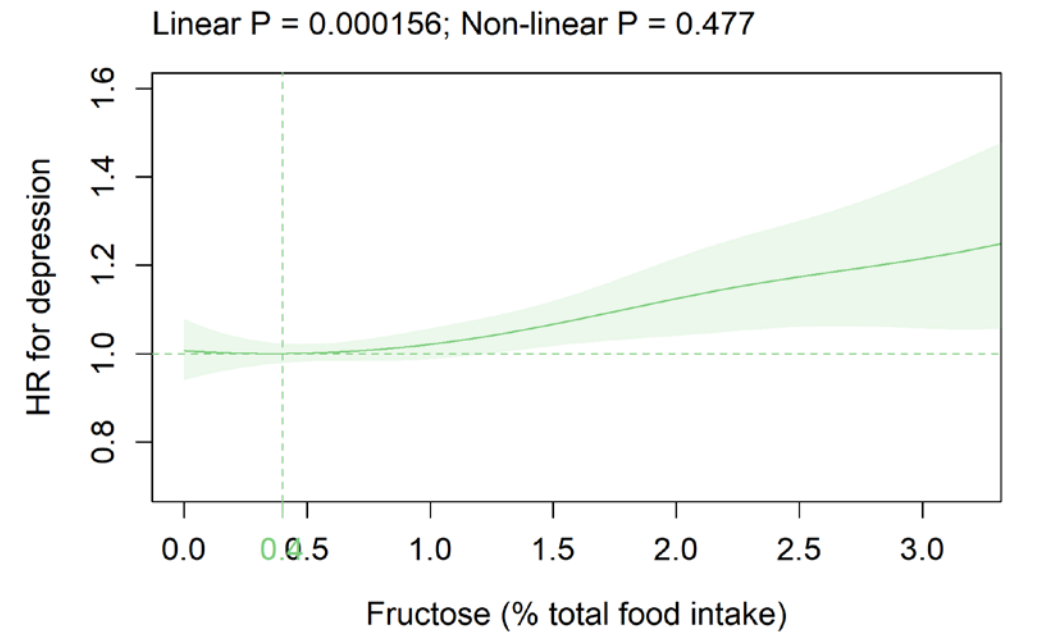

(v)

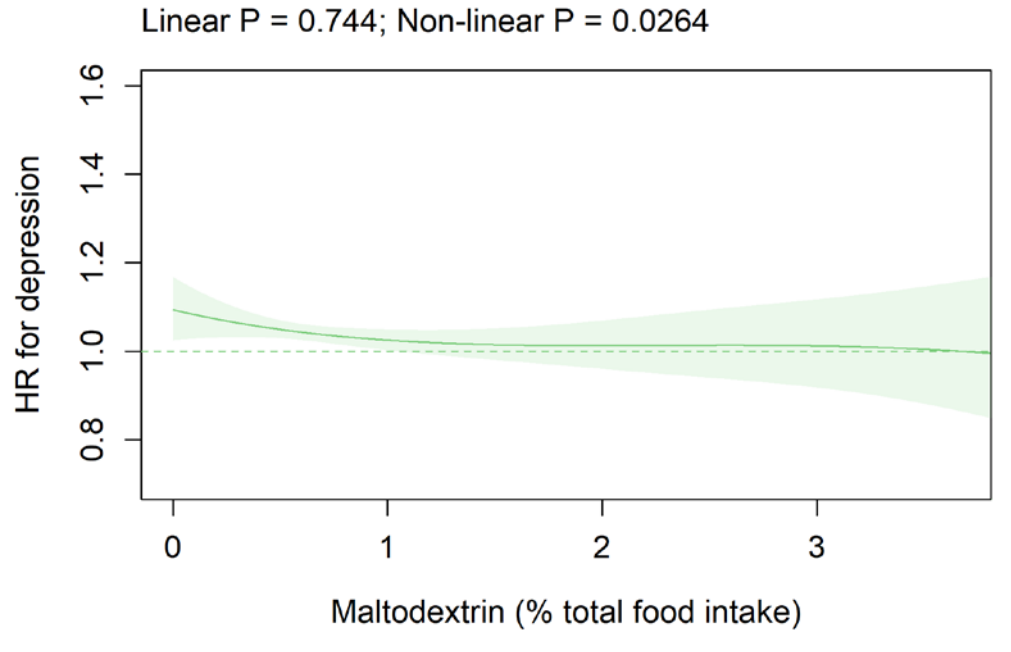

(w)

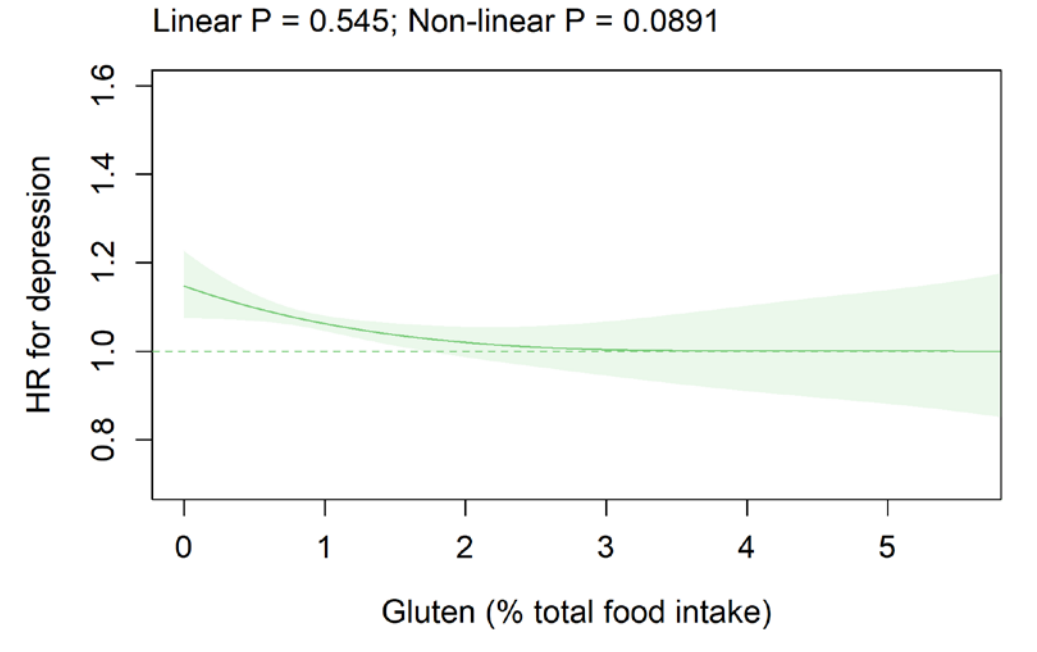

Online Resource 7 - Unintentional weight loss removed

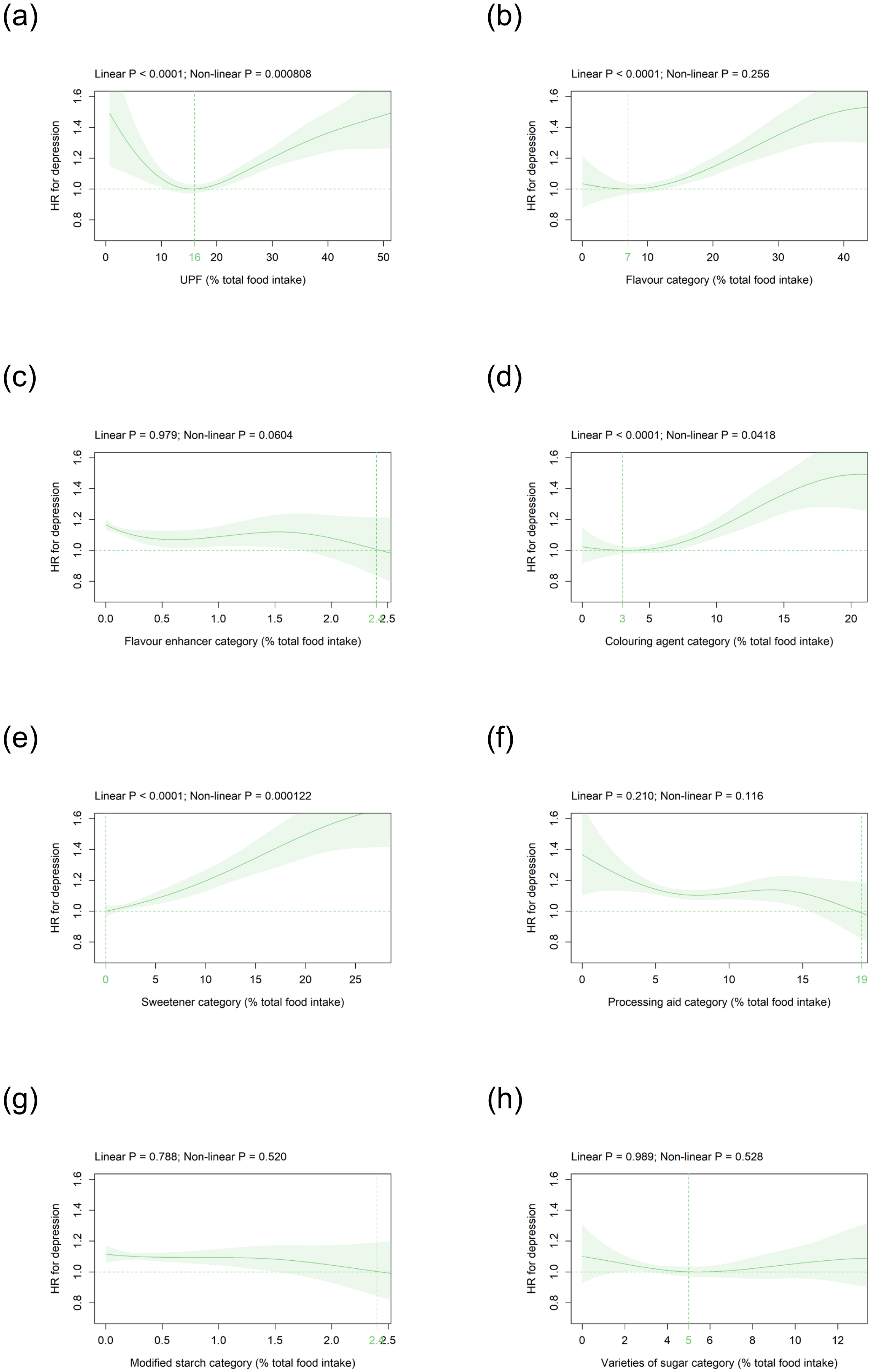

Online Resource 8 - Atypical diet excluded

(i)

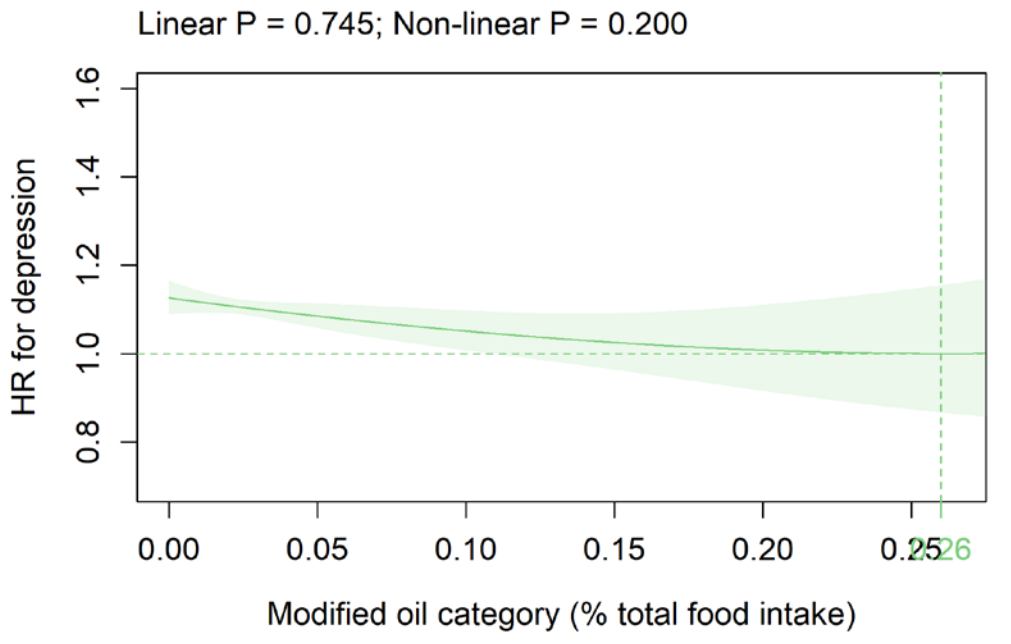

(j)

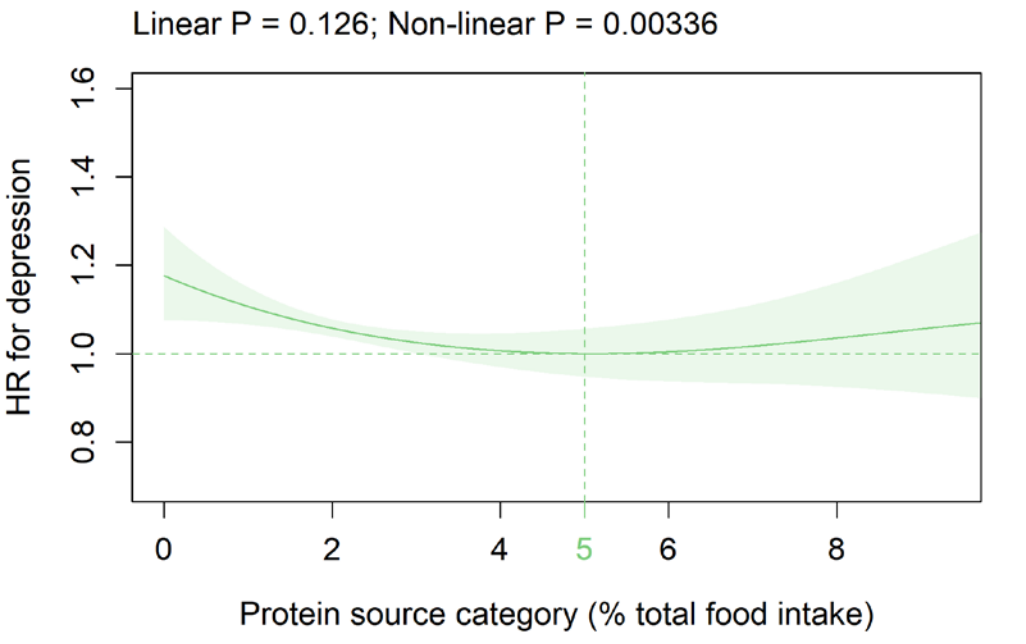

(k)

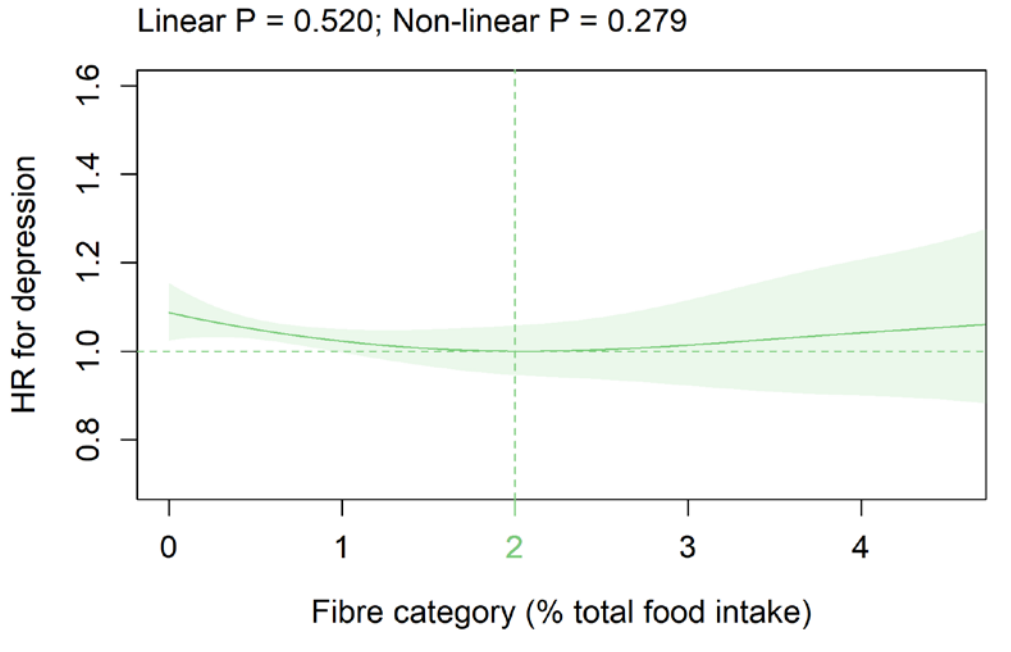

Online Resource 8 - Atypical diet excluded

(l)

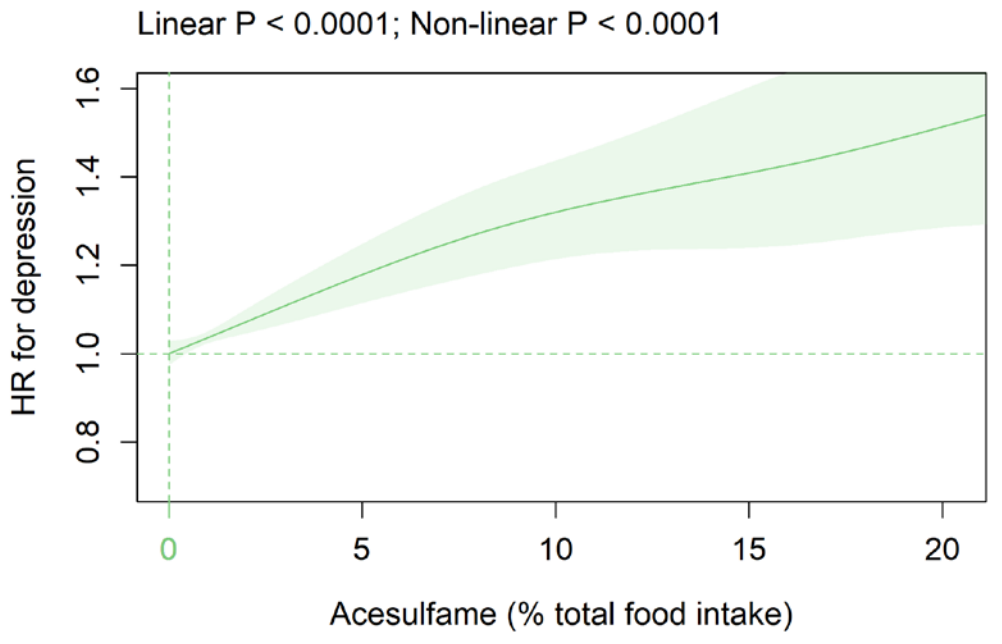

(m)

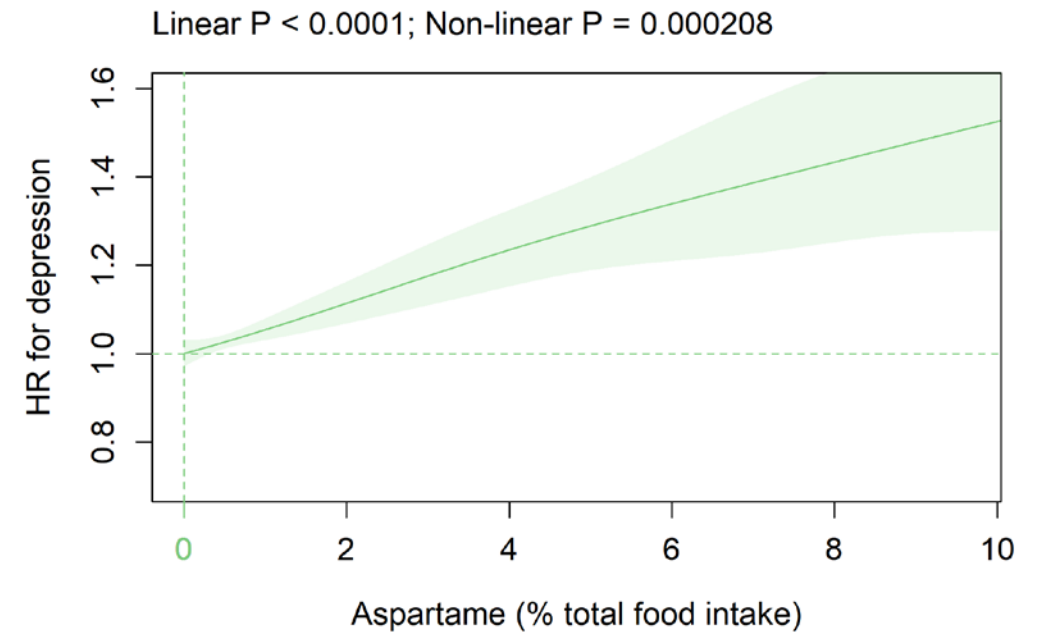

(n)

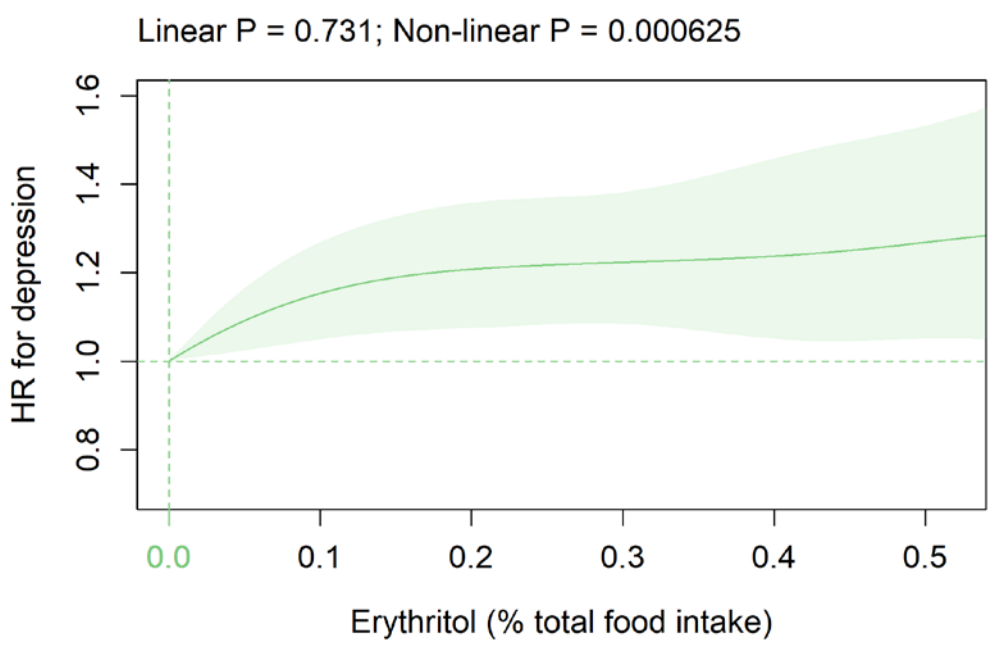

(o)

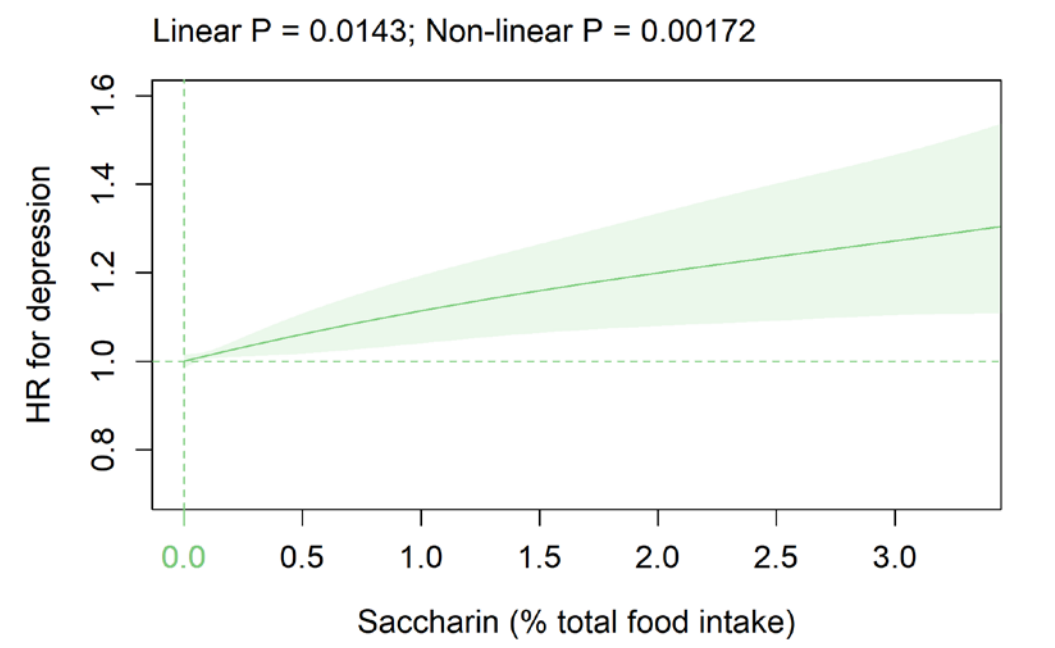

(p)

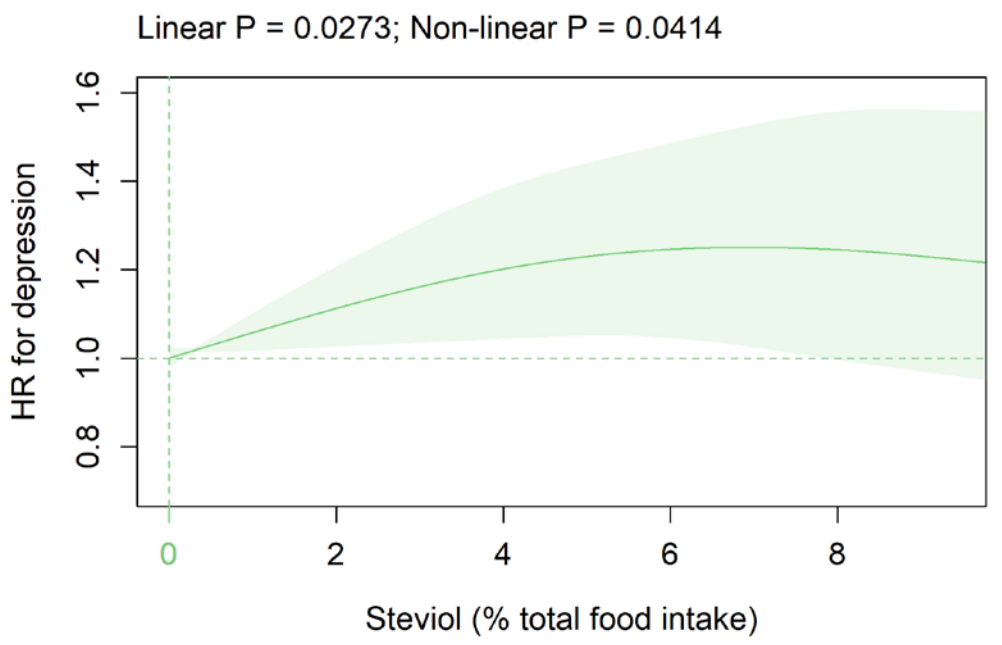

(q)

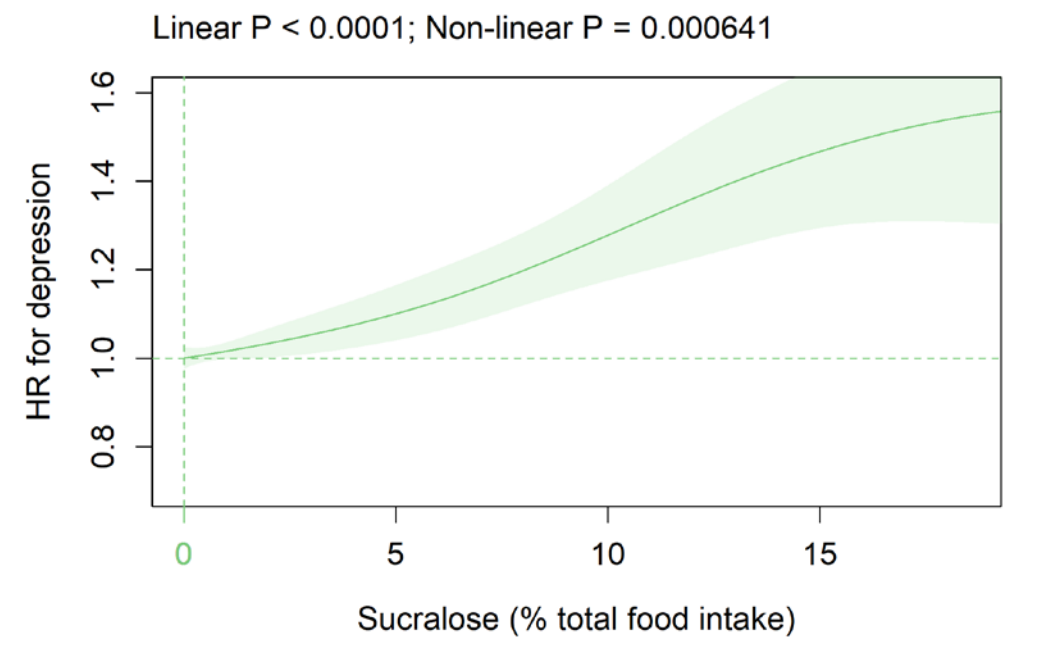

(r)

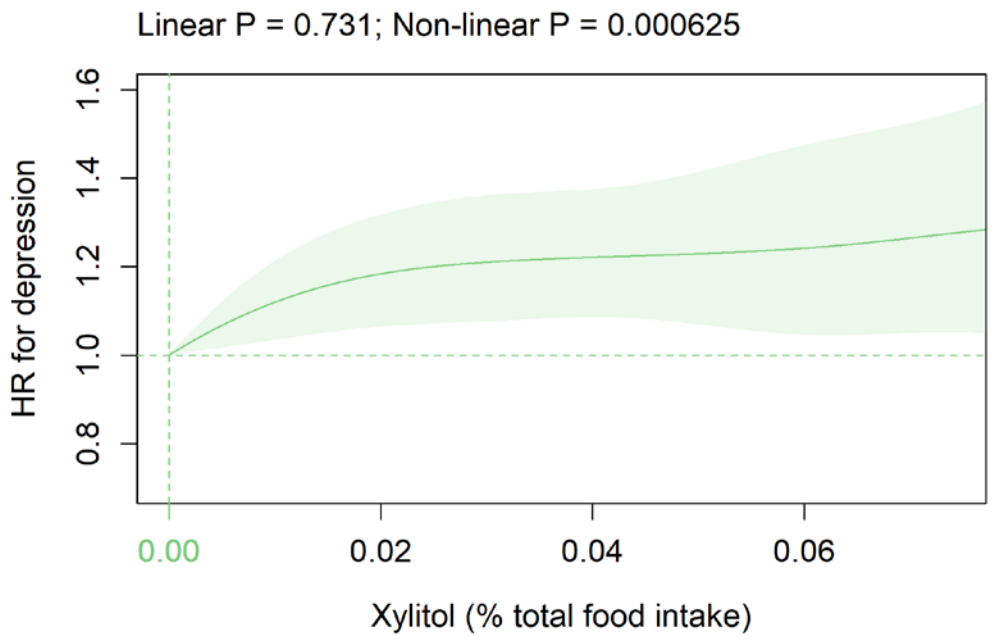

(s)

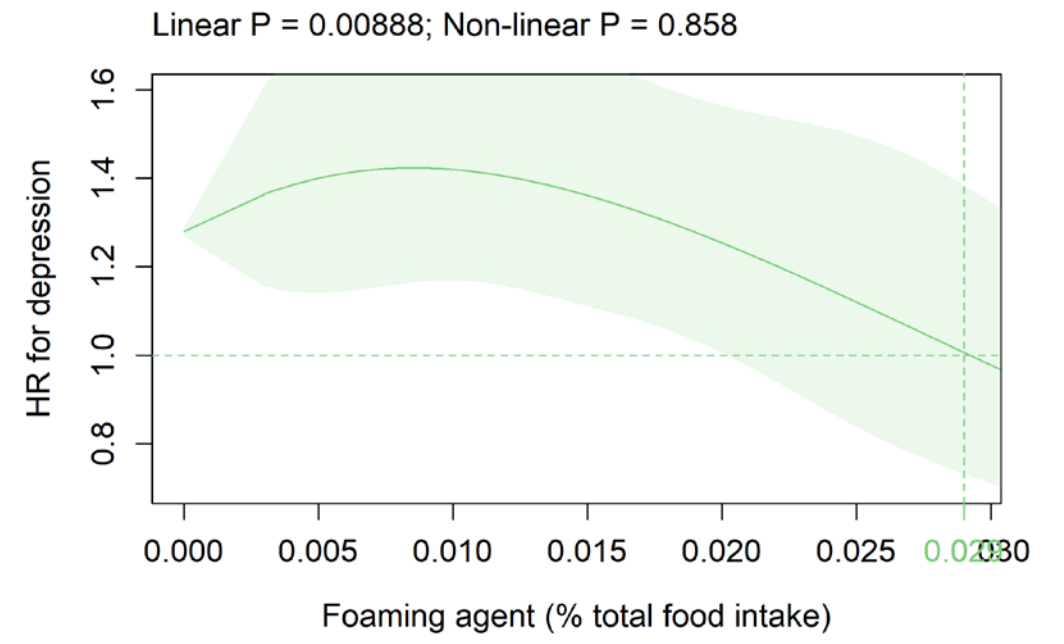

Online Resource 8 - Atypical diet excluded

(t)

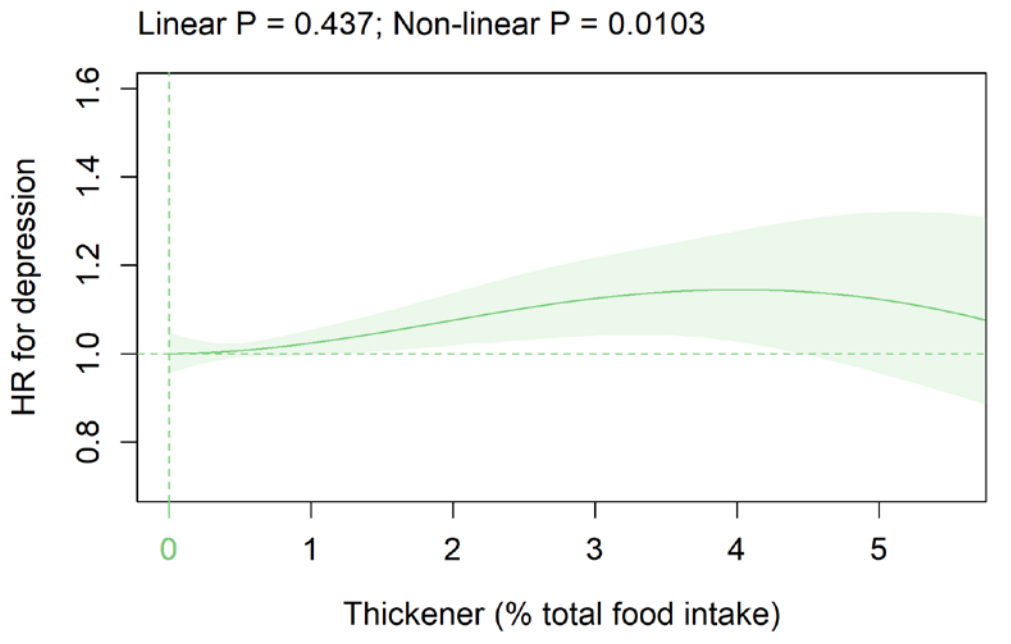

(u)

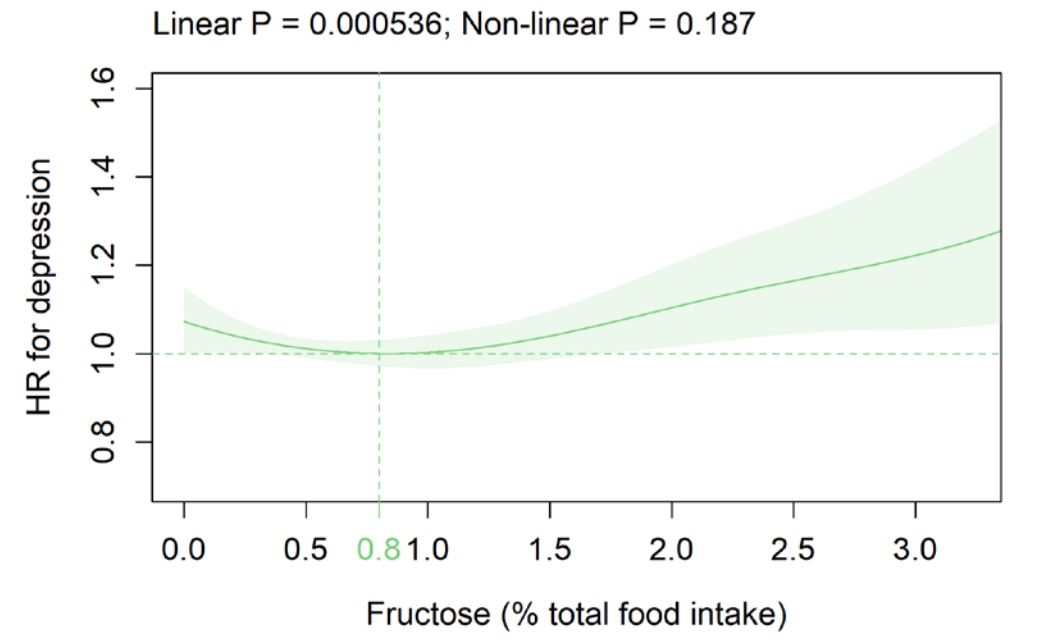

(v)

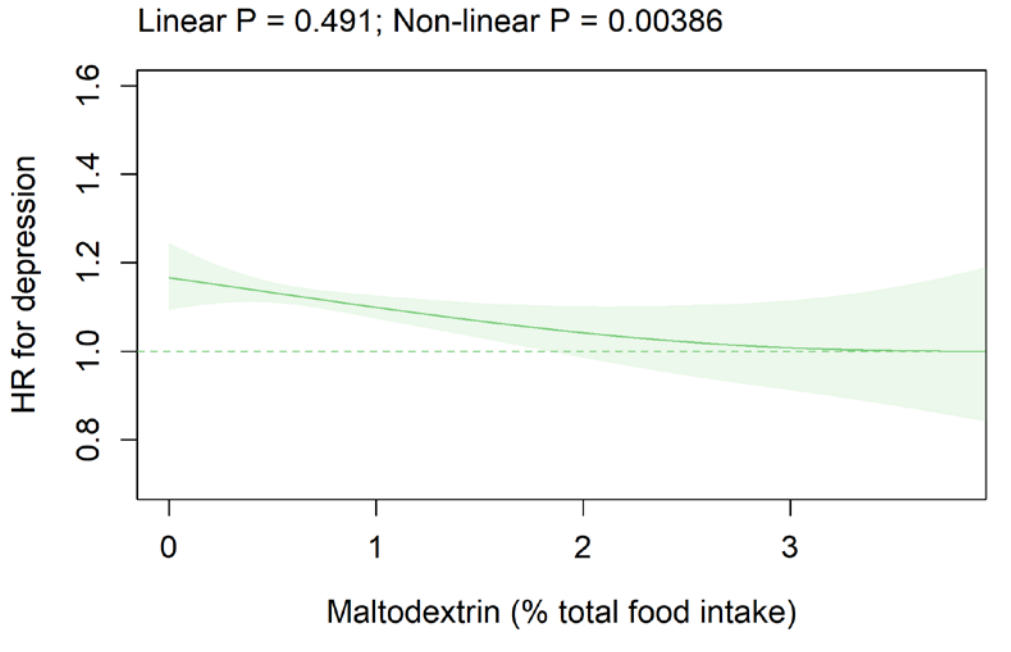

(w)

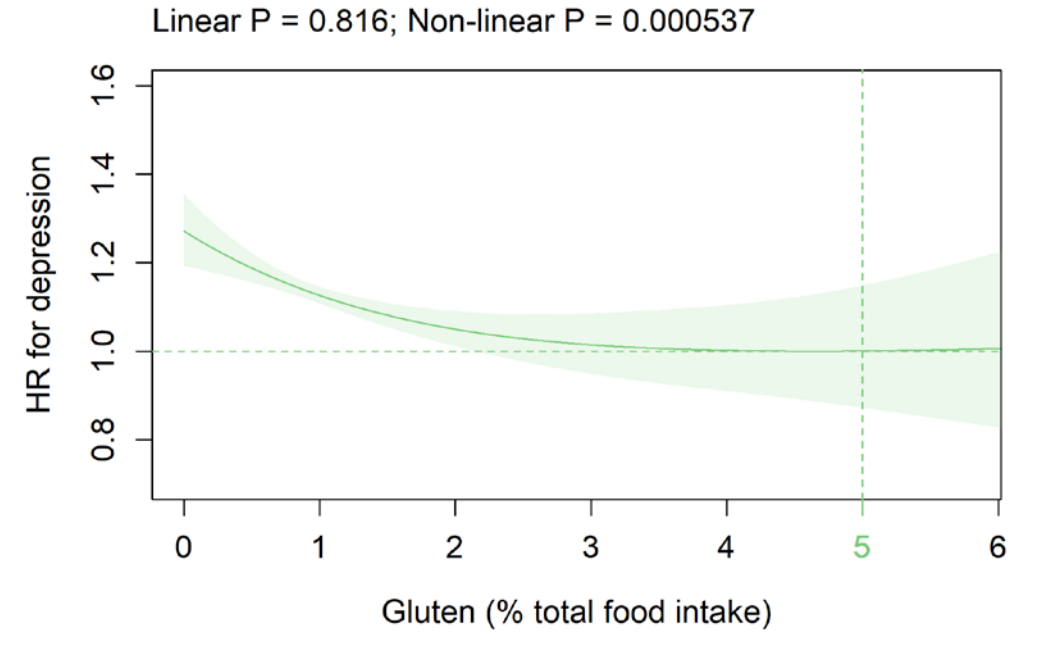

Online Resource 8 - Atypical diet excluded

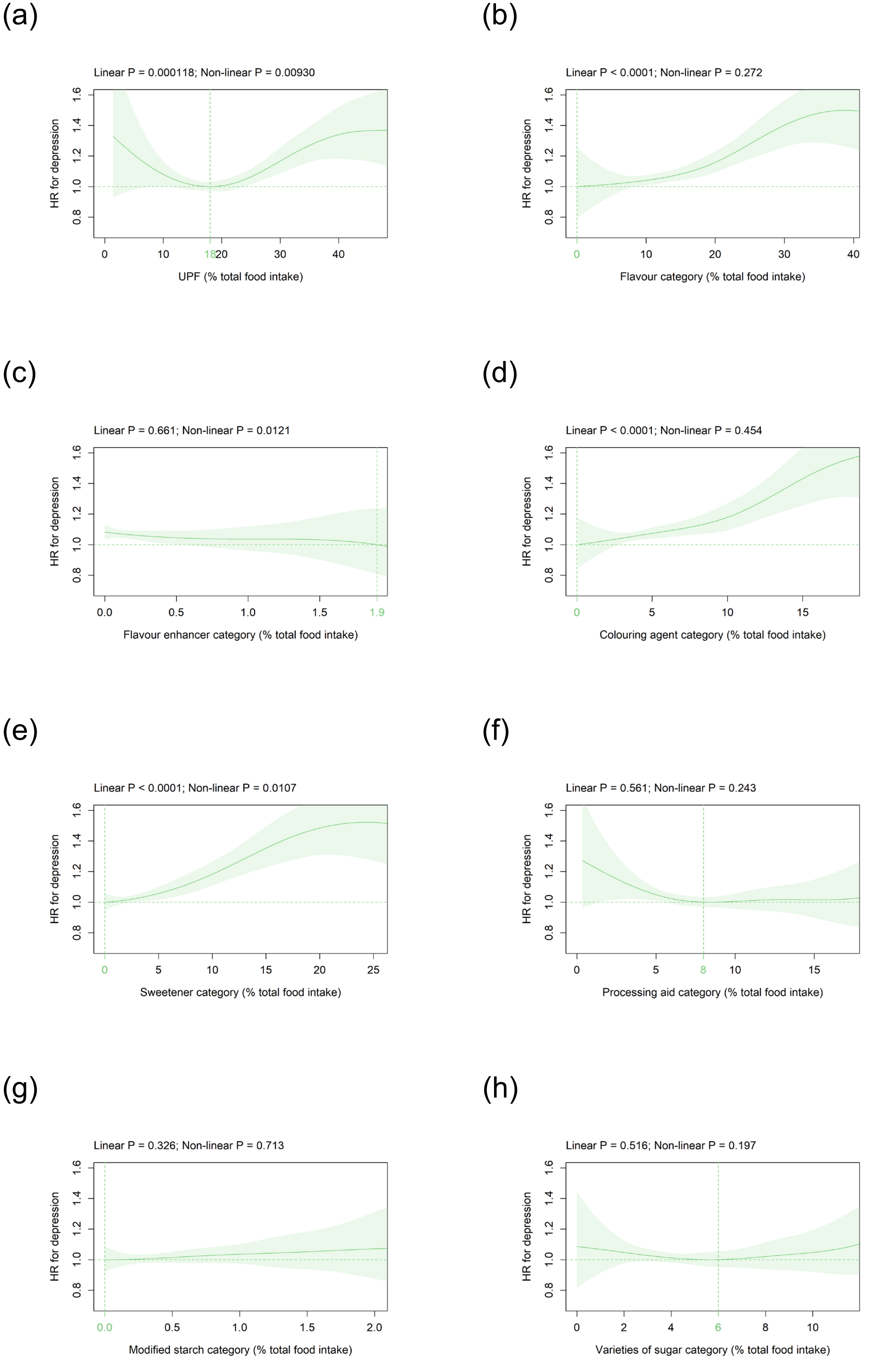

Online Resource 9 - More than one Oxford WebQ

(i)

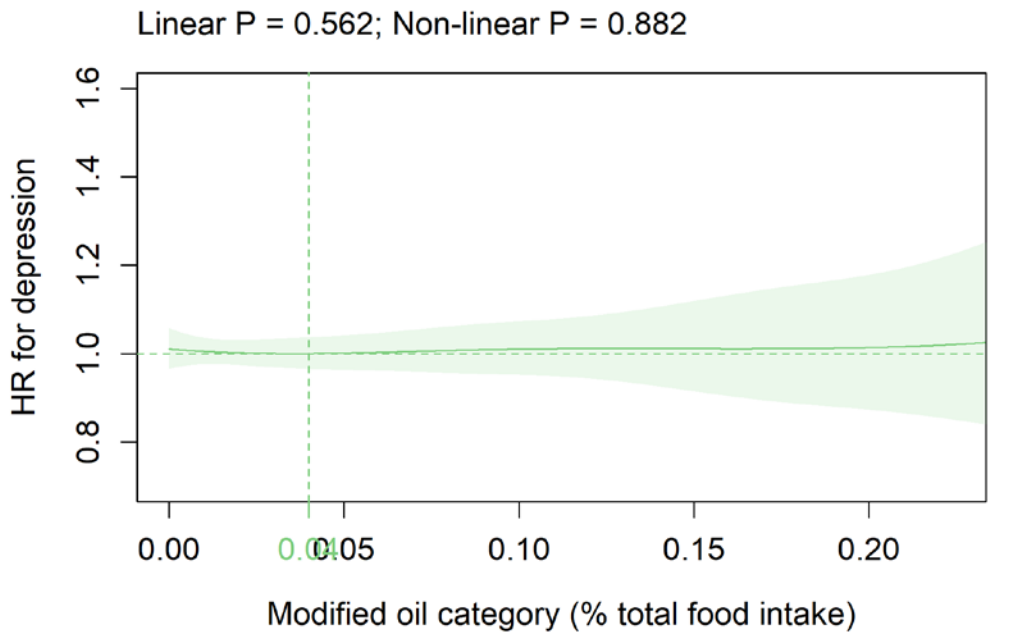

(j)

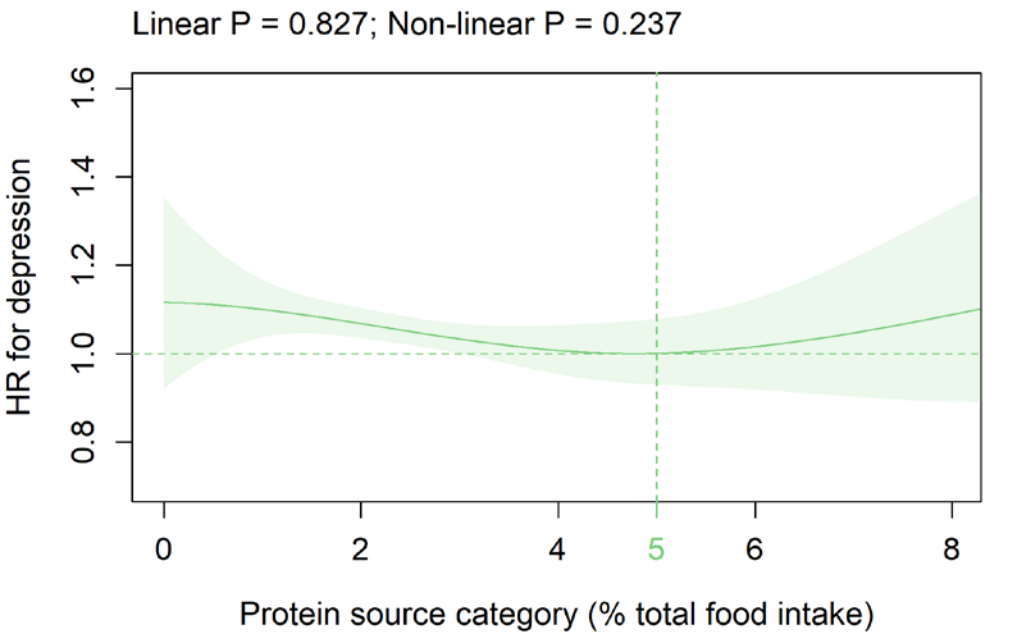

(k)

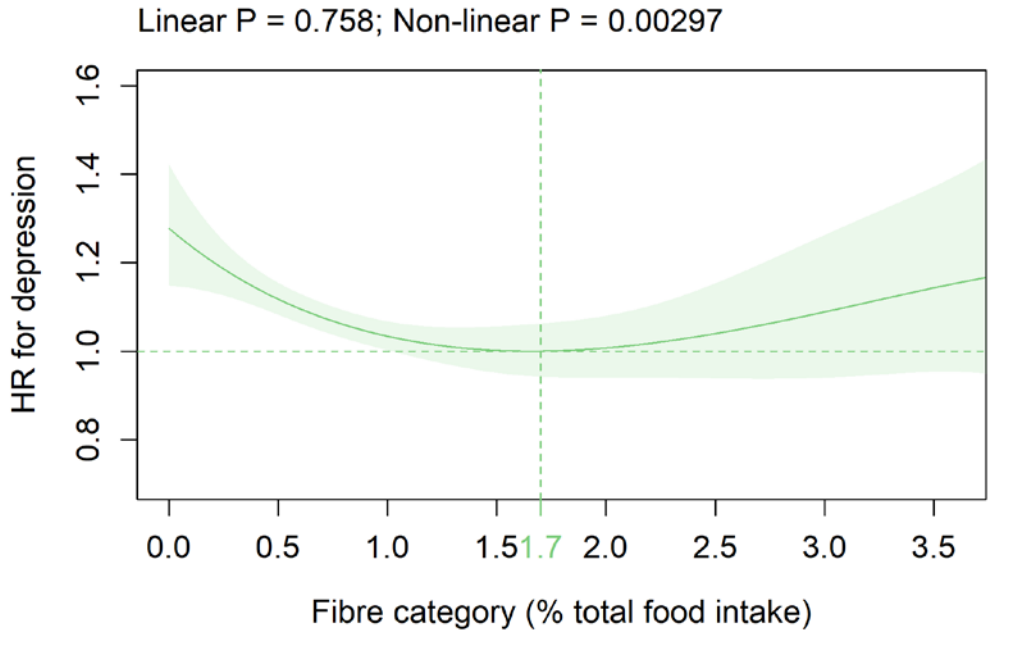

Online Resource 9 - More than one Oxford WebQ

(l)

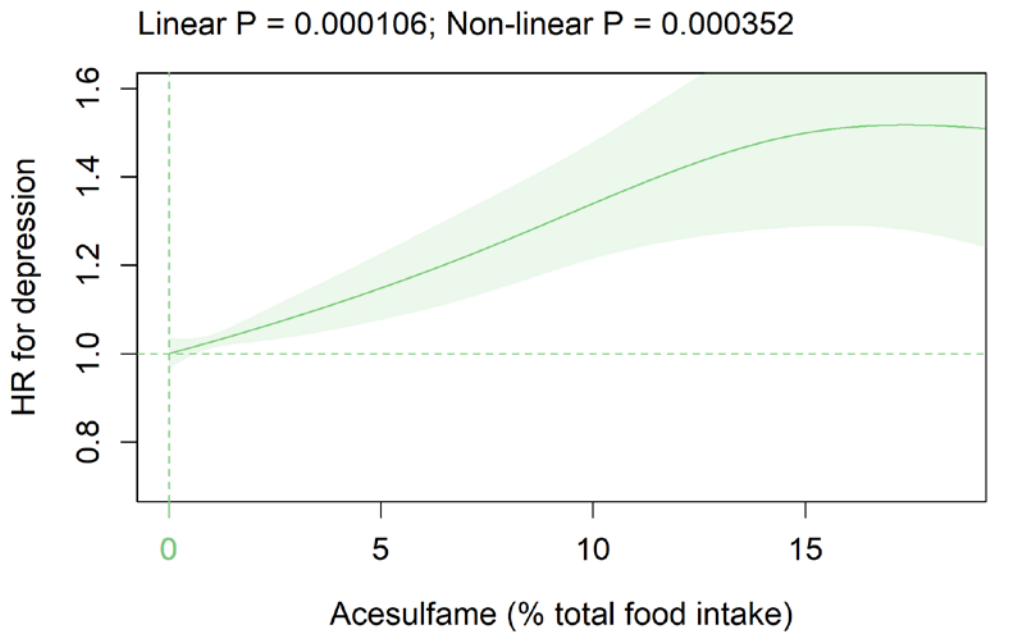

(m)

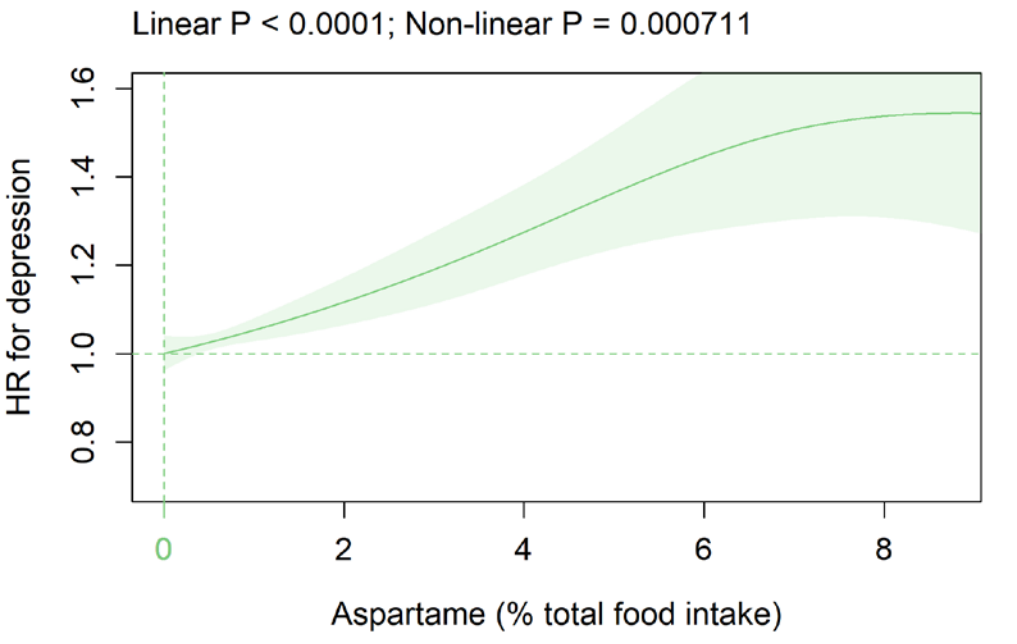

(n)

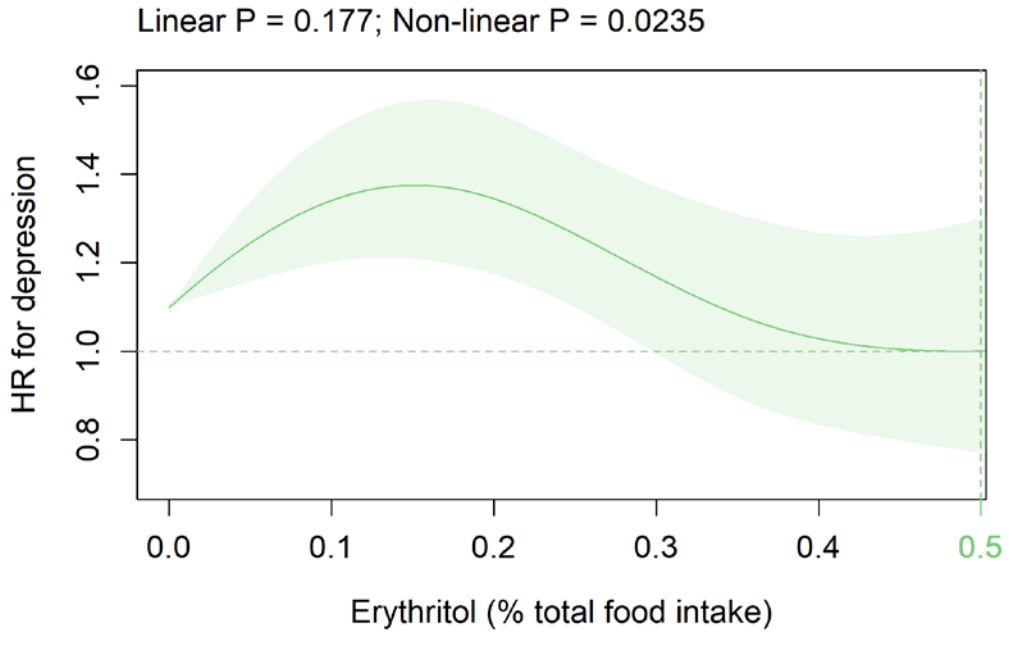

(o)

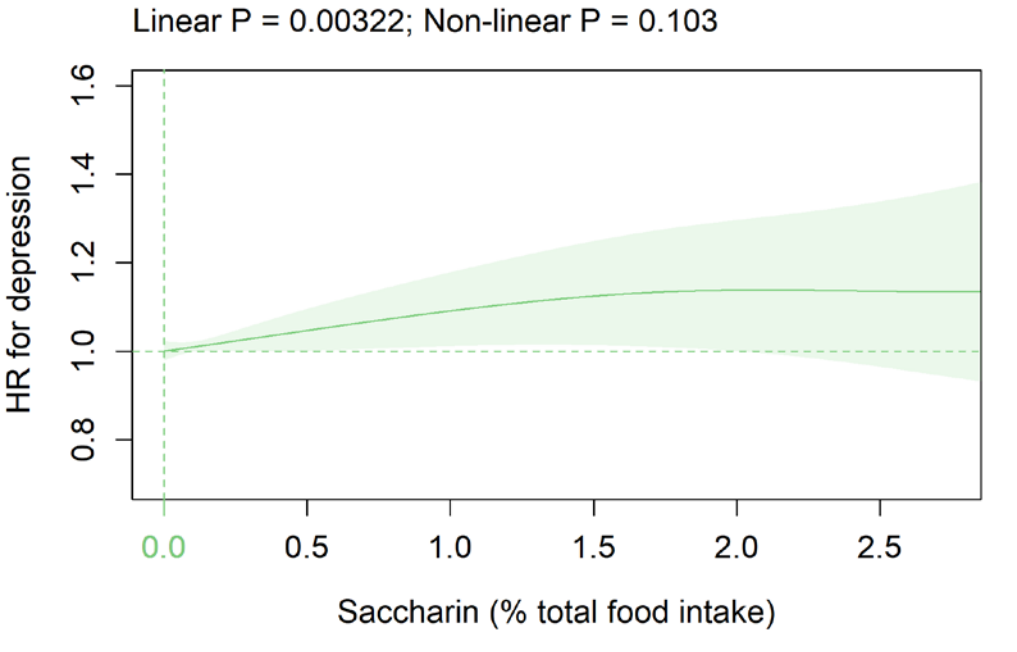

(p)

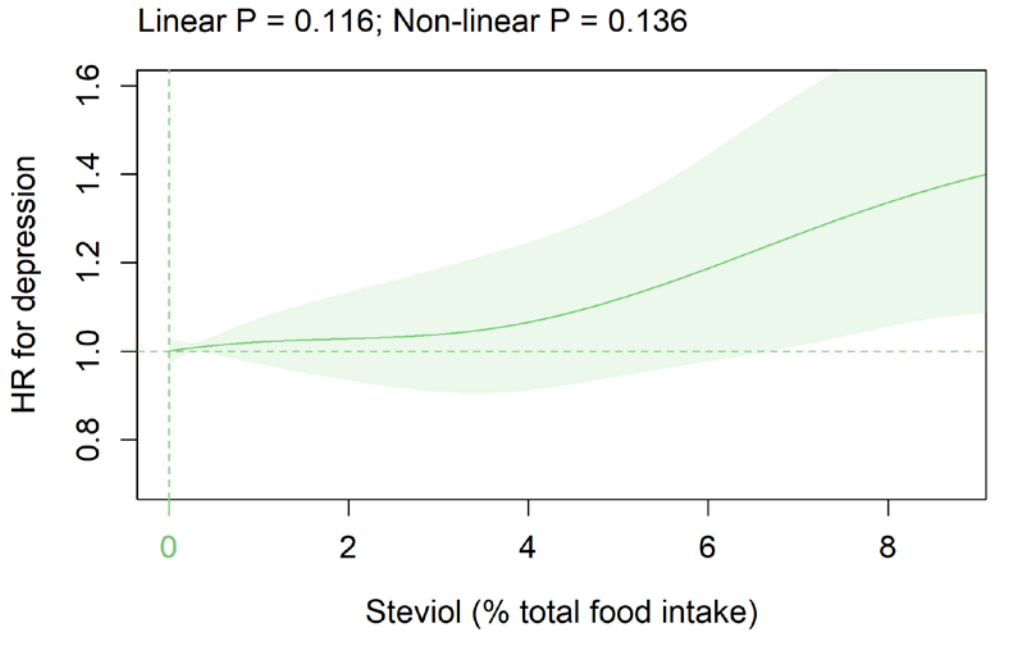

(q)

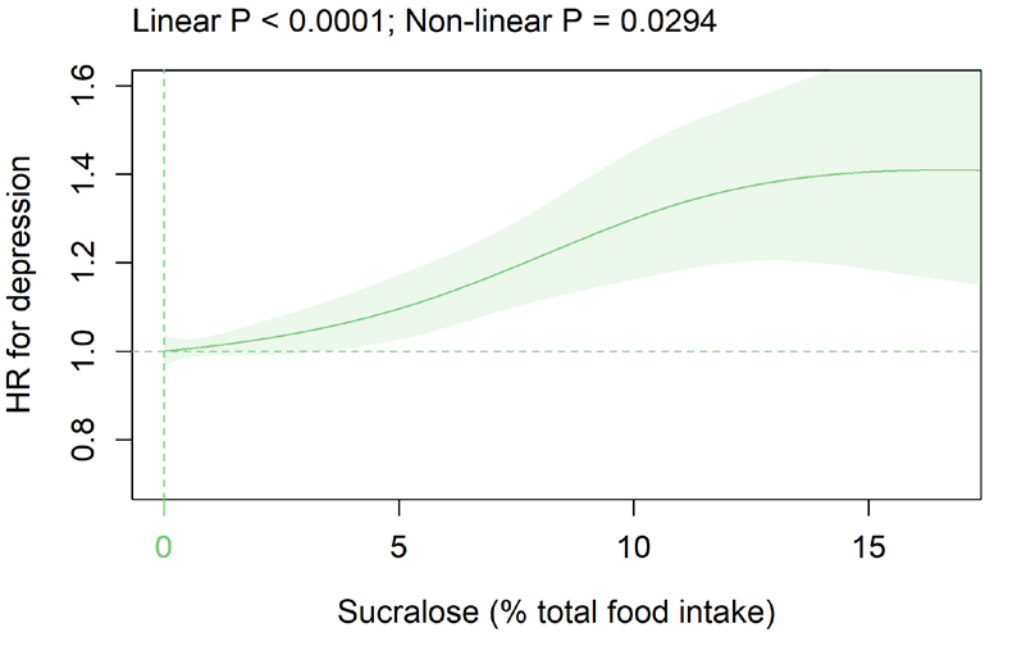

(r)

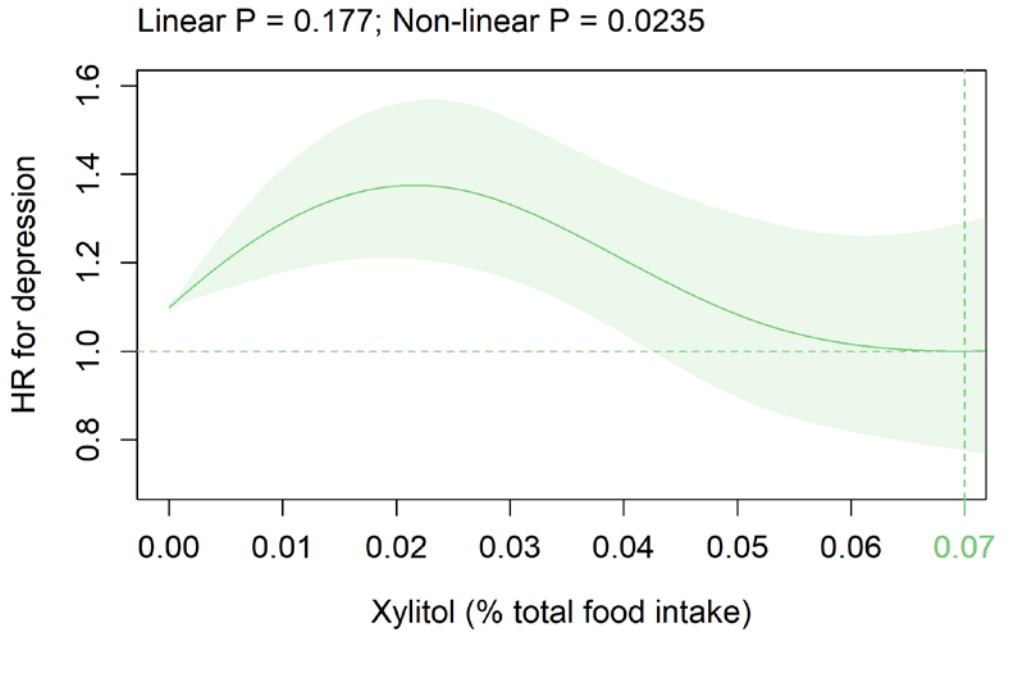

(s)

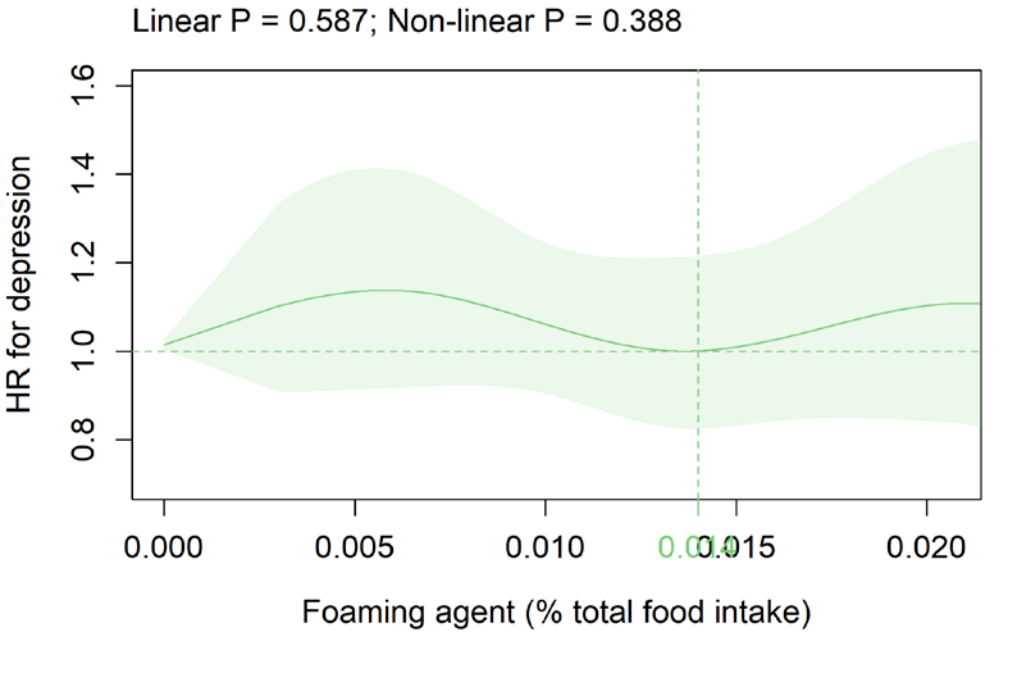

Online Resource 9 - More than one Oxford WebQ

(t)

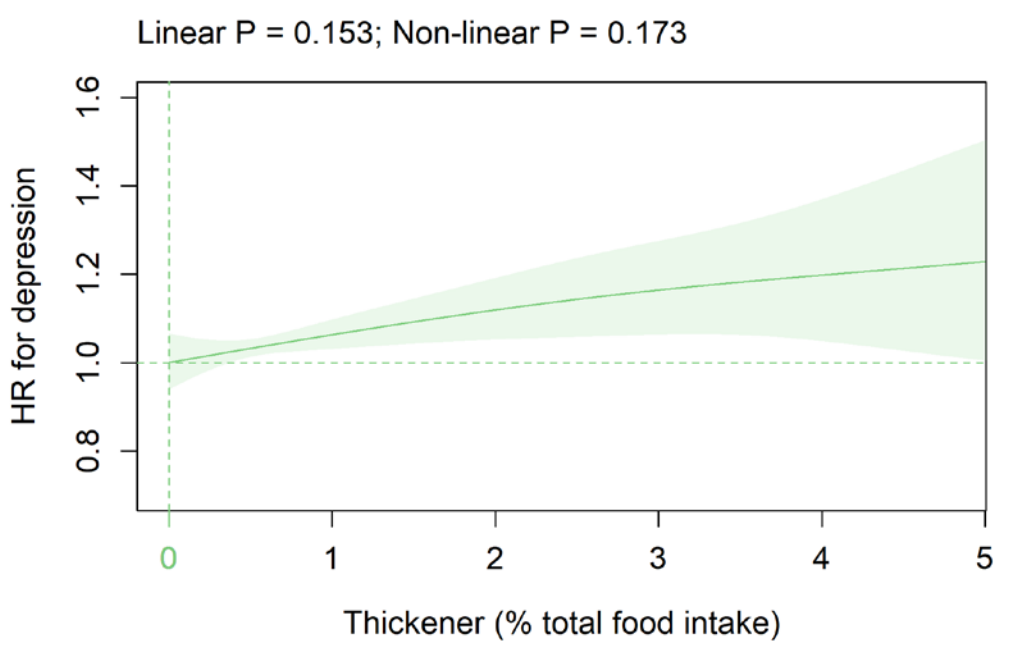
Linear P = 0.153; Non-linear P = 0.173
HR for depression
0.8
1.0
1.2
1.4
1.6
0
1
2
3
4
5
Thickener (% total food intake)

(u)

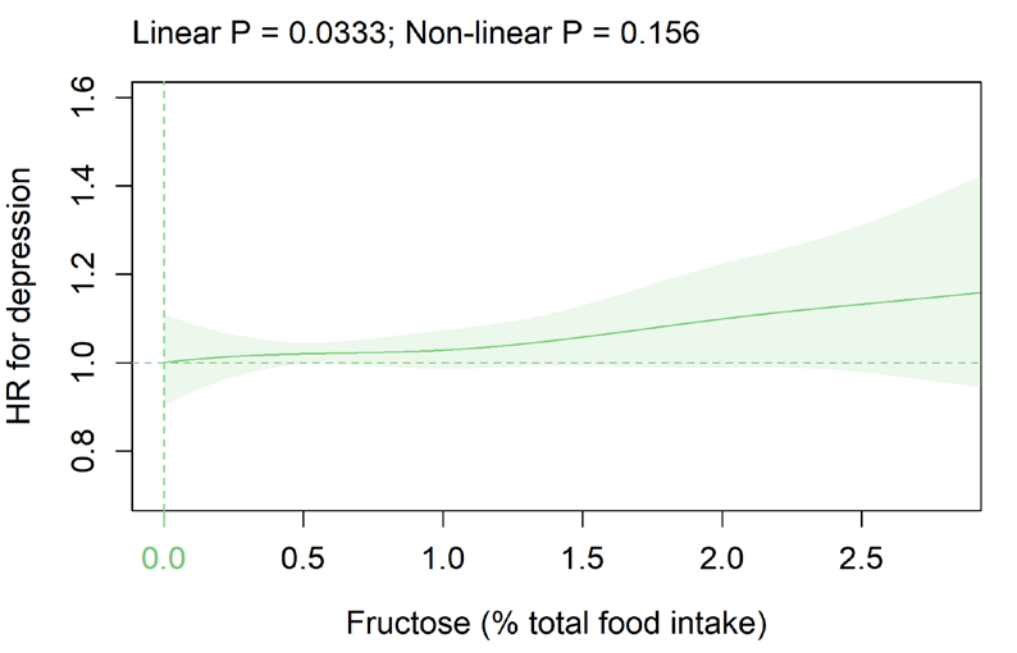
Linear P = 0.0333; Non-linear P = 0.156
HR for depression
0.8
1.0
1.2
1.4
1.6
0.0
0.5
1.0
1.5
2.0
2.5
Fructose (% total food intake)

(v)

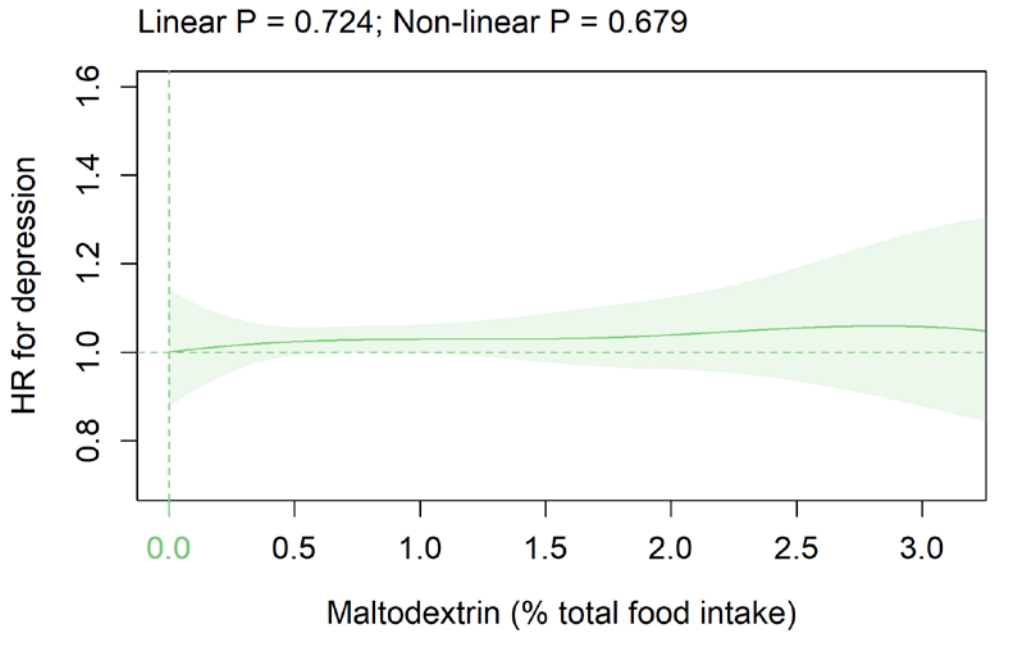
Linear P = 0.724; Non-linear P = 0.679
HR for depression
0.8
1.0
1.2
1.4
1.6
0.0
0.5
1.0
1.5
2.0
2.5
3.0
Maltodextrin (% total food intake)

(w)

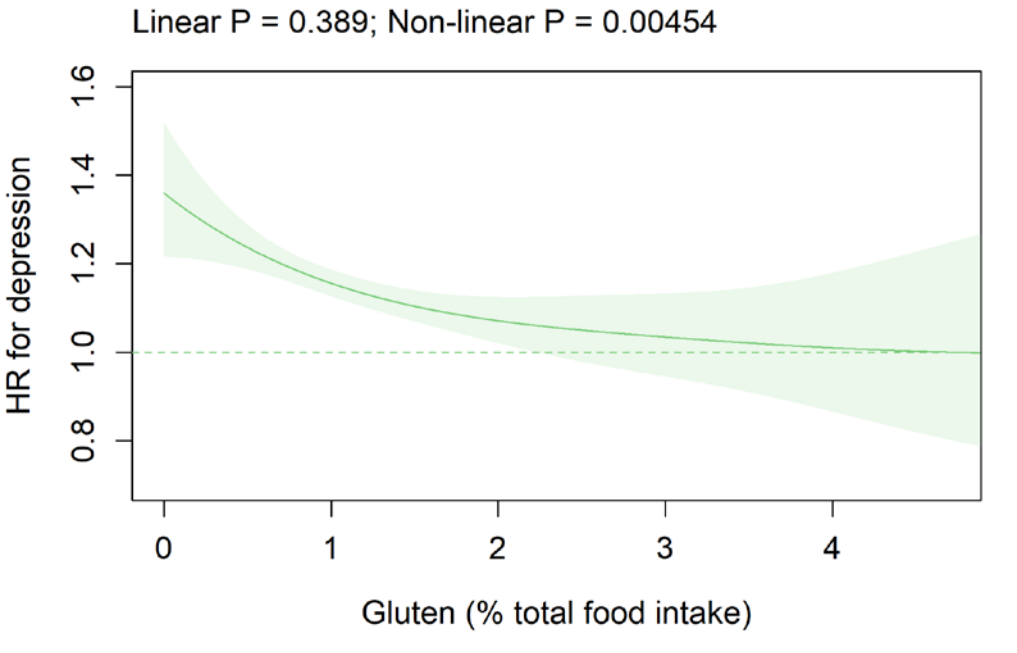
Linear P = 0.389; Non-linear P = 0.00454
HR for depression
0.8
1.0
1.2
1.4
1.6
0
1
2
3
4
Gluten (% total food intake)

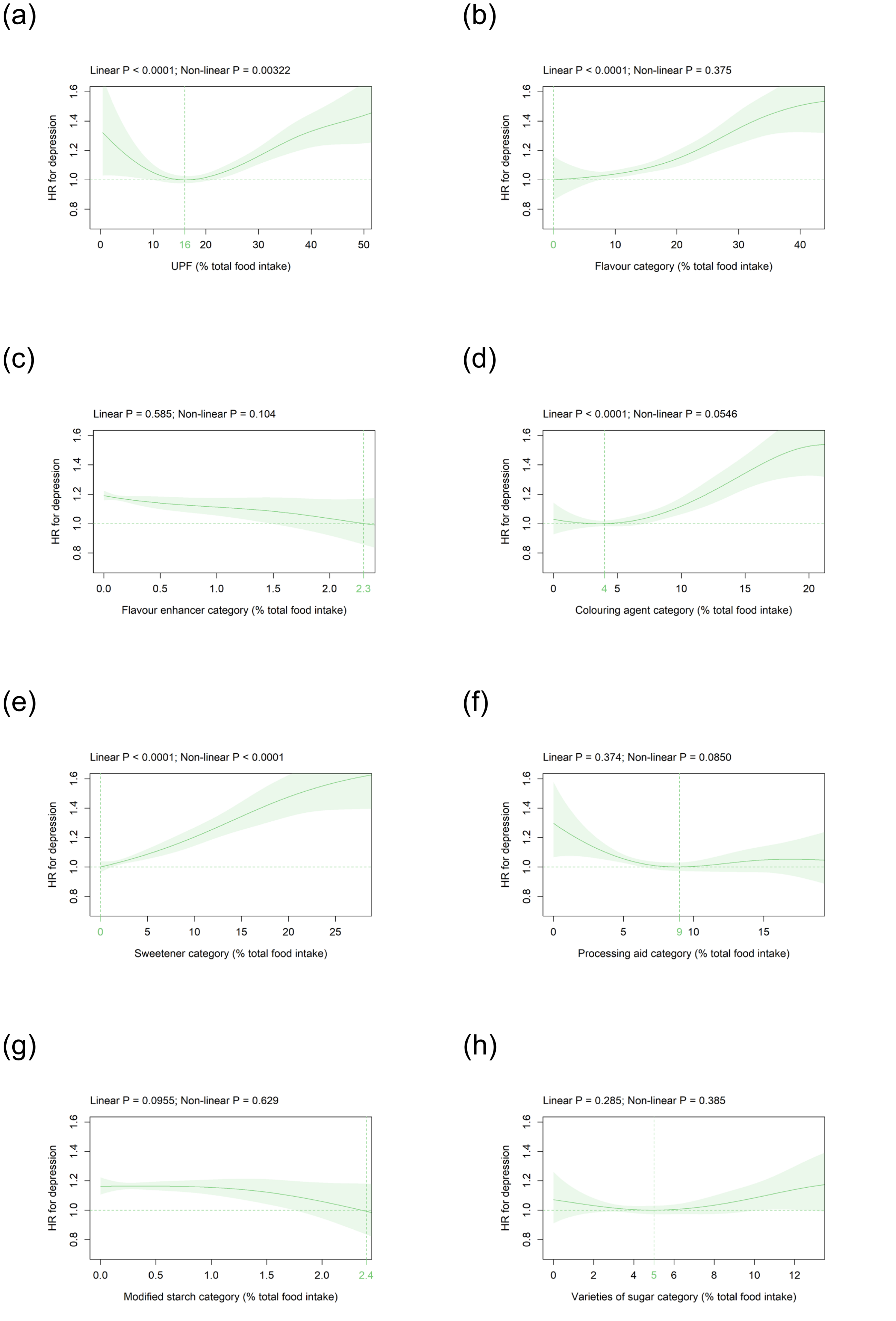

Online Resource 10 - History of CVD and cancer excluded

(i)

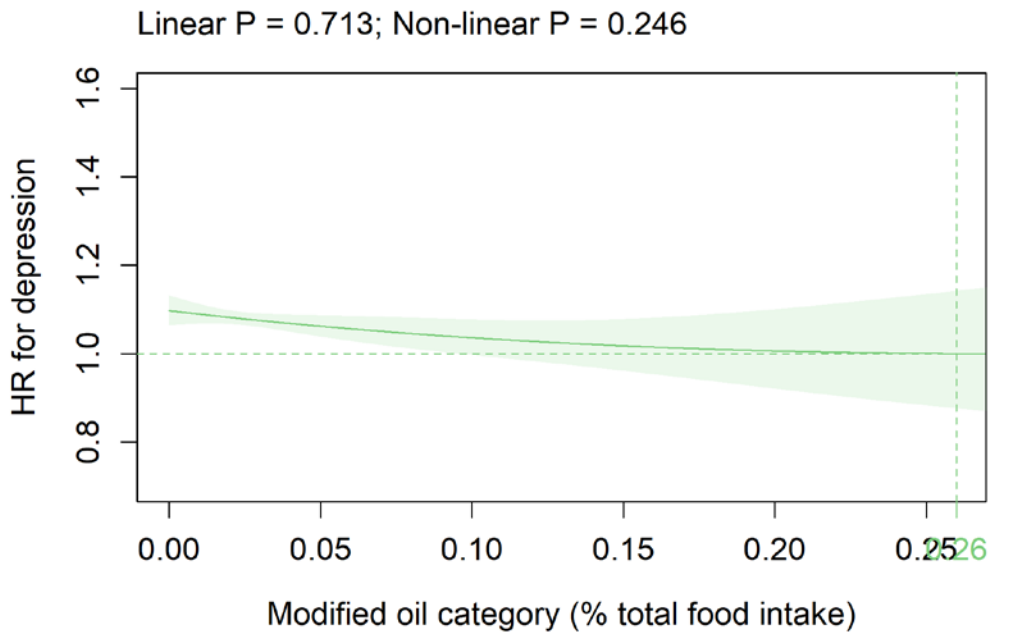

(j)

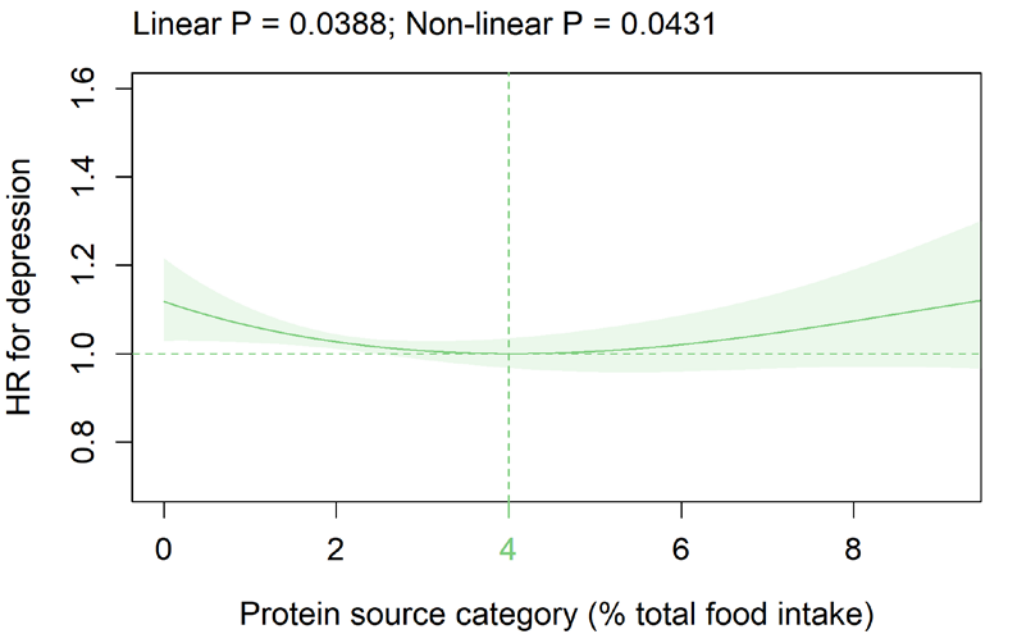

(k)

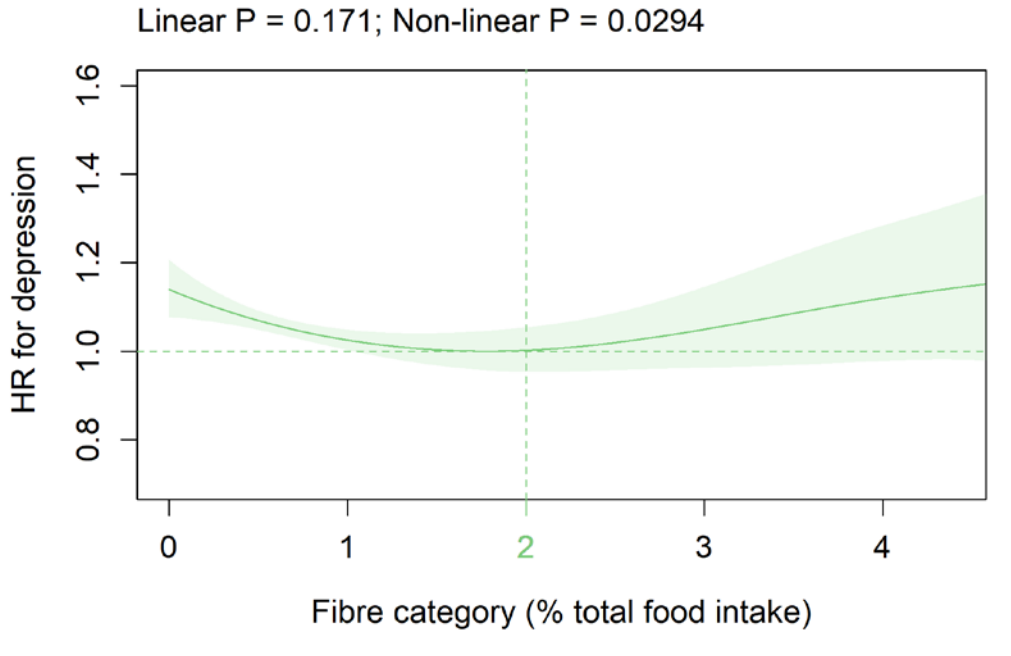

Online Resource 10 - History of CVD and cancer excluded

(l)

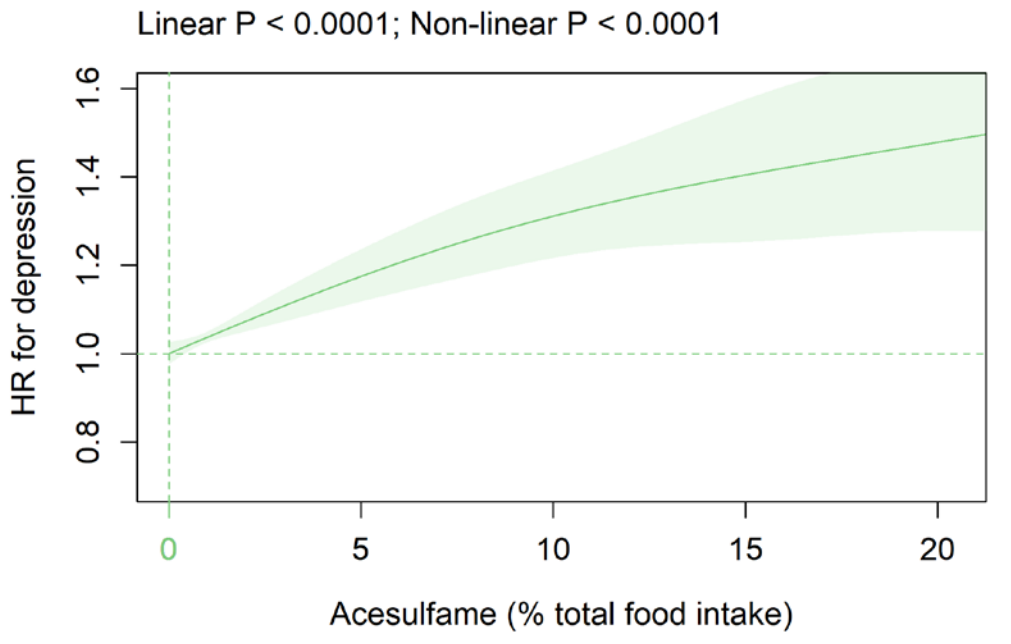

(m)

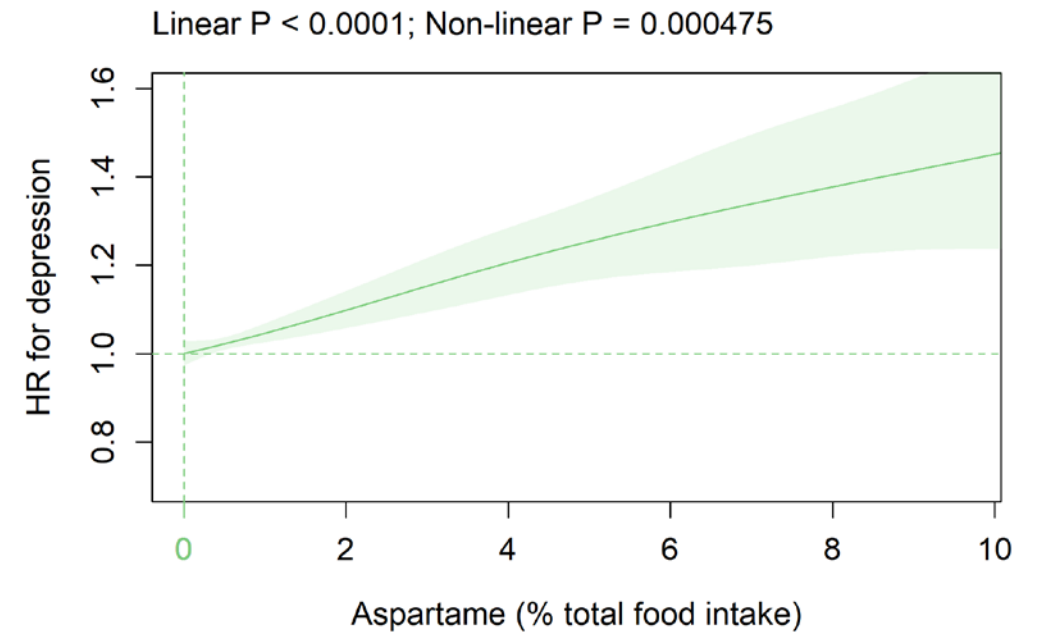

(n)

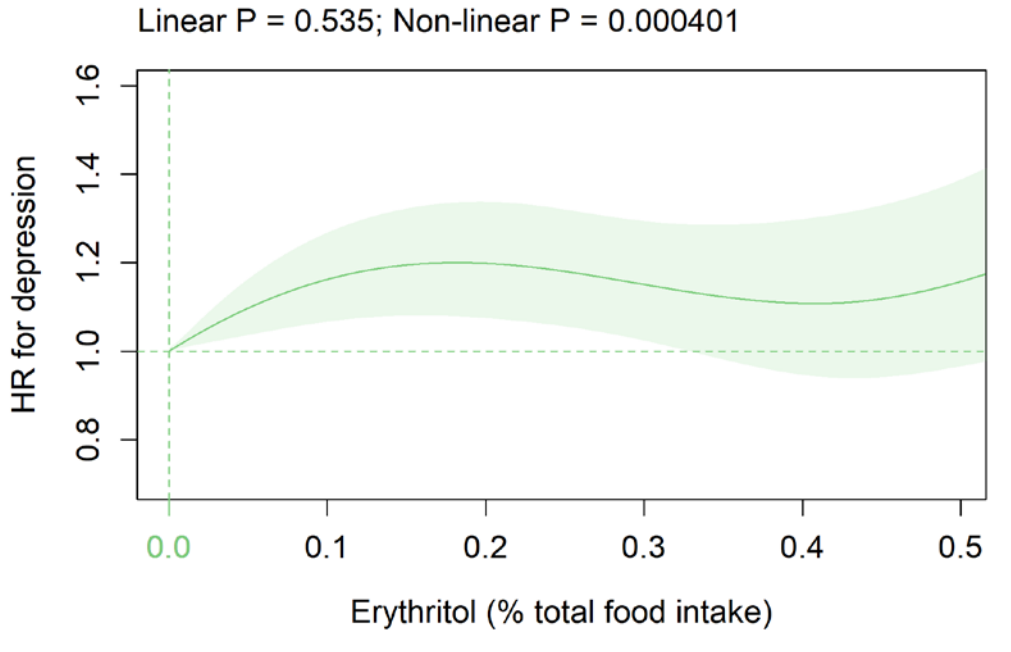

(o)

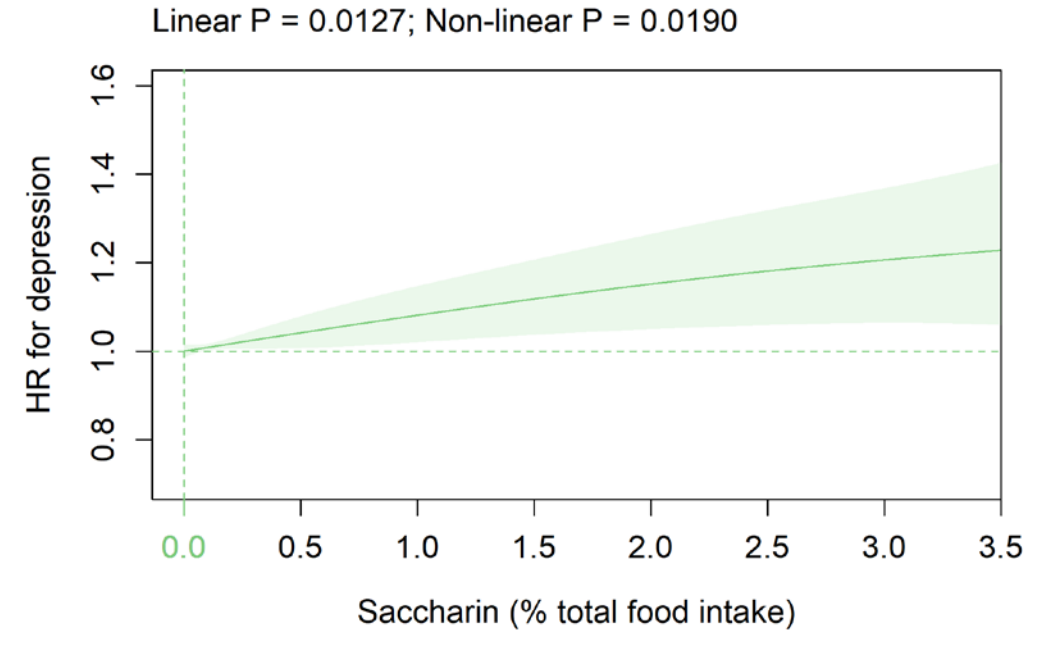

(p)

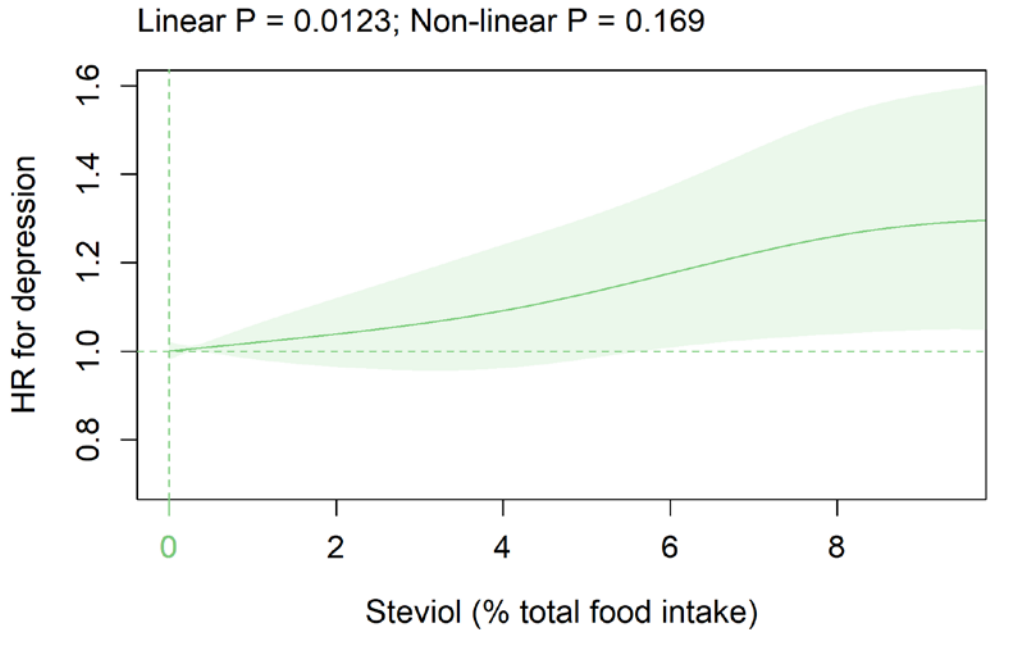

(q)

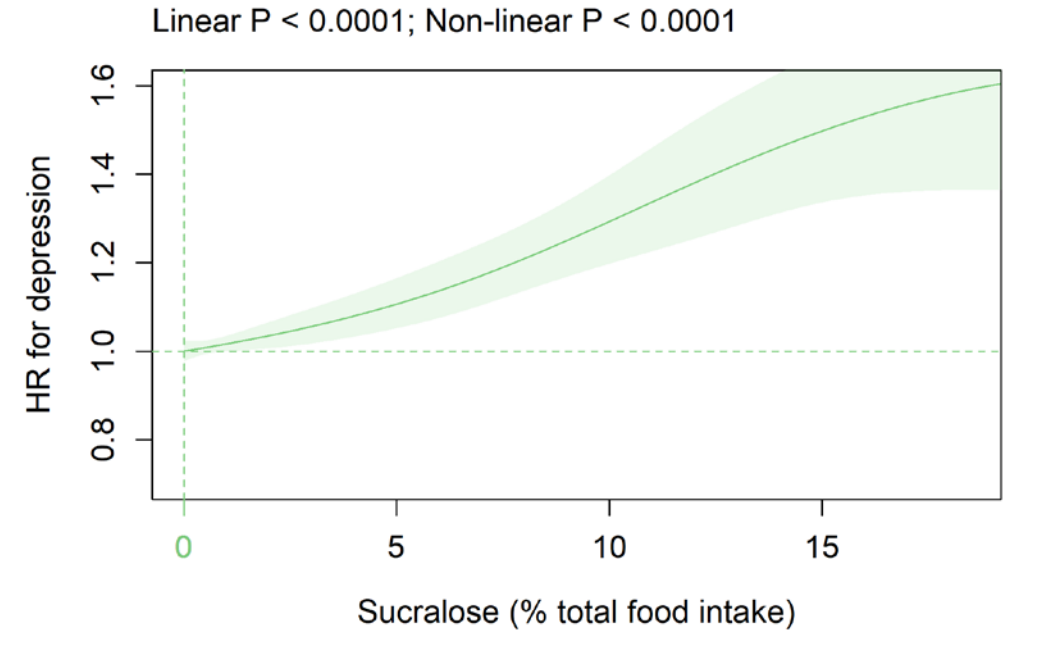

(r)

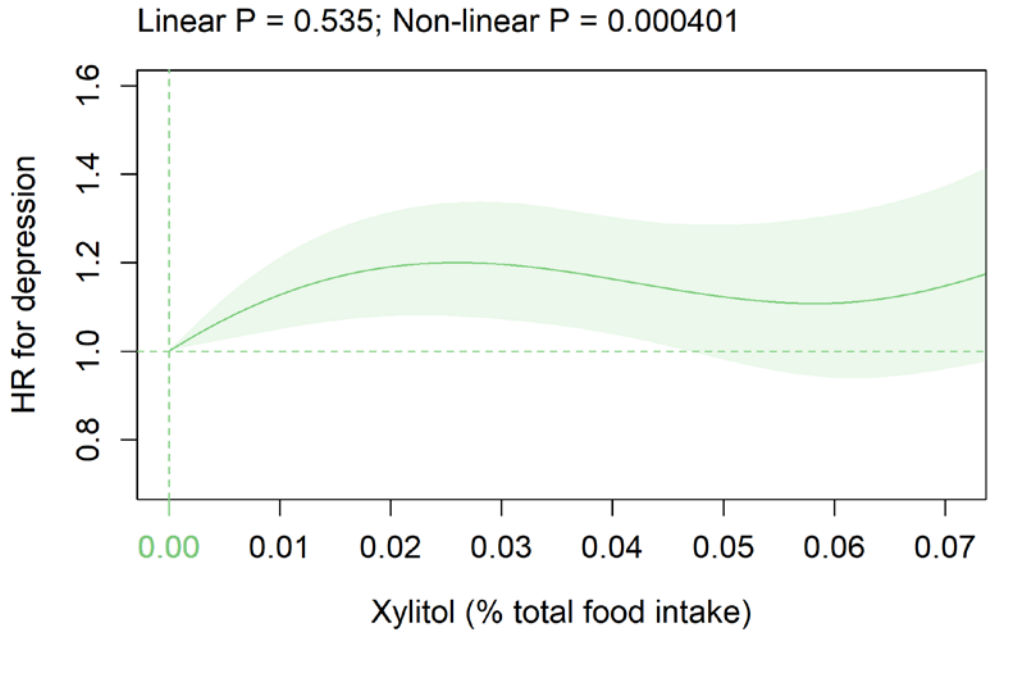

(s)

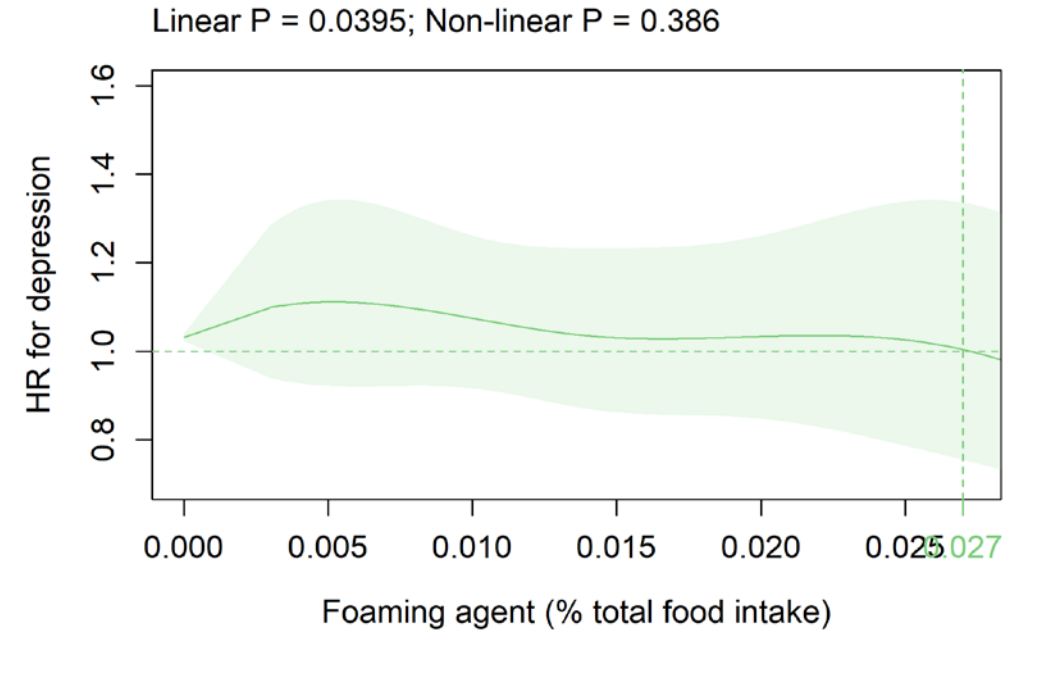

Online Resource 10 - History of CVD and cancer excluded

(t)

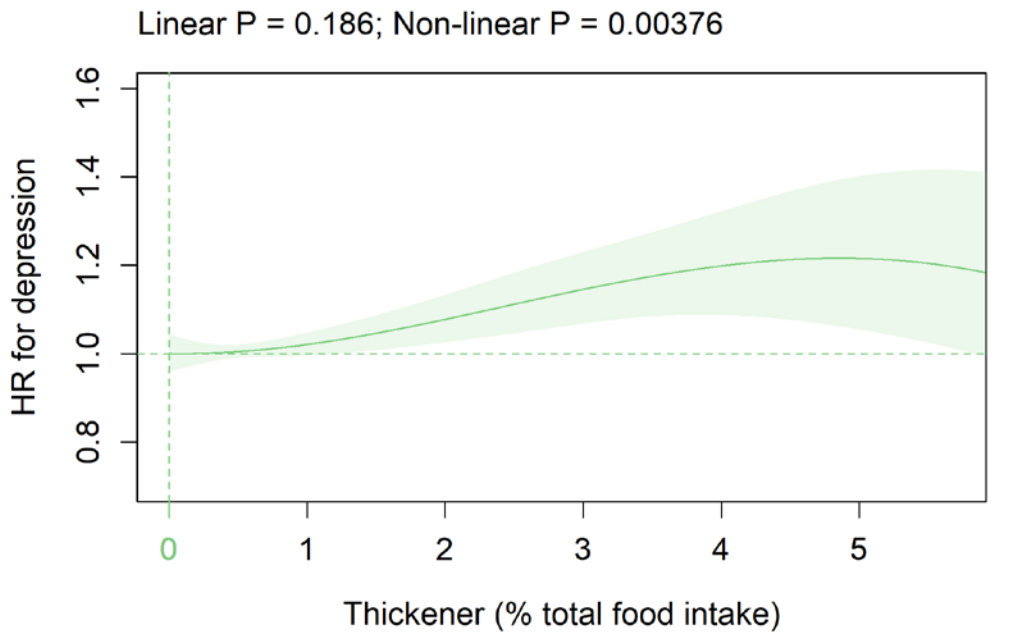

(u)

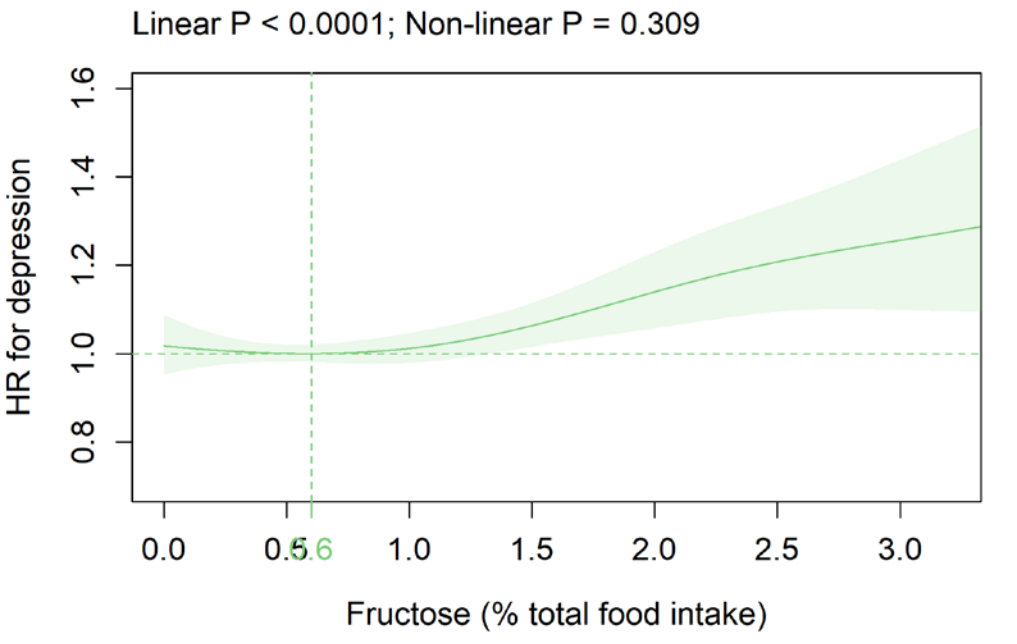

(v)

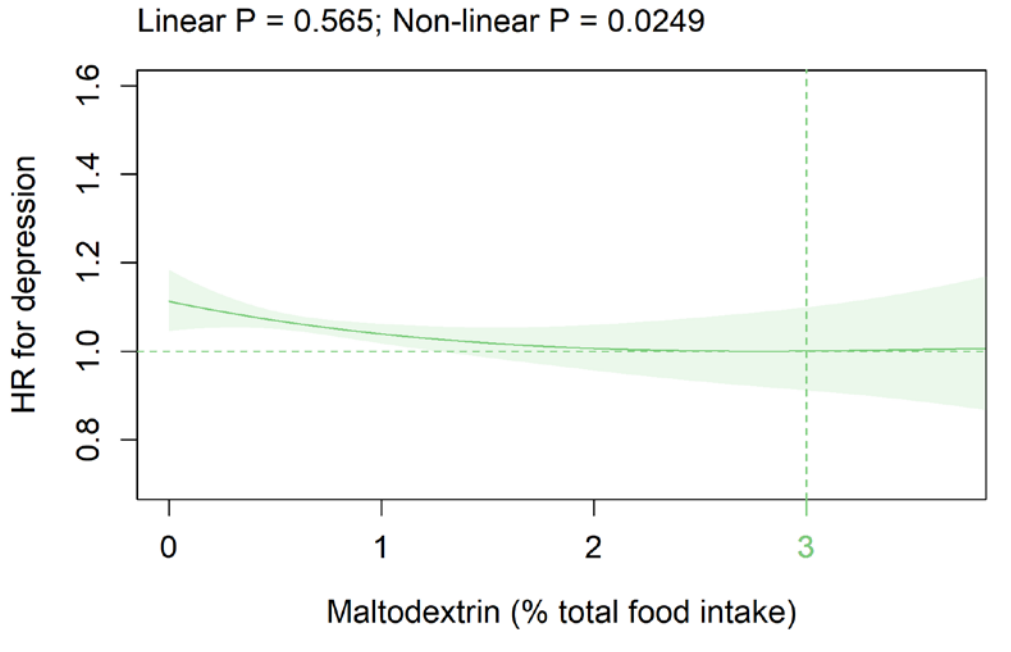

(w)

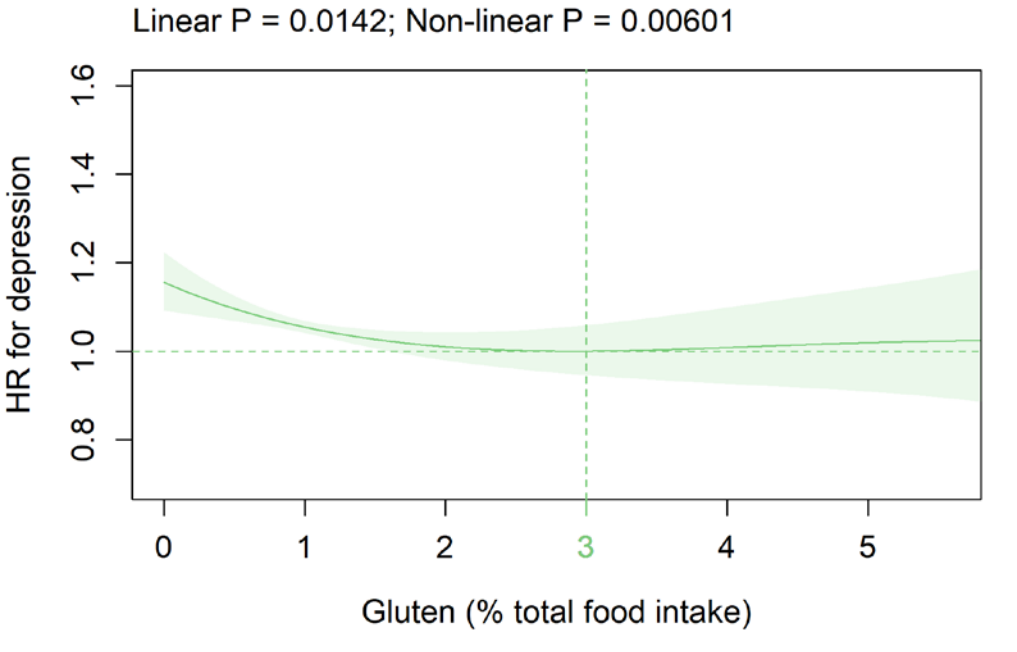

Online Resource 10 - History of CVD and cancer excluded

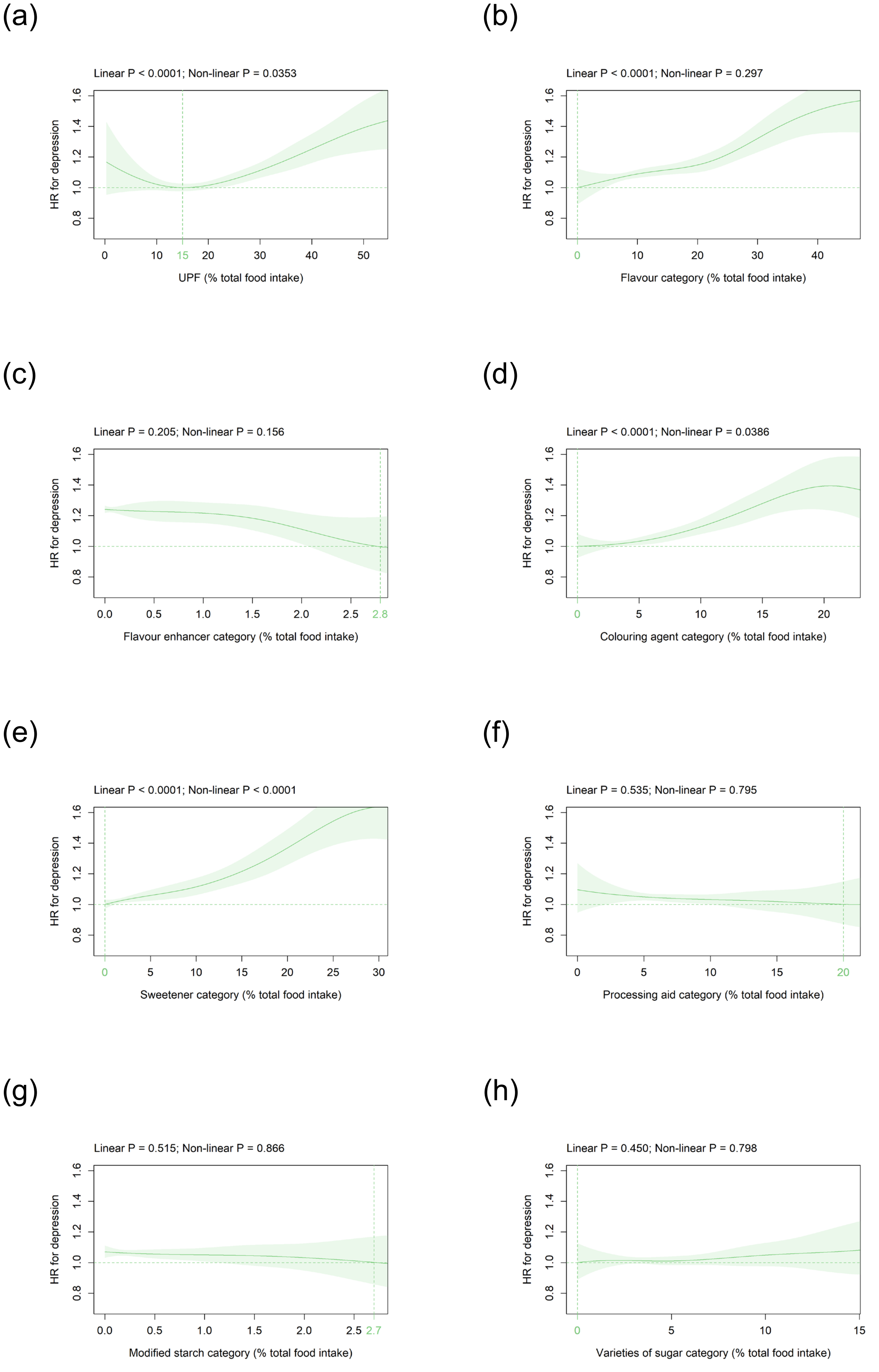

Online Resource 11 - First Oxford WebQ only

(i)

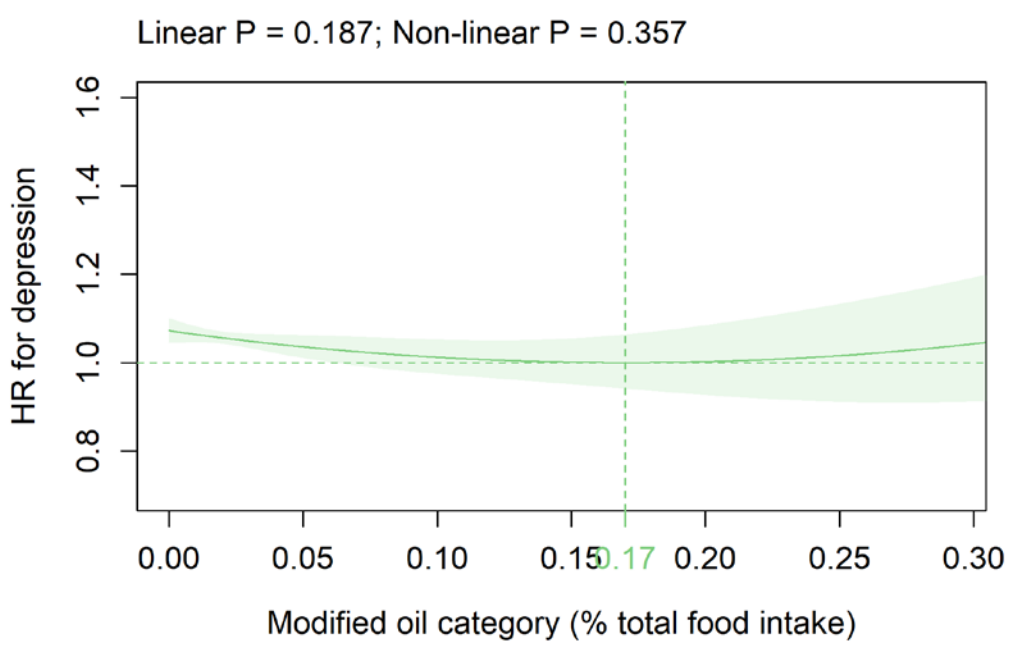

(j)

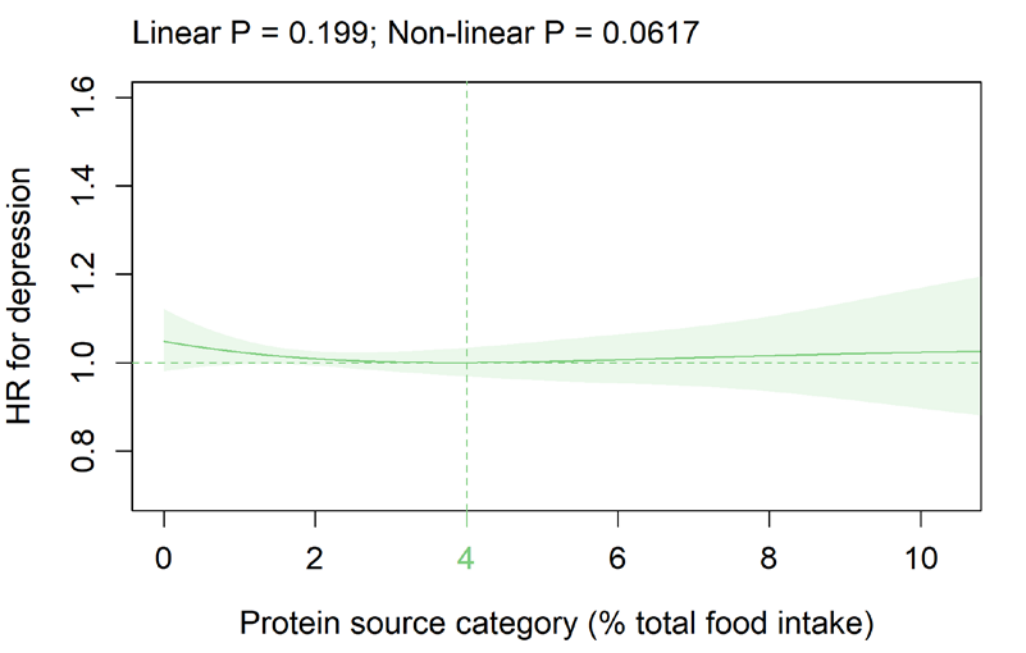

(k)

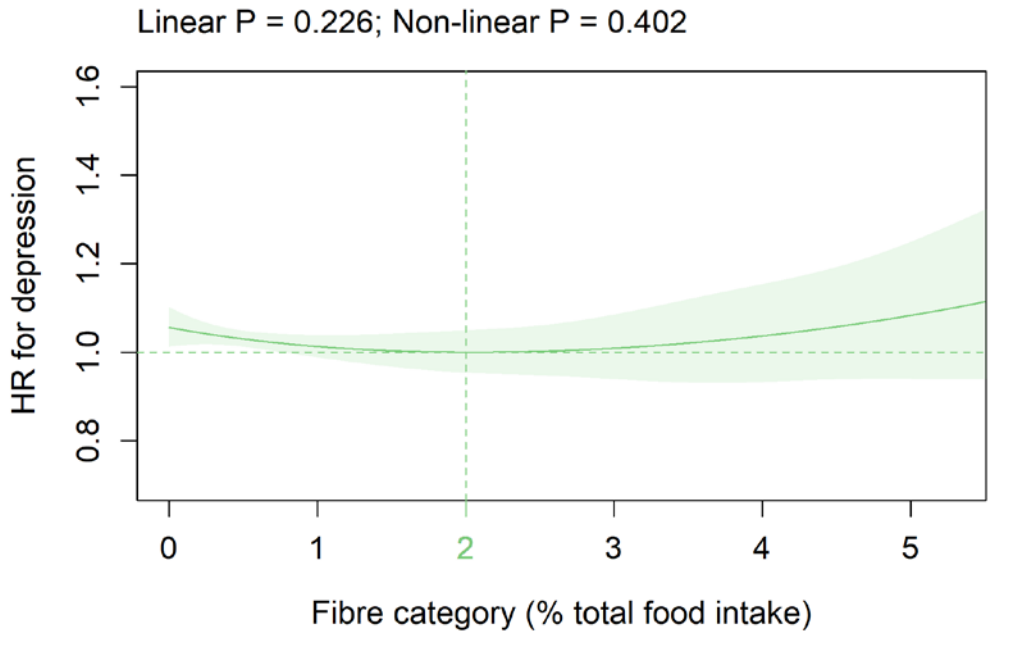

Online Resource 11 - First Oxford WebQ only

(l)

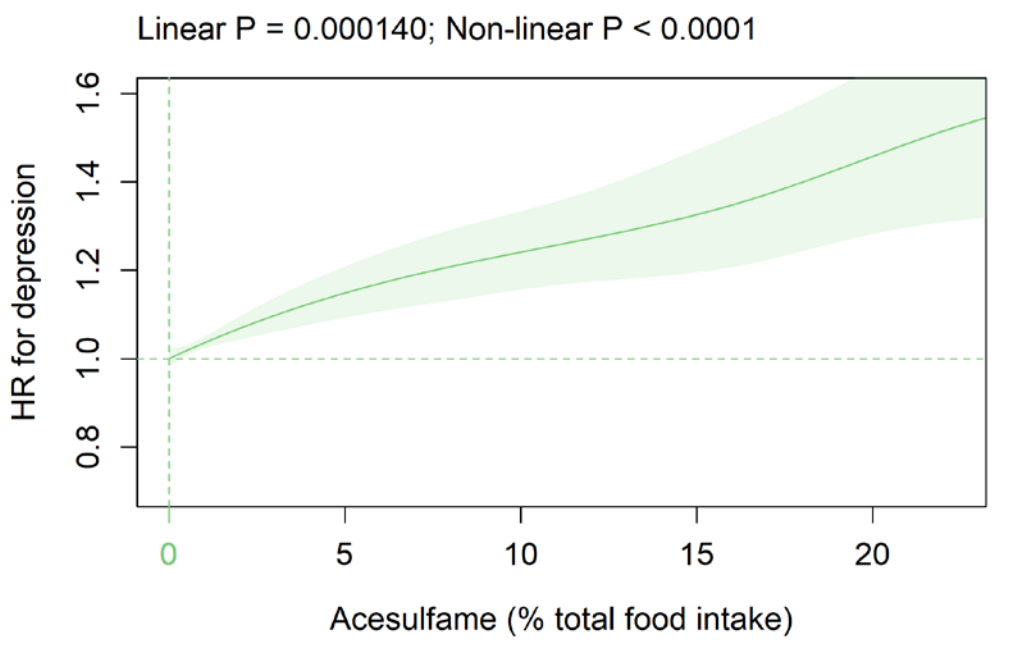

(m)

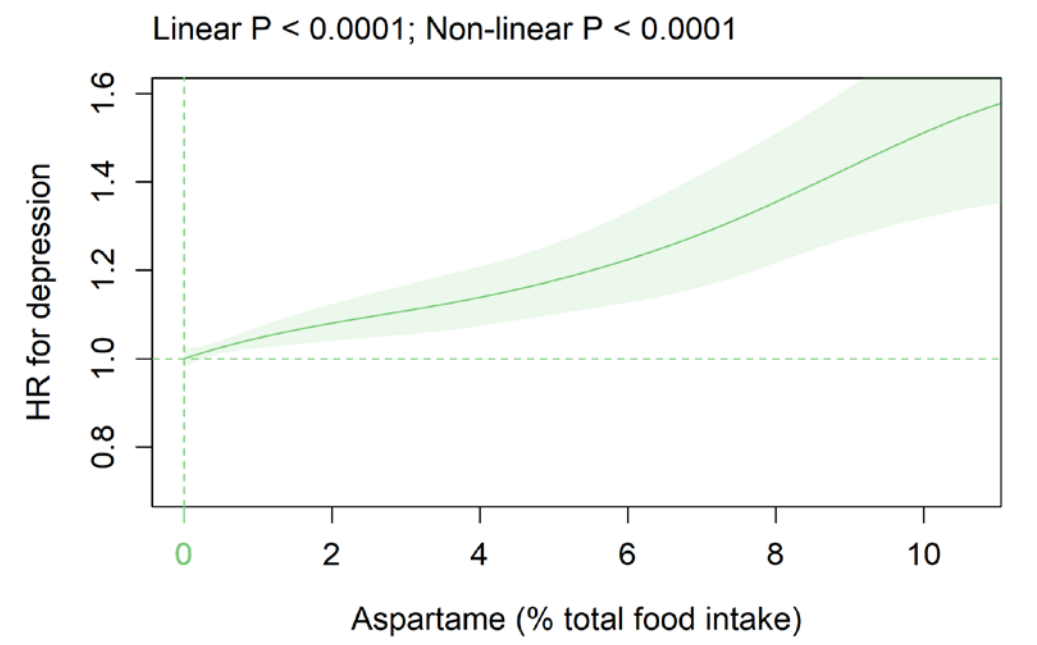

(n)

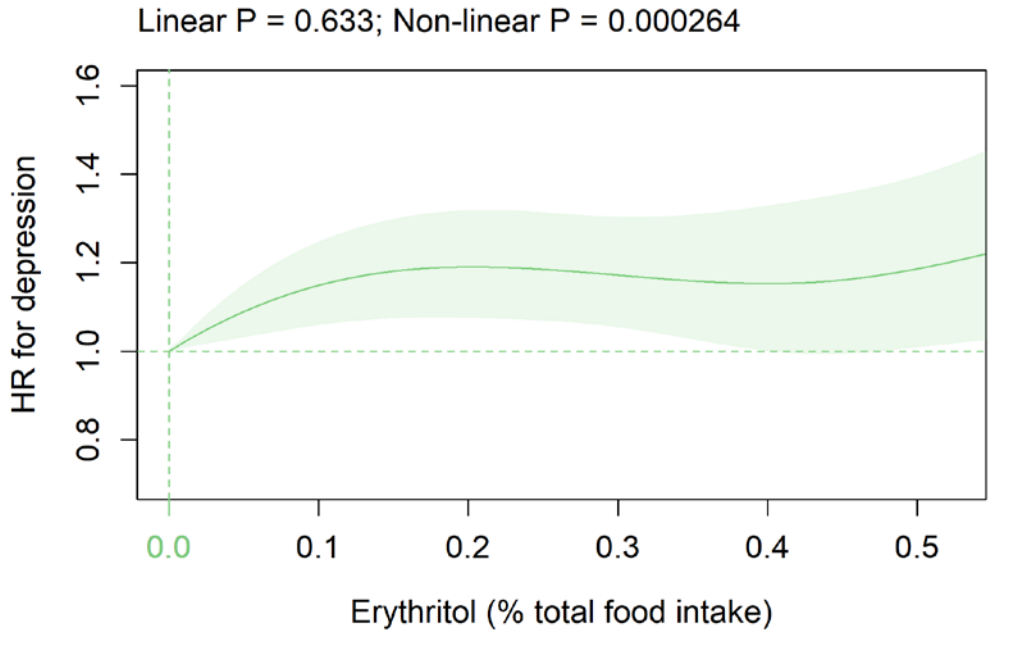

(o)

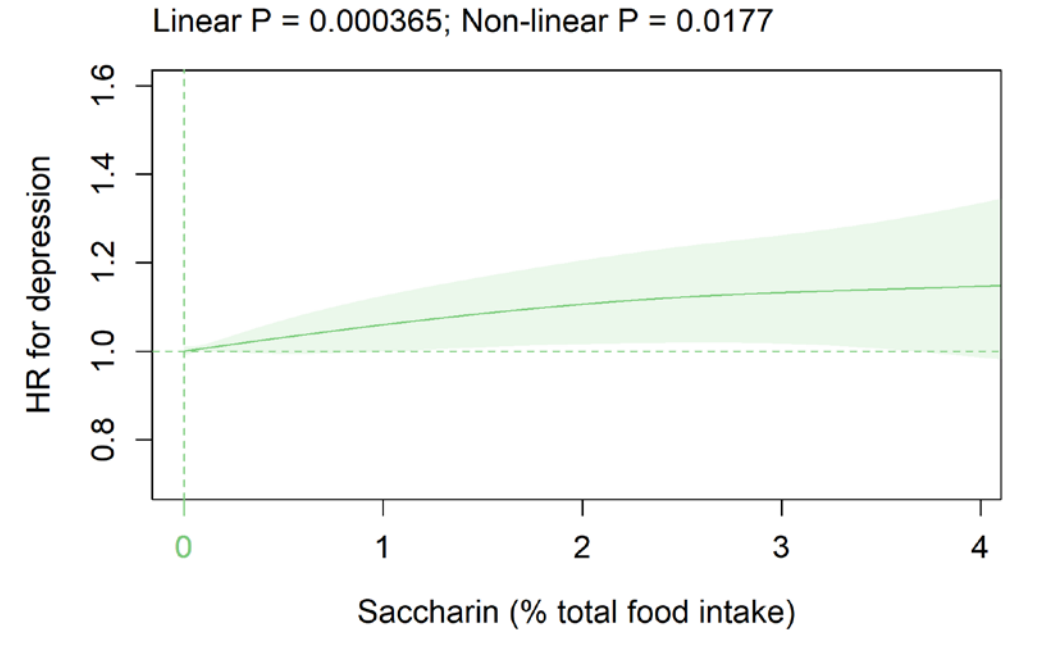

(p)

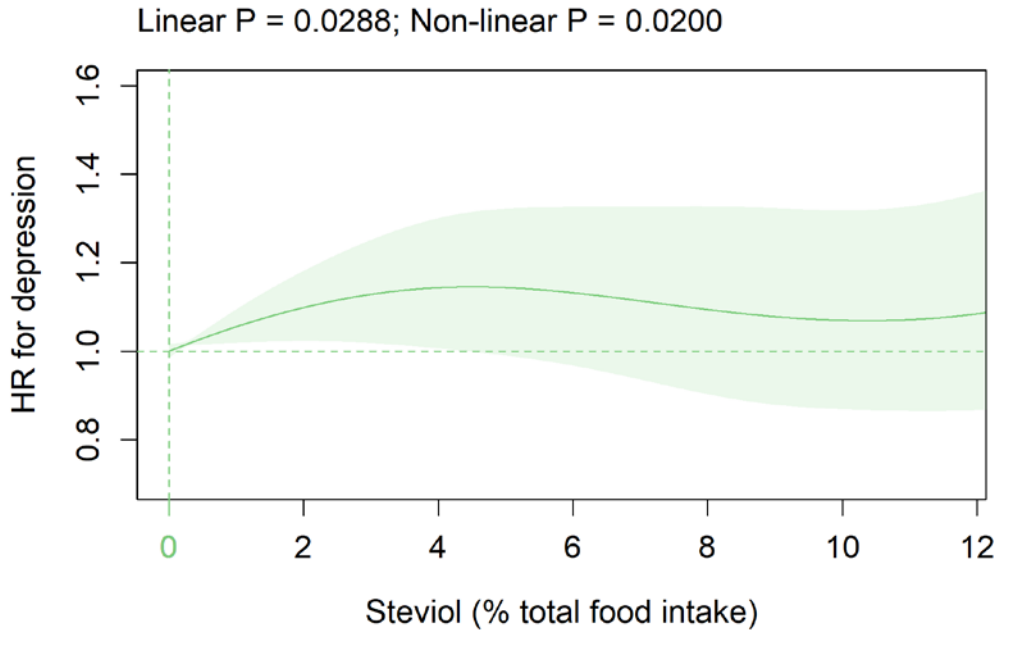

(q)

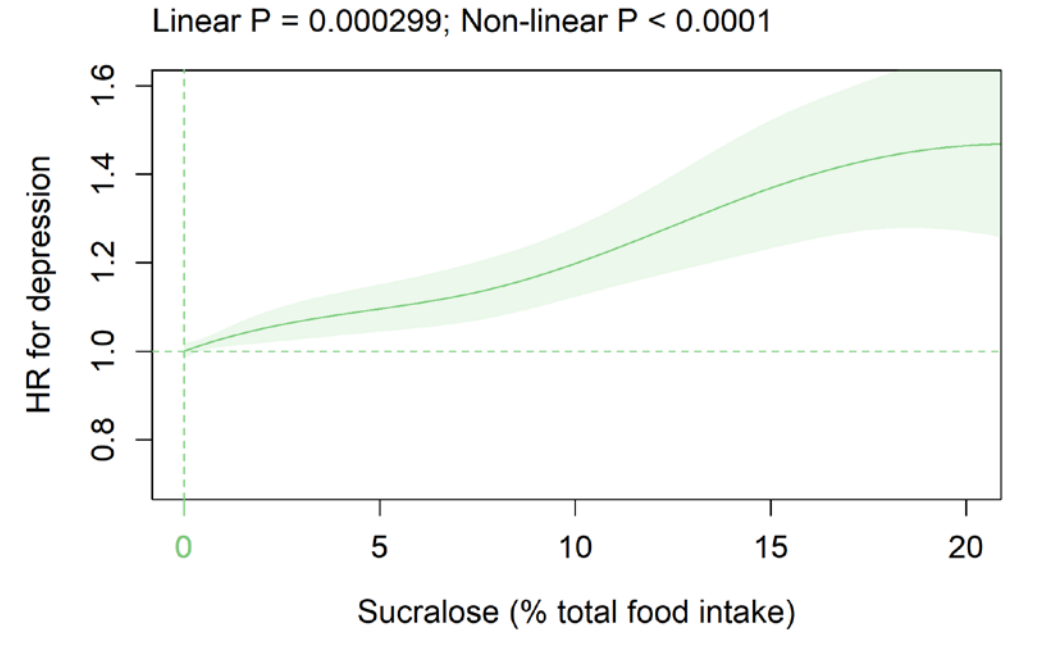

(r)

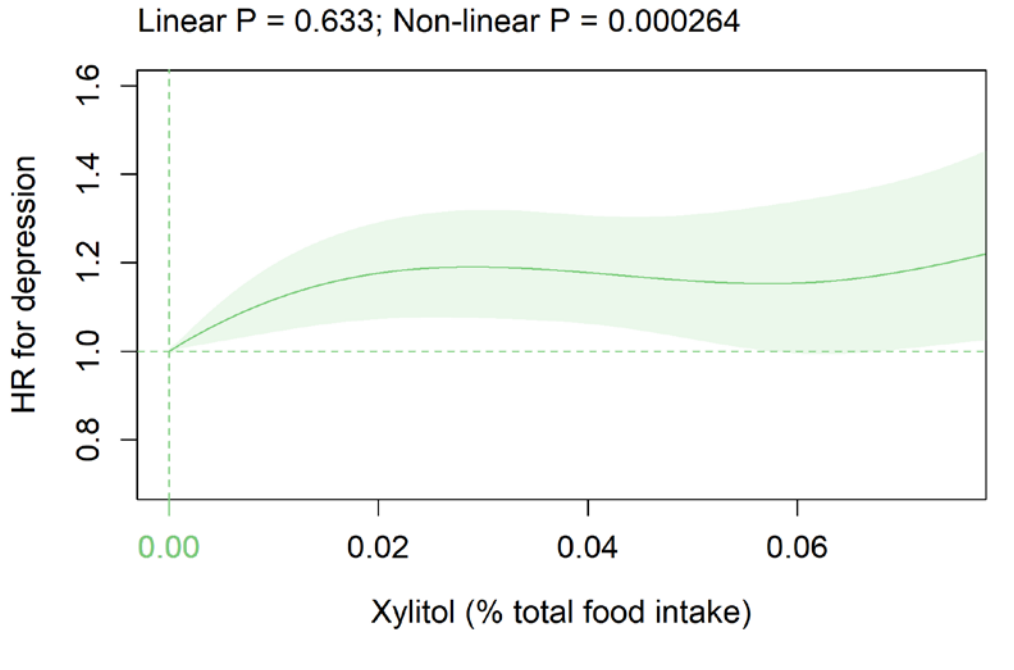

(s)

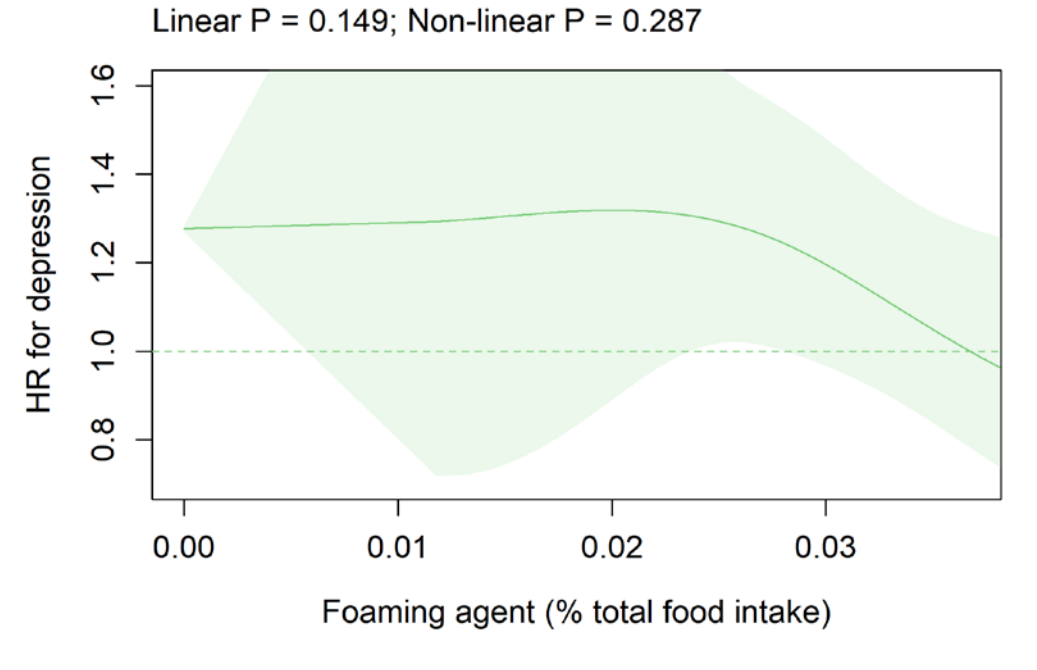

Online Resource 11 - First Oxford WebQ only

(t)

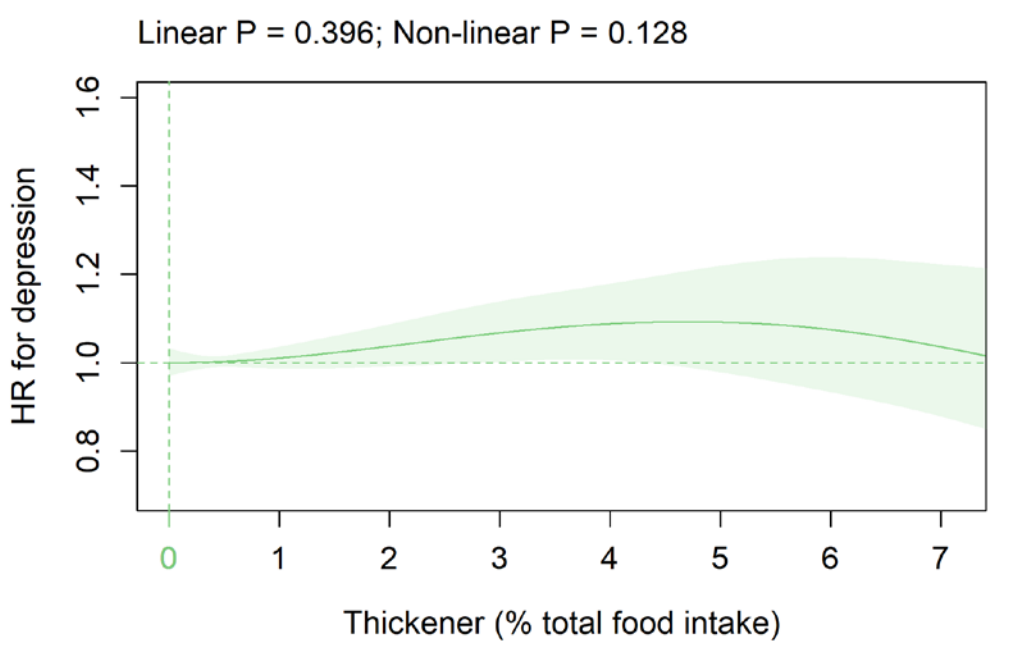

(u)

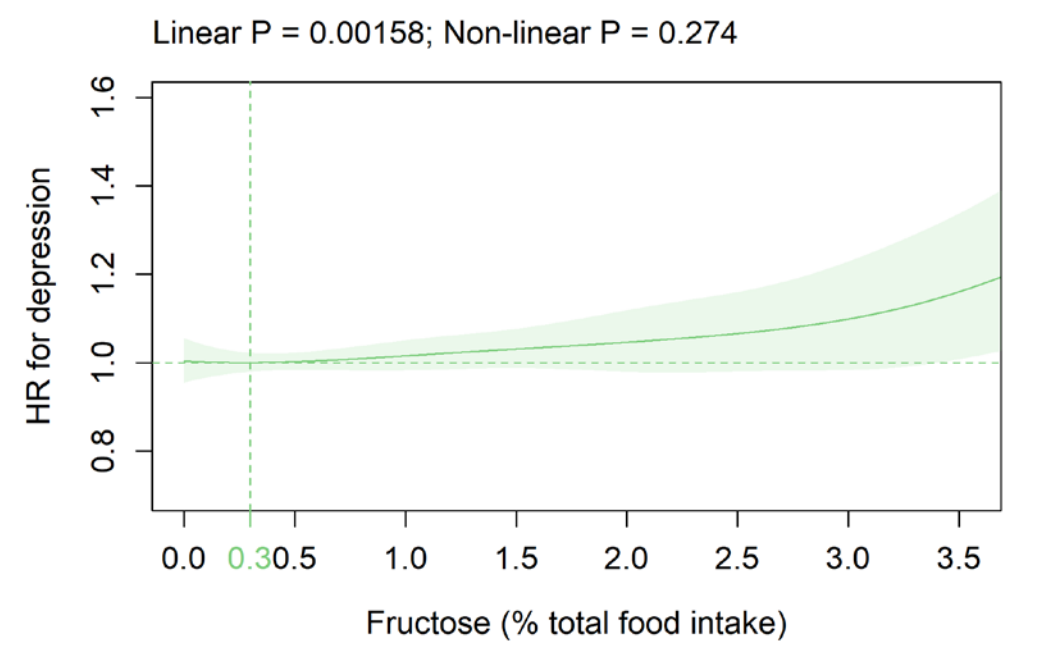

(v)

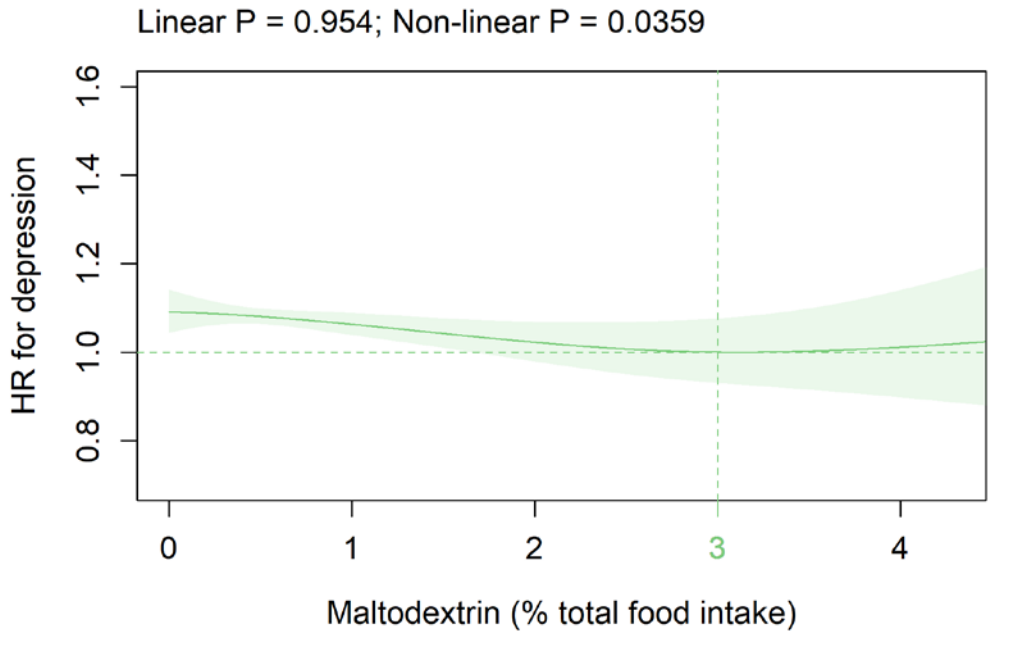

(w)

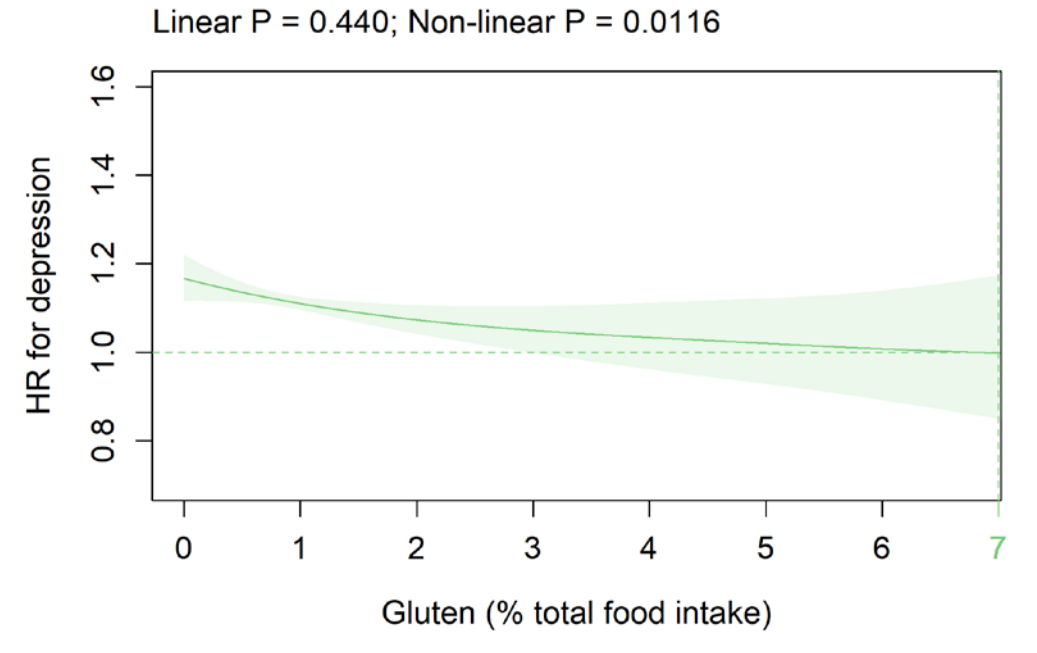

Online Resource 11 - First Oxford WebQ only

Online Resource 15. Association of MUP categories and specific MUPs with incident depression in the main and sensitivity analyses further adjusted for multiple testing using the Benjamini-Hochberg method for the MUP categories and specific MUPs separately. Results with an FDR-adjusted P<0.05 were considered as statistically significant and are only shown for associations which were significant in the main analyses without adjustment for multiple testing.[a]

		Main analysis	Landmark analysis	Unintentional weight loss removed	Atypical diet excluded	More than one Oxford WebQ	History of CVD and cancer excluded	First Oxford WebQ only	Further adjusted for diet quality	WHR and height instead of BMI	Energy intake removed
	Online Resource		6	7	8	9	10	11	12	13	14
Category											
- Flavour	b	s	s	s	s	s	s	s	s	s	s
- Colouring agent	d	s	s	s	s	s	s	s	s	s	s
- Sweetener	e	s	s	s	s	s	s	s	s	s	s
- Protein source	j	ns	s	ns	s	ns	ns	ns	ns	ns	ns
- Fibre	k	ns	ns	ns	ns	s	ns	ns	ns	ns	ns
MUP											
- Acesulfame	l	s	s	s	s	s	s	s	s	s	s
- Aspartame	m	s	s	s	s	s	s	s	s	s	s
- Erythritol	n	s	s	s	s	ns	s	s	s	s	s
- Saccharin	o	s	ns	ns	s	s	ns	s	s	s	ns
- Steviol	p	s	s	s	ns	ns	ns	ns	s	s	ns
- Sucralose	q	s	s	s	s	s	s	s	s	s	s
- Xylitol	r	s	s	s	s	ns	s	s	s	s	s
- Foaming agent	s	ns	ns	ns	s	ns	ns	ns	ns	ns	ns
- Thickener	t	ns	ns	ns	s	ns	s	ns	ns	ns	ns
- Fructose	u	s	s	s	s	ns	s	s	s	s	s
- Maltodextrin	v	ns	ns	ns	s	ns	ns	ns	ns	ns	ns
- Gluten	w	s	s	ns	s	s	s	ns	s	s	s

[a]The respective Online Resources are indicated (e.g., Landmark analysis for colouring agent is depicted in Online Resource 6d). Abbreviations: BMI, Body mass index; CVD, Cardiovascular disease; FDR, False discovery rate; MUP, Marker of ultra-processed food; ns, Non-significant; s, Significant, UPF, Ultra-processed food; WHR, Waist-to-hip ratio
